# Ferrocene-Based Hybrid Drugs as Potential Anticancer and Antibacterial Therapeutic Agents for Incorporation into Nanocarriers: In Silico, In Vitro, Molecular Docking Evaluations

**DOI:** 10.3390/pharmaceutics17060722

**Published:** 2025-05-30

**Authors:** Sijongesonke Peter, Eric Morifi, Mutshinyalo Nwamadi, Samson Olaitan Oselusi, Asongwe Lioniel Tantoh, Thierry Youmbi Fonkui, Derek Tantoh Ndinteh, Blessing Atim Aderibigbe

**Affiliations:** 1 Department of Chemistry, University of Fort Hare, Alice Campus, Dikeni 5700, South Africa; 2 School of Chemistry, Mass Spectrometry Division, University of Witwatersrand, Johannesburg 2017, South Africa; eric.morifi@wits.ac.za; 3 Department of Chemistry, University of Johannesburg, Auckland Park Campus, Johannesburg 2017, South Africa; mnwamadi@uj.ac.za; 4 Department of Biotechnology, University of the Western Cape, Bellville, Cape Town 7535, South Africa; 3866891@myuwc.ac.za; 5 Pharmaceutical, Future Production Chemical Cluster, Council for Scientific and Industrial Research (CSIR), Meiring Naude Road, Brummeria, P.O. Box 395, Pretoria 0001, South Africa; atantoh@csir.co.za; 6 Department of Biotechnology, University of Johannesburg, Doornfontein Campus, Johannesburg 2092, South Africa; youmbifonkui@yahoo.com; 7 Department of Chemical Sciences, University of Johannesburg, Doornfontein Campus, Johannesburg 2092, South Africa; dndinteh@uj.ac.za

**Keywords:** hybrids, ferrocene, drug resistance, pharmacokinetics, anticancer, antibacterial

## Abstract

**Background/Objectives:** Cancer and bacterial cases are increasing. Hence, new drugs to treat these diseases are paramount. Ferrocene-based hybrid compounds were synthesizedas potential cancer and bacteria therapeutics. **Methods:** The synthesized compounds were characterized via FTIR, NMR, and LC-MS and evaluated against different cancer cells and bacterial strains. Moreover, computational studies of these compounds were conducted using several silico tools. **Results:** Among the synthesized compounds, hybrid **10** was the most promising compound, displaying promising anticancer activity with IC_50_ values between 42.42 and 45.37 and 50.64 and 73.37 µg/mL against HeLa and CHO cancer cells, respectively, with a selective index greater than one on HeLa cancer cells. Compounds **22**–**26** displayed promising antibacterial activity with a MIC value of 7.8125 µg/mL against most bacterial strains in vitro. The in silico results revealed that this compound has strong binding affinities for 4qtb, 3eqm, and 2w3l cervical cancer proteins, exhibiting binding energies of −7.3, −8.7, and 7.4 kcal/mol, respectively. Furthermore, hybrid **10** showed promising pharmacokinetics and drug-like properties, including high GI absorption, moderate water solubility, favoring the oral administration route, nontoxicity, and is a P-gp substrate. **Conclusions:** The findings obtained in this study illustrate that hybrid compounds are potential therapeutics that need to be explored. The compounds also contained functionalities relevant for incorporating into nanocarriers to improve their biological activities further. Therefore, further studies are recommended for the most effective compounds to reinforce these findings.

## 1. Introduction

Bacterial infections are fatal to human health, and so is cancer [1,2]. The movement of bacteria from underlying areas such as the mouth, intestine, and nose to regions such as the kidney, blood, lungs, and brain results in human illness and, in some cases, can be deadly [3,4]. The standard treatment of bacteria is the use of antibiotics. However, the increasing cases of bacterial infection are induced by bacteria developing resistance to the commonly used antibiotics [5]. Additionally, the overuse of antibiotics also leads to drug resistance, resulting in challenging bacterial infections that are difficult to treat [1,6,7]. Thus, human lives are compromised each year, and the number of deaths due to bacterial infections is estimated to increase by 2050 [8]. On the other hand, the infinite growth of normal cells due to gene mutations results in cancer tumors that metastasize when they spread to other body tissues, becoming lethal [9]. The increasing number of cancer cases is associated with the lack of effective treatments [10,11,12]. Notably, the common challenges of commercially available drugs for both bacterial infections and cancer include drug resistance, toxicity, poor bioavailability, and severe side effects, resulting in increasing cases or even deaths [13,14]. Hence, it is critical to develop effective treatments for these diseases.

**Figure 1:**
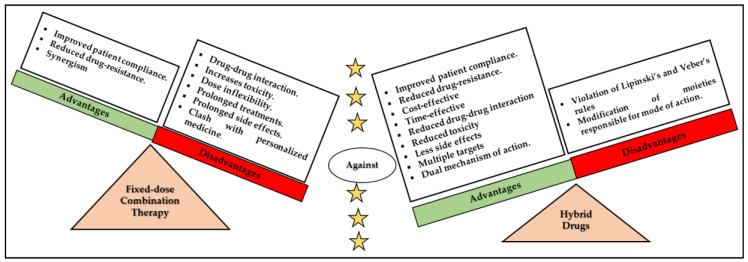
Benefits and limitations of the use of fixed-dose combination therapy and hybrid drugs [15,16,17,18,19].

**Figure 2:**
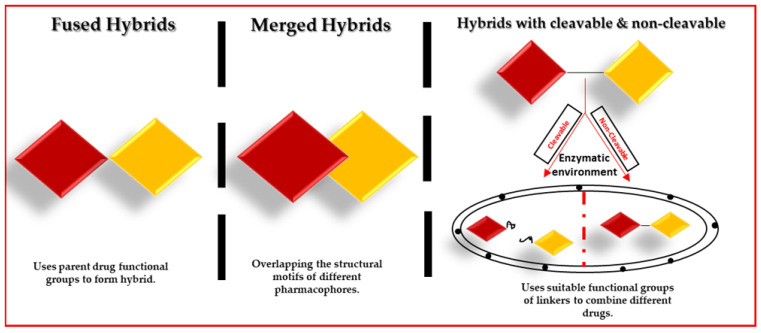
Different strategies to develop hybrid molecules [19,26].

Molecular hybridization via drug repurposing is gaining momentum from several scientists, and this approach is reported to be among the effective strategies for developing therapeutics [15]. This approach involves the formation of a single-entity drug from a cocktail of drugs [16]. This strategy has several advantages, including cost and time-effectiveness, reduced toxicity, and fewer side effects [1,15]. Among other combination strategies, this approach is reported to have more advantages, such as dual-targeting ability and reduced drug-drug interaction, with limited disadvantages, including alteration of the mode of action of the parental drug compared to the fixed-dose combination therapy (see Figure 1) [14,17,18,19]. Furthermore, using the already known and approved drugs to develop hybrid molecules is cost- and time-effective [20]. Hence, the development of hybrid drugs (see Figure 2) using known drugs must be explored [15,17,19]. The literature that has been documented on most of the compounds employed in this study [16,21,22,23,24,25] has reinforced the use of these compounds in developing effective therapeutic agents, as the hybrids displayed promising biological activity compared to the free drugs. These findings revealed the efficacy of hybrid compounds for cancer and bacterial infection treatment. However, selecting appropriate linkers is vital for their pharmacokinetics in the design of hybrid compounds.

Ester and amide linkers in the design of hybrid drug molecules are classified as cleavable linkers [19,26]. The advantage of the cleavable linkers is their ability to efficiently cleave off under enzymatic conditions, resulting in dual or multiple targeting compared to non-cleavable linkers (see Table 1) [14,15,17,19]. Hence, the ester- and amide-linked hybrids approach was employed in this research. Apart from the efficacy of employing appropriate linkers in designing hybrid compounds, plant-based bioactive agents are promising precursors for developing hybrid compounds.

Utilizing plant-based bioactive compounds to develop hybrid compounds is a practical approach, and most approved medications are derived from plants [28,29]. Plant-based compounds, such as essential oils and triterpenoids, display several biological activities, including anticancer, antibacterial, antifungal, and antiviral activity [16,30,31]. However, their efficacy is compromised by some limitations, such as poor bioavailability and solubility [16]. Hence, hybridizing them with good potentiating agents such as a ferrocene moiety to enhance their anticancer activity is a promising approach. This study aimed to synthesize hybrid compounds containing ferrocene scaffolds and other selected pharmacophores, especially plant-based compounds, to develop potential anticancer and antibacterial agents. Notably, the synthesized ferrocene-based hybrids were characterized and evaluated in vitro for their anticancer and antibacterial activity against cancer cell lines and bacterial strains.

**Figure 3:**
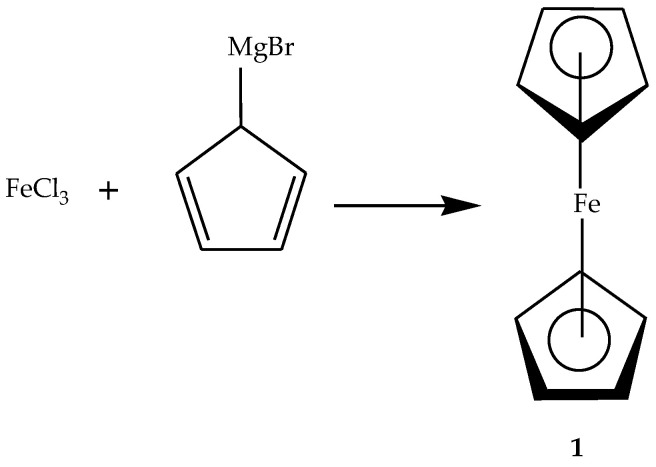
Synthetic route for ferrocene scaffold [33,34].

Ferrocene (displayed in Figure 3) is one of the organometallics reported to enhance the biological efficacy of other drugs [32,33]. The chemical versatility, good lipophilic nature, and stability (stable in water, air, and light) are the highlighted characteristics that triggered its hybridization with other pharmacophores [32,33]. Its combination with other organic molecules results in novel compounds with multiple biological activities [32,33]. Thus, several researchers have explored the use of ferrocene to develop hybrid compounds with numerous biological activities.

**Figure 4:**
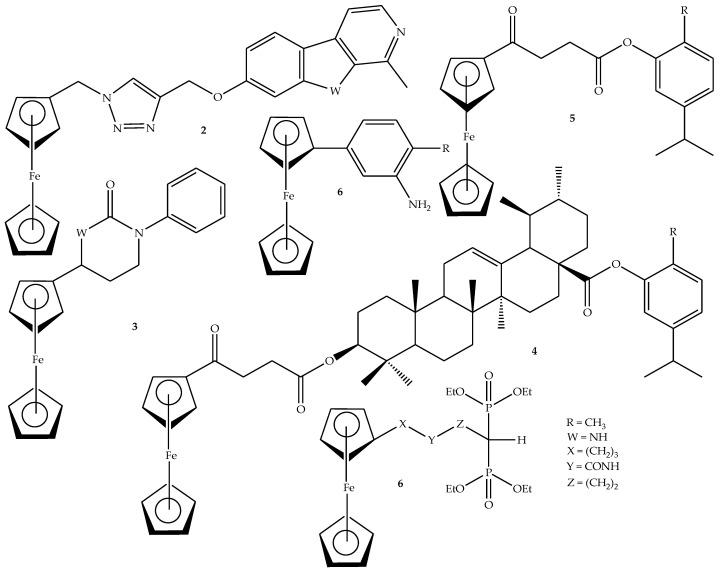
Chemical structures of the reported ferrocene-based hybrids (**2**–**7**).

Poje et al. published a ferrocene-harmine-based hybrid, **2** (Figure 4), that exhibited significant and selective antiproliferative activity against the MCF-7 and HCT116 cell lines. The hybrid compounds induced G1 cell cycle arrest followed by G2/M arrest [34]. Jančić et al. reported ferrocene-based hybrids’ antioxidant and antimicrobial activity [35]. The hybrids were more effective in neutralizing ABTS^·+^ than DPPH^.^ The most potent compound, **3** (depicted in Figure 4), contained tetrahydropyrimidin-2(1*H*)-ones and displayed antioxidant properties that were comparable with an analog of vitamin E, Trolox. The compounds exhibited moderate or weak antibacterial activity on the bacterial strains, *P. aeruginosa*, *E. coli*, and *S. aureus*, but selective activity against fungal species (i.e., *D. stemonitis*, *P. canescens*, and *F. oxysporum*) [35]. The findings from Khwaza et al. revealed the potency of ursolic acid–ferrocene hybrid compound, **4** (Figure 4), which was effective against bacterial strains with MIC values of 15.625 μg/mL and displayed promising cytotoxic effects against human cancer cells (i.e., MDA-MB-231, HeLa, and MCF-7) [36]. Mbese et al. also reported results of a synthesized carvacrol-ferrocene hybrid compound, **5** (Figure 4), with promising cytotoxic effects and antibacterial activity. It displayed cytotoxic effects against the MDA-MB-231, MCF-7, and DU147 cells and no effect against MCF-12A cells, indicating it is cytotoxic on non-cancer cell lines [37].

**Figure 5:**
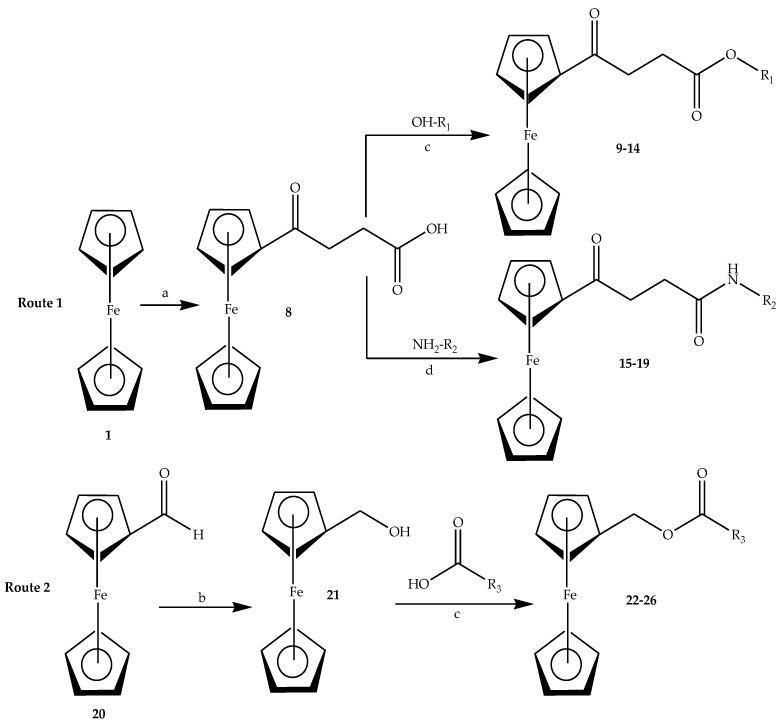
Synthetic routes (**1**–**3**) of hybrid (**9**–**26**) where a = C_4_H_4_O_3_ (succinic anhydride), AlCl_3_, DCM, 40 °C, 2 h; b = NaBH_4_, EtOH, rt, 2 h; c = THF/DMSO/DMF, DCC, DAMP, rt, 48 h; d = THF/DMSO/DMF, DCC, HSU, rt, 48 h.

Asghar et al. documented synthesized ferrocene-functionalized aniline with significant free radical scavenging activity against DPPH with IC_50_ values of 15.9–31.5 µM. Their anticancer activity was moderate against MCF-7. However, their fewer toxic effects on normal cells (MCF-10A) were noted [38]. Anusionwu et al. reported a ferrocene–bisphosphonate hybrid compound, **6** (Figure 4), with significant antibacterial activity against all the bacterial strains used in the study compared to fosfomycin with the lowest MIC value of ≤3 against *Proteus mirabilis* and *Klebsiella aerogenes*. Their antifungal activity against *Penicillum citrinum* and *Aspergillus ochraceus* was comparable to that of the control, nystatin [39]. The above reports validate the use of ferrocene to develop novel and effective drugs with multiple biological activities. Some reports highlighted the effects of the type of substituents, length, type of linker, and the introduction of a ferrocene moiety on the biological activity of the synthesized hybrid compounds [34,35,38]. Hence, this study synthesized different hybrid molecules containing ferrocene scaffolds via a general synthetic procedure depicted in Figure 5.

Developing new drugs is costly and time-consuming, and using in silico software to predict the physicochemical properties of the molecules at the early stages of drug development is crucial [40,41]. The in silico approach involves the virtual screening of different chemical molecules and predicting their toxicology and physicochemical properties. It increases the probability of discovering novel therapeutic agents at a low cost and in less time. Importantly, this approach complements the development of new drugs in less time and at a lower cost [40,41]. In silico studies have been used in a previous study to predict the physicochemical properties of newly synthesized ferrocene hybrid compounds [39]. This study employed free in silico online software to predict the potential outcomes of these ferrocene-based hybrid compounds.

## 2. Materials and Methods

### 2.1. Apparatus

N,N′-dicyclohexylcarbodiimide (DCC), 4-dimethylaminopyridine (DMAP), hydroxysuccinimide (HSU), ferrocene, ferrocene carboxaldehyde, carvacrol, pyrimethamine, metformin, curcumin, aluminum chloride (AlCl_3_), (NaBH_4_), and thiamine were purchased from Merck Chemicals, Johannesburg, South Africa. Oleanolic acid, ursolic acid, cinnamic acid, temozolomide, chlorambucil, artesunate, methotrexate, thymol, ciprofloxacin, norfloxacin, and doxorubicin were supplied by DB Fine Chemicals, Johannesburg, South Africa. The solvents, dimethyl sulfoxide (DMSO), dichloromethane (DCM), acetone, dimethylformamide (DMF), tetrahydrofuran (THF), ethanol, methanol, ethyl acetate, toluene, hexane, and chloroform, used were of HPLC grade and supplied by SAINS Agencies and Merck Chemicals, Johannesburg, South Africa. Silica gel plates (TLC Silica gel 60 F254), Spectroline template UV-4NFW 365 nm/254 nm, and Minimax UV lamp (254 nm) lamps were purchased from Merck Chemicals, Johannesburg, South Africa. Human cervical carcinoma (HeLa), Chinese hamster ovary (CHO), and African green monkey kidney (VERO) cell lines were obtained from ATCC. Dulbecco’s modified Eagle Medium 1X (DMEM), Penicillin (100 U/mL)/Streptomycin (100 μg/mL), 0.25% Trypsin-EDTA, phosphate-buffered saline (PBS), and, fetal bovine serum (FBS) were purchased from Life Technologies (GIBCO; Paisley), UK. PrestoBlue^TM^ Cell Viability reagent was from Life Technologies (Invitrogen; Eugene, OR, USA).

### 2.2. General Procedure for the Synthesis of Ferrocene-Based Hybrids, **9**–**26**

**Figure 6:**
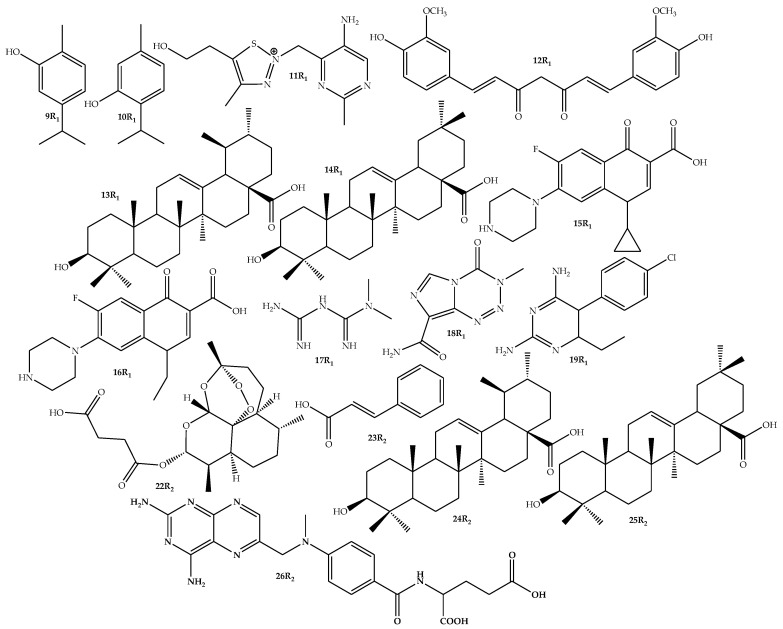
Chemical structure of the selected pharmacophores hybridized with ferrocene scaffolds. General synthesis of ester-linked hybrids via Route 1 (**9**–**14**) and Route 2 (**22**–**26**).

**Scheme 1:**
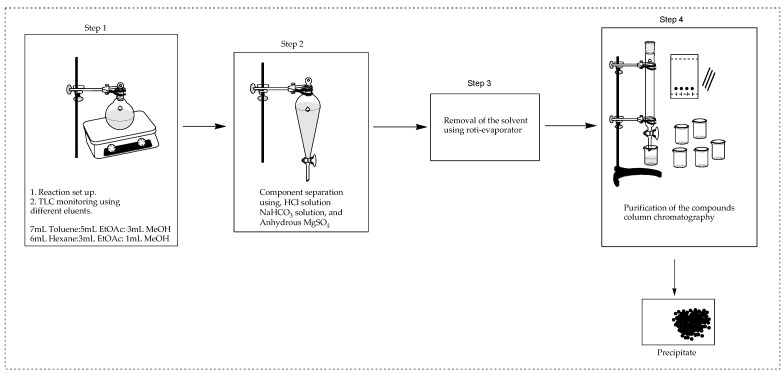
The schematic diagram illustrates the steps involved in synthesizing hybrids (**9**–**26**).

Hybrids **9**–**14** were synthesized via synthetic route 1. Ferrocene was modified into keto ferrocene butanoic acid following the procedure from our previous work by Peter et al. (Figure 5) [42]. The esterification procedure employed by Peter et al. [43] was used to hybridize keto ferrocene butanoic acid (0.6991 mmol) with either carvacrol (0.6991 mmol), thymol (0.6991 mmol), thiamine (0.6991 mmol), curcumin (0.6991 mmol), ursolic acid (0.6991 mmol), or oleanolic acid (0.6991 mmol) via the carboxylic acid (COOH) and hydroxyl (OH) functional groups, resulting in hybrids **9**–**14**. The compounds hybridized with ferrocene are shown in Figure 6. Different organic solvents, including THF, DMF, DMSO, and DCC, were used to dissolve the compounds. DMAP (0.6991 mmol) and DCC (0.7690 mmol) were used as coupling reagents and catalysts, respectively. Thin-layer chromatography was used to monitor the completion of the reaction using the eluent, toluene-ethyl acetate-methanol (7:5:3), or hexane-ethyl acetate-methanol (6:3:1) as displayed in Scheme 1. After reaction completion, 10 mL of a solution of sodium hydrogen carbonate (50 mg in 50 mL) and hydrochloric acid (1 mL of HCl in 500 mL of water) were added to the reaction and extracted three times by DCM using a separating funnel. Column chromatography was used for purification using an eluent, toluene-ethyl acetate-methanol (7:5:3), or hexane-ethyl acetate (6:3), followed by characterization using FTIR, NMR, and LC-MS, respectively.

**Figure 7:**
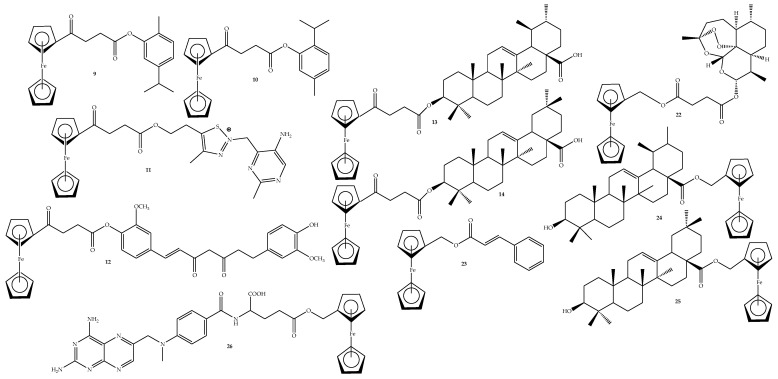
Chemical structures of the synthesized ester-linked ferrocene-based hybrid compounds (**9**–**14** and **22**–**26**).

To synthesize hybrids **22**–**26**, synthetic route 2 was applied (Figure 7). Firstly, ferrocene carboxaldehyde was reduced to methylferrocenol using the procedure adopted by Alkhan et al. and Singh et al. [42,44], followed by the hybridization of methylferrocenol with either artesunate (0.4629 mmol), cinnamic acid (0.4629 mmol), ursolic acid (0.4629 mmol), oleanolic acid (0.4629 mmol), or methotrexate (0.4629 mmol) via an esterification reaction through the COOH and OH groups. Thin-layer chromatography was used to monitor the completion of the reactions, and column chromatography was used to purify the hybrids. The compounds were characterized using FTIR, NMR, and LC-MS.

### 2.3. General Synthesis of Amide-Linked Hybrids via Route 1 (**15**–**19**)

**Figure 8:**
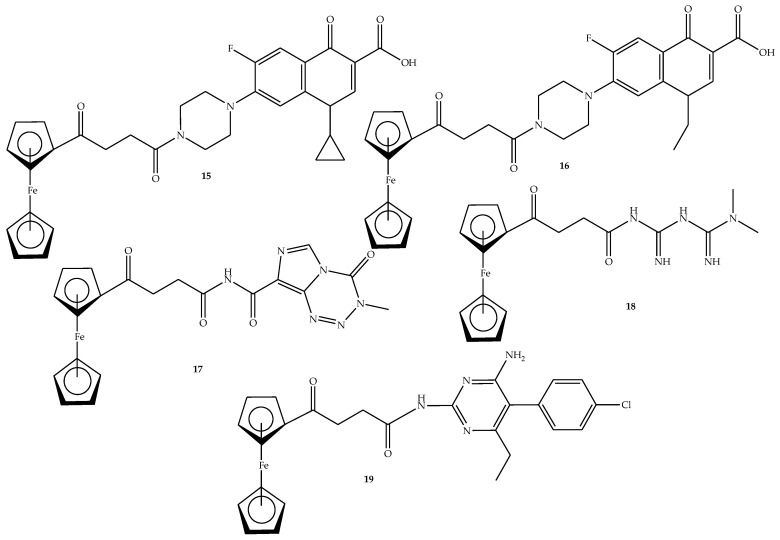
Chemical structures of the synthesized amide-linked ferrocene-based hybrid compounds (**15**–**19**).

Hybrids **15**–**19** were synthesized via synthetic route 1 (Figure 8). Ferrocene was modified into keto-ferrocene butanoic acid following the procedure from our previous work [42]. The amidation procedure also adopted from our previous work [44] was used to hybridize keto-ferrocene butanoic acid with either ciprofloxacin (0.6991 mmol), norfloxacin (0.6991 mmol), temozolomide (0.6991 mmol), metformin (0.6991 mmol), or pyrimethamine (0.6991 mmol) via the amine (N-H) and COOH group, resulting in hybrids **15**–**19**. DCC (0.7690) mmol) was used as a catalyst with HSU (0.6991 mmol), replacing DMAP as a coupling reagent. Different organic solvents were employed to dissolve the compounds, as depicted in Figure 5. Thin-layer chromatography was used to monitor the completion of the reaction using an eluent, toluene-ethyl acetate-methanol (7:5:3) or hexane-ethyl acetate-methanol (6:3:1), as shown in Scheme 1. After reaction completion, 10 mL of a solution of sodium hydrogen carbonate (50 mg in 50 mL) and hydrochloric acid (1 mL of HCl in 500 mL of water) were added to the reaction and extracted three times by DCM using a separating funnel. Column chromatography was used for purification using an eluent toluene-ethyl acetate-methanol (7:5:3) or hexane-ethyl acetate (6:3). FTIR, NMR, and LC-MS characterized the compounds.

These reported hybrid compounds are novel. However, hybrids **9** and **12** were evaluated for their anticancer, antibacterial, and antimalarial activity from the previous studies from our group by Mbese et al. [37] and Peter et al. [43], respectively. Hybrid **9** displayed moderate activity against breast cancer cell lines and no significant activity on bacterial strains. Moreover, compound **12** exhibited no significant antimalarial activity against *P. falciparum*. Therefore, this study is about hybridization and drug repurposing; hence, these compounds were included in this study to test them on different biological parameters.

### 2.4. Biological Evaluation

#### 2.4.1. In Vitro Antibacterial Evaluation

The MIC of the compounds studied was determined using the protocol reported by Fonkui et al. [45]. Each compound was dissolved in distilled water to make a stock solution of 1 mg/mL concentration for the test compounds. These solutions were then serially diluted (6 times) in 100 µL of nutrient broth in 96-well plates to the desired concentrations of 500, 250, 125, 62.5, 31.25, and 15.625 µg/mL. Then, 100 µL of each solution was placed in duplicate and seeded with 100 µL of an overnight bacterial culture brought to 0.5 McFarland in nutrient broth. Streptomycin, ampicillin, and nalidixic acid were used as positive controls, and a negative control contained 50% nutrient broth in DMSO.

#### 2.4.2. In Vitro Anticancer Evaluation

Stock solutions of 5 mg/mL concentration were prepared using DMSO or chloroform/methanol. The stock solutions were sonicated, followed by serial dilution by adding DMEM containing 10% FBS. The solutions were filtered using a 0.45 µm filter.

Cell culture was performed at 37 °C in a humidified incubator with 5% CO_2_. A 75 cm^2^ tissue culture flask containing DMEM media supplemented with 10% heat-inactivated FBS and 1% antibiotics (100 U/mL penicillin, 100 μg/mL streptomycin) was used. Cell harvesting was carried out at 70–80% cell confluency using trypsin-EDTA (0.25% trypsin plus 0.01% EDTA) with a subculture in the complement culture media. The cell lines were cultured in full DMEM supplemented with 10% heat-inactivated FBS and 1% antibiotic/antifungal solution at 37 °C in an atmosphere of 95% air and 5% CO_2_. The cells were treated with the test samples at 1.5625–200 µg/mL concentrations in a final 100 µL culture media for 24, 48, and 72 h. Phenylarsine Oxide (PAO) was selected as the positive control, and the negative control was the untreated cells. After each experimental time-point, media from each well was aspirated, and 90 µL of the fresh solution was added to each well, followed by 10 µL of the PrestoBlue reagent and incubated for 2 h (for Hela cells) and 3 h for both CHO and VERO cells at 37 °C in an atmosphere of 5% CO_2_. Fluorescence was read at Em 535 nm and Ex 612 nm using TECAN fluorescence, absorbance, and luminescence microplate reader. Percentage (%) cell viability was determined using the formula:

$$\%\;\mathrm{Cell}\;\mathrm{Viability}=\frac{Extract-Blank\;avaerage}{Cell\;control\;average-Blank\;average}\times 100$$

NOTE: Each experiment was repeated three times for various time points.

PrestoBlue solution was aspirated, and each plate was fixed in 4% *w*/*v* paraformaldehyde in PBS for 10 min, followed by treatment of cells for 10 min with 0.1 Triton X-100 (Sigma-Aldrich, St Louis, MO, USA) in PBS. Cell nuclei were counterstained with 1:1000 (10 µmg/mL) Hoechst 33,324 (Sigma-Aldrich, St Louis, MO, USA) in PBS. Culture plates were imaged using a Cytation3 cell imaging multi-mode plate reader from Bio-Tek (Winooski, VT, USA). DAPI (4′,6-diamidino-2-phenylindole)excitation 377 nm, emission 447 nm, and Bright Field channels were used to obtain separate images.

#### 2.4.3. In Silico Studies

SMILES images were generated using ChemDraw (ChemDraw Ultra 8.0 app), followed by the use of several free prediction online software tools, including SwissADME (http://www.swissadme.ch/, all accessed on 20 November 2024), ProTox-II (http://tox.charite.de/protox_II/), GUSAR (http://www.pharmaexpert.ru/GUSAR/antitargets.html), and cardiac toxicity (http://predherg.labmol.com.br/) to predict the toxicity, pharmacokinetics, pharmacodynamics, and administration of the drugs.

Moreover, the PyRx software program (version 0.8) was employed to perform the molecular docking of the molecules following a previously reported method [46]. The ligand structures were energy minimized with Universal Force Field (UFF) [47] using the conjugate gradient optimization algorithm, with the total number of steps set at 200. The Protein Data Bank (PDB) was used to retrieve human extracellular signal-regulated kinase (3poz), X-ray crystallographic structures of aromatase enzyme (4eqm), B-cell lymphoma-2 (BCL-2) (2w3l), and epidermal growth factor receptor (4qtb), through http://www.RCSB.org. UCSF Chimera software version 1.17.3 was used for docking. The PyRx program was used to convert proteins and molecules to ready-to-dock PDBQT format. Docking simulations were performed using the AutoDock Vina widget embedded within PyRx, employing the Lamarckian genetic algorithm [48]. The binding conformations with the lowest binding energy were selected for further analysis. The resulting protein–ligand interactions were visualized and analyzed using BIOVIA Discovery Studio Visualizer.

## 3. Results and Discussion

### 3.1. Characterization

The hybrid compounds were successfully synthesized via esterification for compounds (**9**–**14** and **22**–**26**) and amidation for compounds (**15**–**19**) reactions in good yields of 59–84% and melting points between 95–182 °C. These findings were comparable to the previously reported group from our group [49]. The characterization techniques, including FTIR, NMR, and LC-MS, also suggested that the hybrids were successfully synthesized. Notably, the FTIR signals confirming the presence of carbonyls (C=O) for both ester and amide carbon were visible between 1614 and 1739 cm^−1^, and the C-Fe signal was visible between 471 and 528 cm^−1^_,_ revealing the presence of a cyclopentadiene ring of ferrocene in the hybrid compounds. The C=O and C-Fe stretch were characteristic signals present in all the synthesized compounds, **9**–**19** and **22**–**26**. Moreover, the C-O stretch vibrations for esters were visible between 1210 and 1243 cm^−1^. The proton and carbon NMR spectra displayed the desired signals for the hybrid compounds for the carbons of the ester and amide linkages at 176.17–162.56 ppm (ester linker) for compounds **9**–**14** and **22**–**26,** and 173.13–154.68 ppm (amide linkers) with the proton spectra depicting amide linkers signals between 5.62 and 5.93 ppm for compounds **15**–**19**, illustrating the successful synthesis of the hybrids. Noteworthy, some NMR spectra were affected by the poor solubility of the hybrids, resulting in poor proton NMR spectra. However, the signals were similar to those reported in the literature [36,49,50]. The LC-MS spectra further confirmed the hybrids’ molecular weights, corresponding to the theoretical molecular weights with isotopic hydrogen detected in some compounds. Mbese et al. [37] also reported similar findings. The NMR and FTIR spectra are presented in the Supplementary File.

### 3.2. In Vitro Antibacterial Activity

The hybrids (**9**–**26**) were evaluated for their antibacterial activity against both Gram-negative and Gram-positive bacterial strains, including *Bacillus subtilis* (*BS*), *Enterococcus faecalis* (*EF*), *Mycobacterium smegmatis* (*MS*), *Staphylococcus epidermidis* (*SE*), *Escherichia coli*, (*E. coli*), *Enterobacter cloacae* (*EC*), *Klebsiella oxytoca* (*KO*), *Proteus vulgaris* (*PV*), *Pseudomonas aeruginosa* (*PA*), *Proteus mirabilis* (*PM*), *Staphylococcus aureus* (*SA*), and *Klebsiella pneumonia* (*KP*). Ampicillin, a commonly used drug to treat bacterial infections [51,52], was used as a positive control, with ketoferrocene butanoic acid (**8**) used as a negative control.

As depicted in Table 2, the hybrids displayed either a significant or comparable antibacterial activity as the controls, with MIC values between 7.8125 and 15.625 µg/mL compared to 26 µg/mL of ampicillin and 15.625 µg/mL of keto ferrocene butanoic acid scaffold 8, respectively, especially against Gram-negative bacterial strains. The ferrocene derivative, **8**, possessed antibacterial activity against several bacterial strains. However, against some bacterial strains such as *SE*, *SA*, and *MS*, most of the hybrids displayed poor antibacterial activity compared to ampicillin. Additionally, against the *Klebsiella pneumonia* bacteria strain, the compounds did not exhibit significant antibacterial effects with MIC values of either 125 or 250 µg/mL. Furthermore, the hybrids except compound **16** also showed poor antibacterial activity against *SE* bacterial strain in vitro. This illustrates that the compounds behave differently on various bacterial strains.

Noteworthy, both ester-linked and amide-linked hybrids (**9**–**19**) with keto ferrocene butanoic acid moiety exhibited better antibacterial activity (except hybrid **14**) compared to ampicillin and comparable antibacterial effect with keto ferrocene butanoic acid against *E. coli* which is one of the most common and resistant bacterial strains in humans [53], with MIC values of 15.625 µg/mL whereas the antibacterial activity of the hybrids (**22**–**26**) with methyl ferrocenol scaffold (**21**) displayed a compromised activity with MIC values of 250 µg/mL against the same bacterial strain, in vitro. This also illustrates the positive influence of the keto ferrocene butanoic acid scaffold compared to the methyl ferrocenol moiety on the antibacterial activity of these hybrids.

The hybrids (**22**–**26**) with methyl ferrocenol scaffolds displayed superior antibacterial activity compared to hybrids with keto ferrocene butanoic acid moiety, ampicillin, and compound **8** against most Gram-positive (*BS* and *EF*) and Gram-negative (*EC*, *PV*, *KO*, and *PA*), exhibiting MIC values of 7.8125 µg/mL. Notably, these hybrids were threefold superior to ampicillin and twofold superior to hybrid **9**–**19** against the bacterial strains in vitro. In essence, the hybrids displayed promising antibacterial activity against different bacterial strains. More studies, such as molecular docking, structural relationship activity, etc., are paramount to fully understanding their mode of action.

### 3.3. In Vitro Cytotoxicity Activity

The cytotoxicity effect of the sixteen synthesized hybrids was tested against different cell lines. POA and keto ferrocene butanoic acid (**8**) were used as a positive and negative control, respectively. The hybrids displayed an inferior cytotoxic effect compared to POA, as shown in Table 3. Noteworthy, two hybrids (**10** and **24**) displayed promising activity compared to keto ferrocene butanoic acid (100 µg/mL < IC_50_) against HeLa cells in a time-dose dependent manner with IC_50_ values between 42.42 and 83.97 µg/mL, in vitro. Compounds **10** and **13** exhibited appreciable anticancer effects compared to the parent compound, keto ferrocene butanoic acid.

Observably, hybrid **24** did not induce a cytotoxic effect against CHO cells, and hybrid **13** was not cytotoxic on the HeLa cell line. This illustrates the selective cytotoxic effects of the compounds on different cell lines. Similar findings were also highlighted by Khwaza et al. [36], Jadhav et al. [49], and Yan et al. [54] for hybrid molecules containing ferrocene scaffolds. The cytotoxic effect of ester-linked hybrids **10**, **13**, and **24** was significant compared to that of the amide-linked hybrids when evaluated against HeLa and CHO cancer lines, as the most effective compounds were those with ester linkers, and none from the amide-linked ferrocene hybrids displayed a significant cytotoxic effect. These observations demonstrate the impact of the ester linker on the cytotoxic effect of the hybrid compounds on different cancer cells. However, the influence was inconsistent for other compounds with ester linkers, suggesting that the nature of drugs used in the hybridization played a crucial role in their cytotoxic effect. Compound **10** contained carvacrol, while compounds **13** and **24** contained ursolic acid. The review paper previously reported by Peter et al. [16] on carvacrol and thymol hybrids and Khwaza et al. on ursolic-based hybrids [36] revealed the effect of the nature of drugs hybridized with either carvacrol or ursolic acid. However, more studies, such as the structural elucidation of these hybrids and their mechanism of action on these cancer cells, are needed to further validate the factors affecting these compounds’ cytotoxic effect.

**Figure 9:**
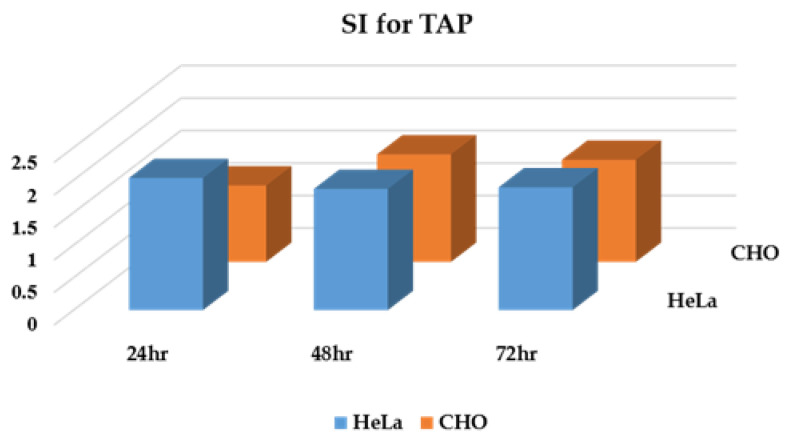
Selective index of hybrid **10** on HeLa and CHO cells.

Furthermore, against the normal cells (VERO), the hybrids displayed less than 20% tumor suppression, demonstrating their non-cytotoxic effect on the normal cells [55]. The selective indexes of hybrids **10**, **13**, and **24** are displayed in Figure 9. Hybrid **10** displayed a selective index greater than one on cancer cell lines, revealing its non-toxic effect on non-cancerous cells and suggesting this compound can be recommended for studies. Thus, hybrids **10** and **24** were cytotoxic against HeLa cell lines compared to other synthesized ferrocene-based compounds. A similar finding was reported by Skoupilova et al. for ferrocene-based derivatives against cervical cancer cells [56]. Hence, these two compounds were submitted for molecular docking studies to evaluate their binding potential on cervical cancer proteins.

### 3.4. Molecular Docking

**Figure 10:**
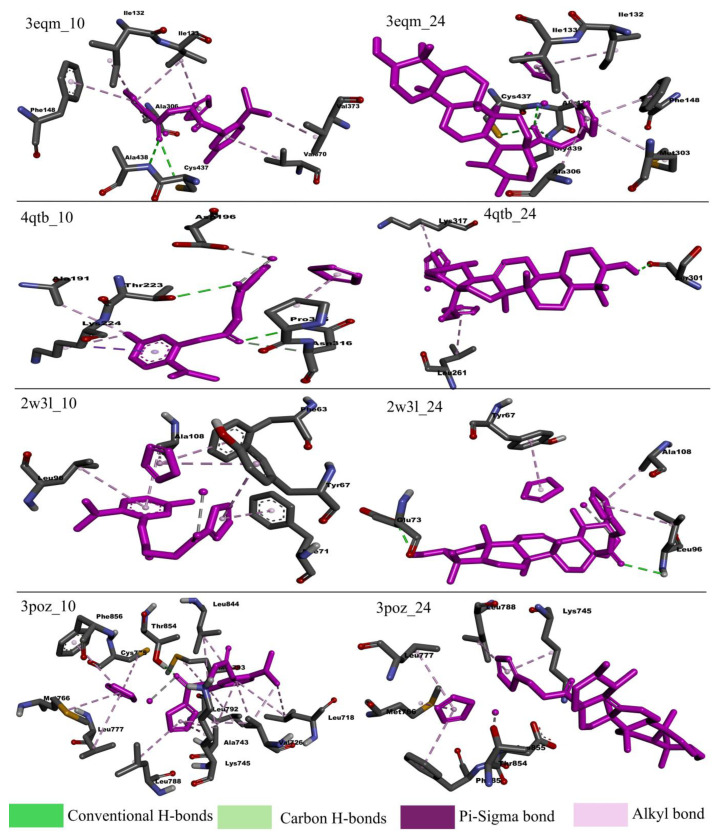
Three-dimensional interactions of the hybrids (**10** and **24**) with selected target proteins.

The interaction mechanism between amino acid residues and ligands plays a key role in many biological processes, accelerating advancements in drug discovery and design [57]. In this study, the BIOVIA Discovery Studio Client 2024 was used to analyze the docking results and visualize how selected anticancer proteins interact with the hybrid compounds at the molecular level. Table 4 and Figure 10 present the binding affinities and molecular interactions between selected cervical cancer protein targets and the hybrid compounds. Hybrid **10** and **24** interacted with 3eqm, achieving docking scores of −8.7 and −8.8 kcal/mol, respectively. Hybrid **10** formed two hydrogen bonds with Cys437 and Ala438, along with eight hydrophobic interactions involving Ile132, Ile133, Phe148, Ala306, Val370, and Val373. The complex, 3eqm_24 formed three hydrogen bonds with Cys437, Ala438, and Gly439, as well as 8 hydrophobic interactions with Ile132, Ile133, Phe148, Met303, Ala306, and Ala438.

The interactions between hybrid **10** and 3poz resulted in a docking score of −9.2 kcal/mol, forming one hydrogen bond with Thr854, and 18 hydrophobic interactions with Leu718, Val726, Ala743, Lys745, Met766, Cys755, Leu777, Leu788, Leu792, Leu844, and Phe856. The complex, 3poz_24, had a higher docking score of −11.0 kcal/mol, interacting with Lys745, Met766, Leu777, Leu788, and Phe856 via five hydrophobic bonds. 4qtb_10 and 4qtb_24 showed docking scores of −7.3 and −9.5 kcal/mol, respectively. 4qtb_10 formed three hydrogen bonds with Thr223 and Asn316, and four hydrophobic bonds with Ala191, Lys224 and Pro315, while 4qtb_10 formed one hydrogen bod with Ser301 and two hydrophobic interactions (Leu261, and Lys317). Finally, hybrids **10** and **24** interacted with 2w3l, with docking scores of −7.4 and 9.3 kcal/mol, respectively. Hybrid **10** further established seven hydrophobic interactions with Phe63, Tyr67, Phe71, Leu96, and Ala108, while 24 formed two hydrogen bonds with Glu73 and Leu96, and three hydrophobic interactions with Tyr67, Leu96, and Ala108. Overall, the compounds’ high negative docking scores and binding interactions indicated good binding affinity towards the biological targets, supporting the experimental results.

### 3.5. Toxicity and Swiss ADME Predictions

The tables revealing the ADME and toxicity predictions of the compounds are shown in Table 5, Table 6 and Table 7. Among the toxic side effects of the drugs used for treating bacterial infections and cancer is cardiac toxicity [25]. Hence, the hybrids predicted for HerG blockage. Some studies have predicted the capability of designed drug molecules to induce cardiac toxicity [58]. The hybrids were classified as blockers and non-blockers, as displayed in Table 4. The ester-linked hybrids with keto ferrocene butanoic acid moiety (**9**–**14**) were predicted as blockers, whereas the amide-linked hybrids (**15**–**19**) and ester-linked hybrid with methyl ferrocene moiety (**22**–**26**) were predicted to be non-blockers, except hybrid **19** and **26**, as displayed in Table 4. The hybrids predicted as blockers have a high probability of inducing cardiac failure. However, some compounds, including **10**, **11**, **13**, **16**, **19**, and **24,** were outside the applicable domain of the model. Hence, further studies involving the use of other models are recommended. Moreover, the compounds were predicted to be inactive against cytotoxic, carcinogenic, and mutagenic effects, as depicted in Table 7. This illustrates that not all the hybrids can promote gene mutation and are not harmful to normal cells.

**Figure 11:**
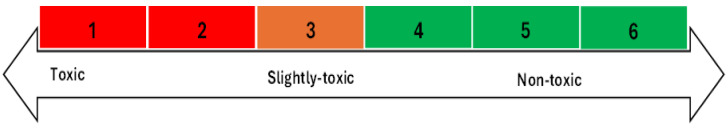
The toxicity classification [60,61].

Table 8 displays that the hybrids were predicted to be more appropriate for administration through the intraperitoneal and oral routes. The oral administration of drugs is the standard and most straightforward route due to its advantages, including cost-effectiveness and patient compliance [59]. Therefore, it is an ideal administration route that can be recommended for the synthesized hybrid compounds. Table 9 and Table 10 summarize the predicted pharmacokinetics and water solubility of the compounds, respectively. Hybrids **12**, **16**, and **17** were predicted to belong to class 3, which is slightly toxic (Figure 11). Hence, they are not recommended to be administered orally as they show toxicity via this route. Therefore, the intraperitoneal route can be recommended for these three compounds.

Furthermore, most of the compounds obeyed Veber’s rule for oral bioavailability as the topological polar surface area (TPSA) and rotatable bond (RB) were within the range with cutoff values of 140 and 10, respectively [62]. Additionally, several hybrids display no Lipinski’s rule violation, whereas some hybrids violated both MW and cLogP, displaying more than 500 Daltons and 5, respectively as displayed on Table 9 [63]. In addition, the hybrids were predicted to be P-gp substrates, which could improve their efficacy since P-gp substrates can enhance specificity and overcome multidrug resistance [64].

The GI absorption influences the oral bioavailability of the drugs [65]. Hybrids including **9**, **10**, **15**–**19**, **22**, and **23** were predicted to have high GI absorption (Table 10), making them more appropriate for oral route administration. This suggests these hybrids could be more effective when taken orally [39,66]. Furthermore, the low GI absorption of the hybrids was affected by their high molecular weights (MW), as high MW results in low GI absorption [39,67].

Most of the synthesized hybrids were predicted not to pass the BBB, except for hybrids **9**, **10**, and **23**. Their inability to pass BBB was compromised by their high TPSA values (more than 80 Å^2^) and molecular weights [68]. The inability of these hybrids to pass the BBB is an ideal characteristic illustrating that these hybrids may not cause neurotoxicity [69]. Furthermore, the majority of the synthesized hybrid compounds are predicted not to inhibit most of the CYP isozymes, and this suggests that the metabolism of these drugs could not be affected, as the inhibition of these enzymes may compromise their efficacy, cause toxicity, and lead to drug–drug interactions [39]. Therefore, these hybrids are good therapeutic agents, and more studies are recommended to reinforce these findings.

A drug’s efficacy is influenced by its bioavailability. Notably, poor bioavailability compromises the efficacy of the drugs [70]. The ideal bioavailability score for effective drugs has been reported to be between 0.55 and 1 [71]. Most of the synthesized hybrids displayed bioavailability scores (>0.55) within the ideal range, except for hybrids **24**, **25**, and **26**. Additionally, the oral bioavailability of the compounds is also influenced by the TPSA, which must be between 20 and 130 Å^2^ [72]. Hybrids **11** and **26** displayed TPSA that was not within the range, with values of 140.18 and 199.54 Å^2^, respectively. The rest of the hybrids displayed TPSA values between 26.30 and 140.18 Å^2^, demonstrating that most of the synthesized compounds will display optimal GI absorption. The high MW of compound **26** contributed to its poor TPSA. Similar findings were reported by Mbese et al. for hybrid compounds containing ferrocene scaffolds [60].

The hybrid compounds’ water solubilities were predicted, as shown in Table 11. The water solubility of a drug affects its bioavailability, with poorly water-soluble drugs exhibiting poor bioavailability and vice versa [70]. The synthesized hybrids, **17**, **18**, **22**, and **23,** were predicted to display good water solubility, while most of the hybrids were predicted to be either moderately or poorly soluble, as depicted in Table 10. A Log *P*o/w (iLOGP) value for all the hybrid compounds was zero from SwissADME, indicating that the compounds have a neutral preference for either n-octanol or water phase and are not considered highly hydrophilic or highly lipophilic. However, the hybrid compounds’ lipophilic nature, predicted by the Consensus Log *P*o/w, was between 0.68 and 3.87 for most of the hybrids, illustrating good lipophilicity. However, hybrids **13**,**14**, **24**, and **25** predicted Log *P* was greater than 5, demonstrating the high lipophilic nature of these triterpenoid-based compounds (Table 12).

## 4. Conclusions

In this study, sixteen ferrocene-based drugs were synthesized and tested against bacterial strains and cancer cells to evaluate their efficacy. The hybrids were successfully synthesized, as confirmed by the FTIR, NMR, and LC-MS. Among the synthesized hybrid, most compounds displayed an antibacterial activity superior to ampicillin against Gram-positive and Gram-negative bacterial strains. Specifically, the keto ferrocene butanoic acid hybrids (both ester and amide-linked) displayed antibacterial activity comparable to parent compound **8** and superior to ampicillin against *E. coli*. These hybrids were two- and three-fold more superior than ampicillin against most bacterial strains.

Furthermore, ester-linked hybrids **10**, **13**, and **24** displayed promising anticancer activity against HeLa and CHO cells. This suggested that the type of the linker could be influential to the anticancer efficacy of the compounds, as the amide-linked hybrids displayed no significant anticancer activity. Hybrids **10** and **24** notably displayed good binding energies with cervical cancer proteins. It was compound **10,** which displayed a selective index of more than 1, and this illustrates that hybrid **10** can be recommended for further studies on cervical cancer. Ultimately, the in silico studies displayed predicted results, which showed desired pharmacokinetics, drug-like properties, and toxicity properties. However, due to the inconsistencies in some of the models, more models must be explored to confirm these findings. In addition, some hybrids displayed moderate to poor water solubility that could compromise their bioavailability.

Among the synthesized compounds, hybrid **10** showed promising efficacy against bacterial strains and cancer cells. Hence, it can be recommended as a potential treatment for cervical cancer and bacterial infections. These compounds are characterized by functionalities suitable for incorporating into nanocarriers to enhance their biological activities further. However, further studies are needed. In addition, since these hybrids display dual biological activities, evaluating them for other biological activities, such as antifungal, antiviral, etc., is paramount.

### 4.1. Experimental Section

#### 4.1.1. Compound **9**

Synthetic route 1 was employed to develop this hybrid previously developed and published by Mbese et al. [38], resulting in a dark-orange greasy product. FTIR (cm^−1^): C-H (2929), C=O (1738), C=O (1624), C=C (1580), C-O (1231), C-Fe (480). ^1^HNMR (CDCl_3_) δ (ppm): 7.06 (1H, J = 7.6 Hz, d), 6.73 (1H, J = 7.7 Hz, d), 6.70 (1H, s), 4.84, 4.54, 4.27 (s, 9H), 3.75 (3H, s), 3.11 (2H, J = 6.6 Hz, t), 2.86 (2H, m), 2.73 (3H, J = 6.6 Hz, t), and 2.23 (d, J = 8.7 Hz, 3H). ^13^CNMR (CDCl_3_) δ (ppm): 202.29, 173.66, 153.80, 148.39, 130.79, 120.92, 118.61, 113.01, 72.30, 69.96, 69.23, 51.87, 34.23, 33.69, 27.72, 24.04, and 15.38. MS (*m*/*z*): Theoretical: 418.1231; Found[M+H]: 419. 1218. % Yield: 71%. RF: 0.73. Eluent (6 mL hexane: 3 mL ethyl acetate).

#### 4.1.2. Compound **10**

Synthetic route 1 was employed to develop this hybrid, resulting in a dark-orange greasy product. FTIR (cm^−1^):C-H (2928), C=O (1737), C=O (1624), C=C (1520), C-O (1228), and C-Fe (480). ^1^HNMR (CDCl_3_) δ (ppm): 7.07 (1H, J = 7.6 Hz, d), 6.73 (1H, J = 7.7 Hz, d), 6.70 (1H, s), 4.84, 4.54, 4.27 (s, 9H), 3.75 (3H, s), 3.13 (2H, J = 6.6 Hz, t), 2.86 (2H, m), 2.75 (3H, J = 6.6 Hz, t), and 2.23 (d, J = 8.7 Hz, 3H). ^13^CNMR (CDCl_3_) δ (ppm): 201.91, 172.06, 152.62, 147.96, 136.58, 126.44, 122.76, 116.01, 72.30, 69.97, 69.23, 34.20, 27.92, 26.71, 23.14, 22.70, and 20.85. MS (*m*/*z*): Theoretical: 418.1231; Found[M+H]: 419.128. % Yield: 68%. RF: 0.71. Eluent (6 mL hexane: 3 mL ethyl acetate).

#### 4.1.3. Compound **11**

Synthetic route 1 was employed to develop this hybrid, resulting in a dark-orange powder product. FTIR (cm^−1^): N-H (3486), C-H (2914), C=O (1739), C=C (1614), C-N (1367), C-O (1217), and C-Fe (467). ^1^HNMR δ (DMSO) (ppm): 5.76 (2H, s), 4.82, 4.57, 4.29 (9H, s), 3.03 (2H, t), 2.51 (2H, t), 2.43 (2H, J = 8.1 Hz, t), 2.15 (2H, J = 7.6 Hz, t), 1.99 (1H, s), 1.92 (2H, s), 1.87 (2H, s), 1.35 (3H, s), and 1.24 (3H, s). ^13^CNMR δ (DMSO) (ppm): 202.20, 174.49, 79.10, 72.36, 70.10, 69.40, 40.63, 34.33, and 28.02. MS (*m*/*z*): Theoretical: 534.1257; Found[M+2H]: 536.1634. % Yield: 76%. RF: 0.66. MP: 110–115 °C. Eluent (7 mL toluene: 5 mL ethyl acetate).

#### 4.1.4. Compound **12**

Synthetic route 1 was employed to develop this hybrid as it was previously developed by Peter et al. [16], resulting in a dark-orange powder product. FTIR (cm^−1^): O-H (3180), C-H (2958), C=O (1739), C=C (1571), C-O (1217), C-O (1122), and C-Fe (463). ^1^HNMR (DMSO) δ (ppm): 7.57 (1H, J = 15.7 Hz, d), 7.11–7.05 (1H, J = 14.3 Hz, d/d), 6.52 (1H, 15.7 Hz d), 5.79 (1H, s), 5.22 (1H, s), 4.76, 4.45, 4.16 (9H, s), 3.82 (s, 3H), 3.12 (t, J = 6.5 Hz, 1H), 2.93 (t, J = 6.5 Hz, 1H), and 2.28 (2H, s). ^13^CNMR (DMSO) δ (ppm): 202.33, 191.42, 182.01, 148.41, 133.72, 132.60, 127.65, 122.59, 116.06, 110.99, 108.58, 79.38, 72.37, 70.09, 69.39, 48.00, 33.78, 31.47, and 24.90. MS (*m*/*z*): Theoretical: 636.1603; Found[M+H]: 637.1476. % Yield: 66%. RF: 0.55. MP: 100–109 °C. Eluent (7 mL toluene: 5 mL ethyl acetate).

#### 4.1.5. Compound **13**

Synthetic route 1 was employed to develop this hybrid, resulting in a dark-orange powder product. FTIR (cm^−1^): O-H (3325), 2971 (C-H), C=O (1739), C=C (1437), C-O (1217), and C-Fe (528). ^1^HNMR (CDCl_3_) δ (ppm): 5.27 (1H, J = 6.5 Hz, t), 4.80, 4.52, 4.26 (9H, s), 3.23 (1H, J = 12.1 Hz, t), 3.03 (1H, J = 13.6 Hz, d), 2.20 (2H, J = 13.4 Hz, t), 1.97 (2H, t), and 1.77–0.70 (aliphatic protons). ^13^CNMR (DMSO) δ (ppm): 202.42, 178.99, 162.73, 149.65, 121.99, 77.32, 72.37, 70.09, 69.45, 55.30, 53.46, 50.01, 47.98, 46.20, 37.08, 36.21, 34.64, 33.80, 33.27, 32.58, 32.18, 31.23, 30.83, 28.68, 27.45, 25.95, 25.79, 24.89, 23.82, 23.39, 23.11, and 16.45, 15.54. MS (*m*/*z*): Theoretical: 724.3790; Found[M+H]: 725.1585. % Yield: 71%. RF: 0.83. MP: 110–125 °C. Eluent (7 mL toluene: 5 mL ethyl acetate).

#### 4.1.6. Compound **14**

Synthetic route 1 was employed to develop this hybrid, resulting in a dark-orange powder product. FTIR (cm^−1^): O-H (3450), C-H (2930), C=O (1737), C=C (1663), C-O (1229), and C-Fe (481). ^1^HNMR (CDCl_3_) δ (ppm): 5.31 (1H, J = 6.5 Hz, t), 4.46, 4.27, 4.17 (9H, s), 3.56 (1H, t), (1H, t), 2.01 (2H, t), 1.79 (2H, t), 1.65-, and 1.61–0.71 (aliphatic protons). ^13^CNMR (DMSO) δ (ppm): 202.42, 178.99, 162.73, 121.99, 107.19, 77.32, 72.37, 70.09, 69.45, 55.30, 53.46, 50.01, 47.98, 46.20, 37.08, 36.21, 34.64, 33.80, 33.27, 32.58, 32.18, 31.23, 30.83, 28.68, 27.45, 25.95, 25.79, 24.89, 23.82, 23.39, 23.11, 16.45, and 15.54. MS (*m*/*z*): Theoretical: 724.3790; Found[M+H]: 725.3789. % Yield: 82%. RF: 0.90. MP: 110–125 °C. Eluent (7 mL toluene: 5 mL ethyl acetate).

#### 4.1.7. Compound **15**

Synthetic route 1 was employed to develop this hybrid, resulting in a dark-orange powder product. FTIR (cm^−1^): O-H (3453), C-H (2923), C=O (1616), C=O (1589), C=C (1499), C-N (1286), and C-Fe (494). ^1^HNMR (CDCl_3_) δ (ppm): 8.70 (1H, s), 7.95 (1H, s), 7.20 (1H, s), 4.74, 4.44, 4.18 (9H, s), 4.07, 4.03, 3.98 (2H, t), 3.36 (2H, t), 2.90 (2H, t), 2.82, 2.57, 2.11, 2.00 (2H, t), 1.63 (2H, s), and 1.19 (3H, t). ^13^CNMR (CDCl_3_) δ (ppm): 202.61, 201.46, 177.09, 169.34, 166.99, 165.46, 162.74, 157.34, 147.66, 108.19, 102.70, 72.31, 69.96, 69.24, 49.30, 46.27, 40.77, 33.80, 25.57, 24.90, 21.32, 14.15, and 8.38. MS (*m*/*z*): Theoretical: 598.1566; Found [M+H]: 599.0463. % Yield: 73%. RF: 0.62. MP: 150–165 °C. Eluent (7 mL toluene: 5 mL ethyl acetate).

#### 4.1.8. Compound **16**

FTIR (cm^−1^): O-H (3482), C-H (2928), C=O (1738), C=O (1624), C=C (1569), C-N (1376), C-F (1242), C-Fe (479). ^1^HNMR (CDCl_3_) δ (ppm): 8.11, 7.28, 6.70, 5.32, 4.84, 4.53, 4.27, 3.47, 3.12, 3.11, 3.09, 2.77, 2.76, 2.74, 2.65, 2.30, 2.23, 2.20, 1.94, 1.70, 1.60, 1.38, 1.35, 1.32, 1.27, 1.17, 1.14, and 1.11. ^13^CNMR (CDCl_3_) δ (ppm): 202.61, 201.46, 177.09, 169.34, 166.99, 165.46, 162.74, 157.34, 147.66, 108.19, 102.70, 72.31, 69.96, 69.24, 49.30, 46.27, 40.77, 33.80, 25.57, 24.90, 21.32, 14.15, and 8.38. MS (*m*/*z*): Theoretical: 586.1566; Found [M+2H]: 586.0463. % Yield: 78%. RF: 0.65. MP: 155–163 °C. Eluent (7 mL toluene: 5 mL ethyl acetate).

#### 4.1.9. Compound **17**

Synthetic route 1 was employed to develop this hybrid, resulting in a dark-orange powder product. FTIR (cm^−1^): N-H (3325), C-H (2927), C=O (1629), C-Fe (483). ^1^HNMR δ (ppm): 5.62 (1H, s), 4.80, 4.55, 4.29 (9H, s), 3.03 (1H, s), 2.83 (3H, s), 1.72 (2H, t), ^13^CNMR δ (ppm): 202.60, 189.48, 171.62, 157.14, 148.85, 125.23, 107.09, 72.21, 70.10, 69.37, 47.98, 40.08, 33.80, and 24.90. MS (*m*/*z*): Theoretical: 462.0739; Found [M+H]: 463.2534. % Yield: 63%. RF: 0.48. MP: 120–135 °C. Eluent (6 mL hexane: 3 mL ethyl acetate).

#### 4.1.10. Compound **18**

Synthetic route 1 was employed to develop this hybrid, resulting in a dark-orange powder product. FTIR (cm^−1^): N-H (3325), C-H (2927), C=O (1657), C-N (1377), C-Fe (478). ^1^HNMR δ (ppm): 6.58 (1H, s), 6.49 (1H, s), 5.76 (1H, s), 4.84, 4.58, 4.28 (9H, s), 3.61 (2H, t), 3.10 (2H, t), 2.98 (1H, s), 2.27 (3H, s), and 1.77 (3H, s). ^13^CNMR δ (ppm): 202.17, 173.13, 171.38, 152.31, 72.38, 70.10, 69.40, 50.44, 47.96, 33.78, 32.53, and 24.90. MS (*m*/*z*): Theoretical: 398.1235; Found [M+H]: 399.8876. % Yield: 68%. RF: 0.66. MP: 125–140 °C. Eluent (6 mL hexane: 3 mL ethyl acetate).

#### 4.1.11. Compound **19**

Synthetic route 1 was employed to develop this hybrid, resulting in a dark-orange powder product. FTIR (cm^−1^): N-H (3323), C-H (2928), C=O (1739), C=O (1623), C=C (1568), C-N (1217), C-Cl (640), and C-Fe (417). ^1^HNMR δ (ppm): 7.48 (1H, s), 7.21 (2H, s), 6.57 (1H, d), 5.93 (1H, d), 4.83, 4.57, 4.28 (9H, s), 2.35 (2H, t), 1.73 (3H, t), 1.15 (2H, m), 1.06, 0.97. ^13^CNMR δ (ppm): 202.27, 166.59, 162.49, 157.14, 135.31, 133.77, 132.99, 132.33, 129.38, 105.86, 72.37, 70.09, 67.71, 47.99, 33.79, 27.74, and 24.90. MS (*m*/*z*): Theoretical: 516.1015; Found [M+H]: 517.1061. % Yield: 82%. RF: 0.78. MP: 170–182 °C. Eluent (6 mL hexane: 3 mL ethyl acetate).

#### 4.1.12. Compound **22**

Synthetic route 2 was employed to develop this hybrid, resulting in a dark-orange oily product. FTIR (cm^−1^): C-H (2928), C=O (1625), C-O (1243), C-O (1087), C-Fe (416). ^13^C NMR (CDCl_3_) (ppm): 162.74, 153.93, 93.54, 90.97, 79.98, 69.53, 68.75, 68.54, 54.88, 50.06, 49.18, 47.18, 36.52, 35.37, 33.76, 32.52, 30.44, 29.56, 27.55, 26.15, 24.87, 22.49, and 20.43. MS (*m*/*z*): Theoretical: 582.1916; Found: 582.3478. % Yield: 67%. RF: 0.85. Eluent (6 mL hexane: 3 mL ethyl acetate).

#### 4.1.13. Compound **23**

Synthetic route 2 was employed to develop this hybrid, resulting in a dark-orange powder product. FTIR (cm^−1^): C-H (2952), C=O (1616), C=C (1554), C=C (1440), C-O (1220), and C-Fe (478). ^1^HNMR (CDCl_3_) δ (ppm): 7.73 (1H, J = 16.0 Hz, d), 7.55 (1H, J = 3.7 Hz, t), 7.42 (1H, J = 08.0 Hz, d), 6.79 (1H, J = 15.3 Hz, d), 6.48 (1H, J = 16.0 Hz, d), 4.30, 4.29, 4.27 (9H, s), and 4.20 (2H, s). ^13^CNMR (CDCl_3_) δ (ppm): 162.56, 157.13, 154.63, 148.39, 144.60, 122.53, 122.00, 106.54, 88.40, 68.51, 68.26, 67.91, and 60.69. MS (*m*/*z*): Theoretical: 346.0656; Found [M+H]: 347.2266. % Yield: 62%. RF: 0.73. MP: 95–102 °C. Eluent (6 mL hexane: 3 mL ethyl acetate).

#### 4.1.14. Compound **24**

Synthetic route 2 was employed to develop this hybrid, resulting in a dark-orange powder product. FTIR (cm^−1^): O-H (3411), C-H (2922), C=O (1604), C=O (1450), C-O (1210), and C-Fe (482). ^1^HNMR(CDCl_3_) δ (ppm): 5.37 (1H, J = 10.8 Hz, t), 4.36, 4.26, 4.19 (9H, s), 3.68 (1H, s), 3.50 (2H, s), 3.10 (1H, t), 1.93–0.80 (aliphatic protons). ^13^CNMR (CDCl_3_) δ (ppm): 176.17, 154.63, 125.42, 88.47, 68.51, 68.26, 67.89, 60.72, and 55.75–14.09. MS (*m*/*z*): Theoretical: 654.3735; Found: 654.3723. % Yield: 84%. RF: 0.68. MP: 105–115 °C. Eluent (6 mL hexane: 3 mL ethyl acetate).

#### 4.1.15. Compound **25**

Synthetic route 2 was employed to develop this hybrid, resulting in a dark-orange powder product. FTIR (cm^−1^): O-H (3411), C-H (2851), C=O (1670), C=C (1450), C-O (1219), C-Fe (480). ^1^HNMR(CDCl_3_) δ (ppm): 5.37 (1H, J = 10.8 Hz, t), 4.36, 4.26, 4.19 (9H, s), 3.68 (1H, s), 3.50 (2H, s), 3.10 (1H, t), and 1.93–0.80 (aliphatic protons). ^13^CNMR (CDCl_3_) δ (ppm): 176.17, 154.63, 125.42, 88.47, 68.51, 68.26, 67.89, 60.72, and 55.75–14.09. MS (*m*/*z*): Theoretical: 654.3735; Found: 654. 3731. % Yield: 68%. RF: 0.70. MP: 105–115 °C. Eluent (7 mL toluene: 5 mL ethyl acetate).

#### 4.1.16. Compound **26**

Synthetic route 2 was employed to develop this hybrid, resulting in a yellow powder product. FTIR (cm^−1^): N-H (3324), O-H (3234), C-H (2926), C=O (1750), C=C (1574), C-N (1236), C-O (1236), and C-Fe (479). ^1^HNMR (CDCl_3_) δ (ppm): 8.02 (1H, s), 7.65 (2H, s), 7.55 (1H, J = 8.6 Hz, d), 7.38 (2H, s), 7.15 (2H, J = 10.1 Hz, d), 6.74 (1H, s), 4.35, 4.25, 4.19 (9H, s), 3.49 (2H, s), 2.97 (2H, s), 2.89 (3H, s), 1.92 (2H, m), 1.35 (2H, t), 1.30, and 1.27. ^13^CNMR δ (CDCl_3_) (ppm): 179.99, 174.36, 170.34, 162.56, 156.90, 153.33, 147.11, 132.48, 128.51, 126.96, 123.96, 120.73, 110.85, 88.40, 68.27, 67.90, 67.44, 60.73, 50.46, 49.09, 36.46, 33.93, and 29.67. MS (*m*/*z*): Theoretical: 652.1845; Found: 652.3303 m/z. % Yield: 59%. RF: 0.48. MP: 160–170 °C. Eluent (7 mL toluene: 5 mL ethyl acetate).

## Figures and Tables

**Table 1:** Advantages and limitations of different types of hybrid linkers [14,17,27].

Type of the Linker	Examples	Advantages	Disadvantages
Cleavable	Esters Amides, Carbamates Phosphates	Stable in the bloodstream. Dual-targets.	Early drug release.
Non-cleavable	Thioether Maleimidocaproyl	More stable in a plasma environment compared to cleavable linkers.	Attached to one targeted site, resulting in compromised efficacy.

**Table 2:** Antibacterial minimum inhibitory concentration (MIC, µg/mL) of the keto ferrocene butanoic acid (**8**), ampicillin, and the hybrids.

Hybrids	Minimum Inhibitory Concentration (MIC, µg/mL)											
	Gram-Positive					Gram-Negative						
	*BS*	*EF*	*SE*	*SA*	*MS*	*EC*	*PV*	*KO*	*PA*	*PM*	*E. coli*	*KP*
**8**	15.625	31.25	250	15.625	15.625	62.5	15.625	125	15.625	15.625	15.625	250
**9**	15.625	62.5	250	125	31.25	31.25	15.625	125	31.25	15.625	15.625	250
**10**	15.625	15.625	250	31.25	15.625	15.625	15.625	31.25	15.625	15.625	15.625	250
**11**	15.625	15.625	250	31.5	62.5	62.5	15.625	125	15.625	15.625	15.625	250
**12**	125	250	250	15.625	62.5	15.625	15.625	125	125	125	15.625	250
**13**	250	125	250	125	125	125	125	125	125	125	15.625	250
**14**	250	250	250	15.625	15.625	15.625	250	62.5	250	250	250	250
**15**	15.625	15.625	250	15.625	15.625	15.625	15.625	15.625	15.625	15.625	15.625	250
**16**	15.625	15.625	15.625	15.625	15.625	15.625	15.625	15.625	15.625	15.625	15.625	125
**17**	15.625	15.625	250	15.625	62.5	15.625	125	31.25	15.625	15.625	15.625	250
**18**	15.625	15.625	250	15.625	31.25	15.625	15.625	125	62.5	15.625	15.625	250
**19**	15.625	15.625	62.5	15.625	15.625	15.625	15.625	15.625	15.625	15.625	15.625	250
**22**	7.8125	7.8125	250	500	250	7.8125	7.8125	7.8125	7.8125	250	250	250
**23**	7.8125	7.8125	250	500	250	7.8125	7.8125	7.8125	7.8125	250	250	250
**24**	7.8125	7.8125	250	7.8125	250	7.8125	7.8125	7.8125	7.8125	31.25	250	250
**25**	7.8125	7.8125	500	125	250	7.8125	7.8125	7.8125	7.8125	125	250	250
**26**	7.8125	7.8125	500	500	250	7.8125	7.8125	7.8125	7.8125	250	250	250
Ampicillin	26	26	26	26	26	26	416	26	64	26	26	26

**Table 3:** The in vitro cytotoxicity activity of compound **8**, POA, and hybrids (**9**–**19** and **22**–**26**) was evaluated against HeLa, CHO, and VERO cells.

Compounds	IC_50_ Values (µg/mL)								
	HeLa Cancer Cells			CHO Cells			Vero Normal Cells		
	24 h	48 h	72 h	24 h	48 h	72 h	24 h	48 h	72 h
**8**	>100	>100	>100	*NT*	*NT*	*NT*	*NT*	*NT*	*NT*
POA	55.78	0.0243	5.9360	*NT*	*NT*	*NT*	*NT*	*NT*	*NT*
**9**	>100	>100	>100	*NT*	*NT*	*NT*	*NT*	*NT*	*NT*
**10**	42.42	44.94	45.37	73.37	50.64	54.61	86.22	83.76	85.70
**11**	>100	>100	>100	*NT*	*NT*	*NT*	*NT*	*NT*	*NT*
**12**	>100	>100	>100	*NT*	*NT*	*NT*	*NT*	*NT*	*NT*
**13**	>100	>100	>100	94.00	68.63	64.63	>100	>100	>100
**14**	>100	>100	>100	>100	>100	>100	>100	>100	>100
**15**	>100	>100	>100	>100	>100	>100	>100	>100	>100
**16**	>100	>100	>100	>100	>100	>100	>100	>100	>100
**17**	>100	>100	>100	*NT*	*NT*	*NT*	*NT*	*NT*	*NT*
**18**	>100	>100	>100	>100	>100	>100	>100	>100	>100
**19**	>100	>100	>100	>100	>100	>100	>100	>100	>100
**22**	>100	>100	>100	*NT*	*NT*	*NT*	*NT*	*NT*	*NT*
**23**	>100	>100	>100	*NT*	*NT*	*NT*	*NT*	*NT*	*NT*
**24**	68.49	75.52	83.97	>100	>100	>100	>100	>100	>100
**25**	>100	>100	>100	>100	>100	>100	>100	>100	>100
**26**	>100	>100	>100	*NT*	*NT*	*NT*	*NT*	*NT*	*NT*

*NT* = not tested.

**Table 4:** Molecular docking scores of hybrids **10** and **24** against selected cervical cancer proteins.

Hybrids	Docking Score (kcal/mol)	Hydrogen Bond	Hydrophobic (Alkyl and pi-Sigma Bonds)
3eqm_10	−8.7	Cys437, Ala438	Ile132, Ile133, Phe148, Ala306, Val370, Val373.
3eqm_24	−8.8	Cys437, Ala438, Gly439	Ile132, Ile133, Phe148, Met303, Ala306, Ala438
4qtb_10	−7.3	Thr223, Asn316	Ala191, Lys224, Pro315
4qtb_24	−9.5	Ser301	Leu261 Lys317
2w3l_10	−7.4	None	Phe63, Tyr67, Phe71 Leu96, Ala108
2w3l_24	−9.3	Glu73, Leu96	Tyr67, Leu96, Ala108
3poz_10	−9.2	Thr854	Leu718, Val726, Ala743, Lys745, Cys755, Met766, Leu777, Leu788, Leu792, Leu844, Phe856
3poz_24	−11.0	None	Lys745, Met766, Leu777, Leu788, Phe856

**Table 5:** The Herg prediction of hybrids **9**–**19** and **22**–**26** were predicted via http://predherg.labmol.com.br/.

Hybrids	Binary Prediction		Confiability%	AD	
	Blocker	Non-Blocker		Inside	Outside
**9**	X		80.31	X	
**10**	X		60.60		X
**11**	X		51.09		X
**12**	X		89.09	X	
**13**	X		71.87		X
**14**	X		54.00	X	
**15**	X		75.02	X	
**16**	X		61.20		X
**17**	X		50.36	X	
**18**	X		78.65		X
**19**		X	80.20	X	
**22**		X	81.80	X	
**23**		X	98.30	X	
**24**		X	87.33		X
**25**		X	86.97	X	
**26**	X		59.39	X	

X = Marker.

**Table 6:** Molecular Weight (g/mol), TPSA (Å^2^), and bioavailability score of the hybrids, **9**–**19** and **22**–**26**, predicted using SwissADME (http://www.swissadme.ch/).

Hybrids	Molecular Weight (g/mol)	Bioavailability Score	TPSA (Å^2^)
**9**	418.1231	0.55	43.37
**10**	418.1231	0.55	43.37
**11**	534.1257	0.55	140.18
**12**	636.1603	0.55	116.20
**13**	724.3790	0.56	80.67
**14**	724.3790	0.56	80.67
**15**	598.1566	0.56	94.99
**16**	586.1566	0.56	94.99
**17**	462.0739	0.55	128.32
**18**	398.1235	0.55	109.14
**19**	516.1015	0.55	97.97
**22**	582.1916	0.55	89.52
**23**	346.0656	0.55	26.30
**24**	654.3735	0.17	45.43
**25**	654.3735	0.17	46.53
**26**	652.1845	0.11	199.54

**Table 7:** The toxicology predictions of hybrid **9**–**19** and **22**–**26** against several parameters using https://tox.charite.de/protox3/.

Hybrids	Classification											
	Organ Toxicity				Toxicity Endpoints							
	Hepatoxicity		Cardiotoxicity		Carcinogenicity		Immunotoxicity		Mutagenicity		Cytotoxicity	
	Prediction	Probability	Prediction	Probability	Prediction	Probability	Prediction	Probability	Prediction	Probability	Prediction	Probability
**9**	Active	0.59	Inactive	0.63	Inactive	0.69	Inactive	0.88	Inactive	0.77	Inactive	0.65
**10**	Active	0.59	Inactive	0.63	Inactive	0.69	Inactive	0.98	Inactive	0.77	Inactive	0.65
**11**	Inactive	0.61	Inactive	0.66	Inactive	0.50	Inactive	0.97	Inactive	0.51	Inactive	0.60
**12**	Active	0.50	Inactive	0.53	Inactive	0.68	Active	0.98	Inactive	0.80	Inactive	0.75
**13**	Inactive	0.70	Active	0.74	Inactive	0.58	Active	0.99	Inactive	0.95	Inactive	0.61
**14**	Inactive	0.70	Active	0.74	Inactive	0.58	Active	0.99	Inactive	0.95	Inactive	0.61
**15**	Inactive	0.70	Inactive	0.77	Inactive	0.58	Active	0.70	Inactive	0.60	Inactive	0.56
**16**	Inactive	0.71	Inactive	0.77	Inactive	0.59	Active	0.78	Inactive	0.64	Inactive	0.55
**17**	Inactive	0.50	Inactive	0.85	Inactive	0.53	Inactive	0.99	Inactive	0.53	Inactive	0.73
**18**	Inactive	0.69	Inactive	0.58	Inactive	0.60	Inactive	0.98	Inactive	0.66	Inactive	0.67
**19**	Inactive	0.57	Inactive	0.77	Inactive	0.61	Inactive	0.98	Inactive	0.66	Inactive	0.74
**22**	Inactive	0.83	Active	0.91	Inactive	0.66	Active	0.98	Inactive	0.69	Inactive	0.63
**23**	Inactive	0.60	Inactive	0.73	Inactive	0.61	Inactive	0.88	Inactive	0.66	Inactive	0.82
**24**	Inactive	0.65	Active	0.82	Inactive	0.57	Active	0.99	Inactive	0.91	Inactive	0.92
**25**	Inactive	0.65	Active	0.82	Inactive	0.57	Active	0.99	Inactive	0.91	Inactive	0.92
**26**	Inactive	0.59	Inactive	0.59	Inactive	0.51	Inactive	0.97	Inactive	0.79	Inactive	0.66

**Table 8:** Administration routes predictions of hybrids **9**–**19** and **22**–**26** using https://www.way2drug.com/Gusar/acutoxpredict.html.

Hybrids	Rat IP				Rat IV				Rat Oral				Rat SC			
	LD_50_ (mg/kg)	Toxicity Class			LD_50_ (mg/kg)	Toxicity Class			LD_50_ (mg/kg)	Toxicity Class			LD_50_ (mg/kg)	Toxicity Class		
		Class	In AD	Out of AD		Class	In AD	Out of AD		Class	In AD	Out of AD		Class	In AD	Out of AD
**9**	150,200	4		X	25,770	3	X		673,100	4	X		381,500	4	X	
**10**	277,900	4	X		23,740	3	X		808,800	4	X		569,600	4	X	
**11**	745,000	5		X	111,700	4	X		1,085,000	4		X	195,500	4		X
**12**	283,600	4	X		53,990	4	X		197,100	3		X	134,800	3	X	
**13**	1,013,000	5	X		11,900	3	X		1,981,000	4	X		57,810	3	X	
**14**	1,032,000	5	X		10,540	3	X		967,200	4	X		47,510	3	X	
**15**	524,600	5		X	51,810	4	X		627,800	4	X		82,050	3		X
**16**	608,900	5		X	47,190	4	X		423,000	3	X		80,010	3		X
**17**	523,000	5		X	178,900	4	X		497,400	3		X	84,390	3		X
**18**	460,300	4		X	81,900	4	X		664,900	4	X		480,000	4	X	
**19**	416,700	4	X		60,120	4	X		983,600	4	X		410,000	4		X
**22**	730,200	5	X		17,410	3	X		1,360,000	4	X		547,100	4	X	
**23**	371,000	4	X		39,230	3	X		1,503,000	4	X		639,700	4	X	
**24**	741,400	5	X		8015	3	X		862,400	4	X		34,730	3	X	
**25**	925,200	5	X		6589	2	X		608,600	4	X		39,920	3	X	
**26**	859,700	5		X	239,800	4	X		3,421,000	5	X		496,200	4	X	

IP, intraperitoneal route of administration; IV, intravenous route of administration; oral, oral route of administration; SC, subcutaneous route of administration.

**Table 9:** Drug likeness of hybrid compounds, **9**–**19** and **22**–**26** predicted usingSwissADME (http://www.swissadme.ch/).

Hybrid	Lipinski	Ghose	Veber	Egan	Muegge
**9**	Yes; 1 violation: MLOGP > 4.15	No; 1 violation: WLOGP > 5.6	Yes	Yes	No; 1 violation: XLOGP3 > 5
**10**	Yes; 1 violation: MLOGP > 4.15	No; 1 violation: WLOGP > 5.6	Yes	Yes	No; 1 violation: XLOGP3 > 5
**11**	Yes; 1 violation: MW > 500	No; 2 violations: MW > 480, MR > 130	No; 2 violations: Rotors > 10, TPSA > 140	No; 1 violation: TPSA > 131.6	Yes
**12**	Yes; 1 violation: MW > 500	No; 4 violations: MW > 480, WLOGP > 5.6, MR > 130, #atoms > 70	No; 1 violation: Rotors > 10	No; 1 violation: WLOGP > 5.88	No; 3 violations: MW > 600, XLOGP3 > 5, Rotors > 15
**13**	No; 2 violations: MW > 500, MLOGP > 4.15	No; 4 violations: MW > 480, WLOGP > 5.6, MR > 130, #atoms > 70	Yes	No; 1 violation: WLOGP > 5.88	No; 2 violations: MW > 600, XLOGP3 > 5
**14**	No; 2 violations: MW > 500, MLOGP > 4.15	No; 4 violations: MW > 480, WLOGP > 5.6, MR > 130, #atoms > 70	Yes	No; 1 violation: WLOGP > 5.88	No; 2 violations: MW > 600, XLOGP3 > 5
**15**	Yes; 1 violation: MW > 500	No; 3 violations: MW > 480, MR > 130, #atoms > 70	Yes	Yes	No; 1 violation: XLOGP3 > 5
**16**	Yes; 1 violation: MW > 500	No; 3 violations: MW > 480, MR > 130, #atoms > 70	Yes	Yes	No; 1 violation: XLOGP3 > 5
**17**	Yes; 0 violation	Yes	Yes	Yes	Yes
**18**	Yes; 0 violation	Yes	No; 1 violation: Rotors > 10	Yes	Yes
**19**	Yes; 1 violation: MW > 500	No; 2 violations: MW > 480, MR > 130	Yes	Yes	Yes
**22**	Yes; 1 violation: MW > 500	No; 3 violations: MW > 480, MR > 130, #atoms > 70	Yes	Yes	No; 1 violation: XLOGP3 > 5
**23**	Yes; 0 violation	Yes	Yes	Yes	Yes
**24**	No; 2 violations: MW > 500, MLOGP > 4.15	No; 4 violations: MW > 480, WLOGP > 5.6, MR > 130, #atoms > 70	Yes	No; 1 violation: WLOGP > 5.88	No; 2 violations: MW > 600, XLOGP3 > 5
**25**	No; 2 violations: MW > 500, MLOGP > 4.15	No; 4 violations: MW > 480, WLOGP > 5.6, MR > 130, #atoms > 70	Yes	No; 1 violation: WLOGP > 5.88	No; 2 violations: MW > 600, XLOGP3 > 5
**26**	No; 2 violations: MW > 500, NorO > 10	No; 3 violations: MW > 480, MR > 130, #atoms > 70	No; 2 violations: Rotors > 10, TPSA > 140	No; 1 violation: TPSA > 131.6	No; 2 violations: MW > 600, TPSA > 150

**Table 10:** The predicted pharmacokinetics of hybrid **9**–**19** and **22**–**26** using SwissADME (http://www.swissadme.ch/).

Hybrids	GI Absorption	BBB Permeant	P-gp Substrate	CYP1A2 Inhibitor	CYP2C19 Inhibitor	CYP2C9 Inhibitor	CYP2D6 Inhibitor	CYP3A4 Inhibitor	Log *K*_p_ (cm/s)
**9**	High	Yes	Yes	No	Yes	Yes	No	Yes	−5.07
**10**	High	Yes	Yes	No	Yes	Yes	No	Yes	−5.07
**11**	Low	No	Yes	No	Yes	Yes	No	No	−6.67
**12**	Low	No	Yes	No	No	Yes	No	No	−6.46
**13**	Low	No	Yes	No	No	No	No	No	−3.57
**14**	Low	No	Yes	No	No	No	No	No	−3.47
**15**	High	No	Yes	No	No	Yes	No	Yes	−6.35
**16**	High	No	Yes	No	No	Yes	No	Yes	−6.31
**17**	High	No	No	No	No	No	No	No	−7.72
**18**	High	No	No	No	No	No	No	No	−7.43
**19**	High	No	Yes	Yes	No	Yes	No	Yes	−6.19
**22**	High	No	Yes	No	No	No	No	No	−6.25
**23**	High	Yes	No	No	Yes	Yes	No	No	−5.27
**24**	Low	No	Yes	No	No	No	No	No	−3.26
**25**	Low	No	Yes	No	No	No	No	No	−3.17
**26**	Low	No	No	No	No	No	No	Yes	−8.79

**Table 11:** The water solubility of the hybrid compounds **9**–**19** and **22**–**26** predicted using SwissADME (http://www.swissadme.ch/).

**Hybrids**	**Water Solubility**								
	**Log S (ESOL)**	**Solubility (mg/mL; mol/L)**	**Class**	**Log S (Ali)**	**Solubility (mg/mL; mol/L)**	**Class**	**Log S (SILICOS-IT)**	**Solubility (mg/mL; mol/L)**	**Class**
**9**	−5.35	1.87 × 10^−3^; 4.47 × 10^−6^	Moderately soluble	−5.98	4.35 × 10^−4^; 1.04 × 10^−6^	Moderately soluble	−4.82	6.40 × 10^−3^; 1.53 × 10^−5^	Moderately soluble
**10**	−5.35	1.87 × 10^−3^; 4.47 × 10^−6^	Moderately soluble	−5.98	4.35 × 10^−4^; 1.04 × 10^−6^	Moderately soluble	−4.82	6.40 × 10^−3^; 1.53 × 10^−5^	Moderately soluble
**11**	−5.16	3.71 × 10^−3^; 6.95 × 10^−6^	Moderately soluble	−6.72	1.02 × 10^−4^; 1.91 × 10^−7^	Poorly soluble	−4.58	1.39 × 10^−2^; 2.60 × 10^−5^	Moderately soluble
**12**	−6.19	4.10 × 10^−4^; 6.42 × 10^−7^	Poorly soluble	−7.45	2.27 × 10^−5^; 3.55 × 10^−8^	Poorly soluble	−6.74	1.15 × 10^−4^; 1.81 × 10^−7^	Poorly soluble
**13**	−10.08	5.98 × 10^−8^; 8.25 × 10^−11^	Insoluble	−11.69	1.46 × 10^−9^; 2.02 × 10^−12^	Insoluble	−7.20	4.58 × 10^−5^; 6.32 × 10^−8^	Poorly soluble
**14**	−10.17	4.88 × 10^−8^; 6.73 × 10^−11^	Insoluble	−11.84	1.05 × 10^−9^; 1.44 × 10^−12^	Insoluble	−7.65	1.63 × 10^−5^; 2.24 × 10^−8^	Poorly soluble
**15**	−6.19	3.84 × 10^−4^; 6.42 × 10^−7^	Poorly soluble	−6.81	9.33 × 10^−5^; 1.56 × 10^−7^	Poorly soluble	−4.21	3.68 × 10^−2^; 6.15 × 10^−5^	Moderately soluble
**16**	−6.10	4.70 × 10^−4^; 8.02 × 10^−7^	Poorly soluble	−6.77	1.01 × 10^−4^; 1.72 × 10^−7^	Poorly soluble	−4.42	2.23 × 10^−2^; 3.80 × 10^−5^	Moderately soluble
**17**	−3.57	1.25 × 10^−1^; 2.71 × 10^−4^	Soluble	−4.29	2.37 × 10^−2^; 5.13 × 10^−5^	Moderately soluble	−1.86	6.35 × 10^+0^; 1.37× 10^−2^	Soluble
**18**	−2.72	7.51 × 10^−1^; 1.89 × 10^−3^	Soluble	−3.73	7.37 × 10^−2^; 1.85 × 10^−4^	Moderate soluble	−1.77	6.79 × 10^+0^; 1.71 × 10^−2^	Soluble
**19**	−5.54	1.48 × 10^−3^; 2.86 × 10^−6^	Moderately soluble	−6.38	2.14 × 10^−4^; 4.15 × 10^−7^	Poorly soluble	−6.63	1.20 × 10^−4^; 2.32 × 10^−7^	Poorly soluble
**22**	−5.99	5.94 × 10^−4^; 1.02 × 10^−6^	Moderately soluble	−6.70	1.16 × 10^−4^; 1.98 × 10^−7^	Poorly soluble	−3.09	4.78 × 10^−1^; 8.20 × 10^−4^	Soluble
**23**	−4.51	1.07 × 10^−2^; 3.10 × 10^−5^	Moderately soluble	−4.70	6.90 × 10^−3^; 1.99 × 10^−5^	Moderately soluble	−3.40	1.38 × 10^−1^; 4.00 × 10^−4^	Soluble
**24**	−9.74	1.19 × 10^−7^; 1.82 × 10^−10^	Poorly soluble	−10.80	7.03 × 10^−8^; 1.58 × 10^−11^	Insoluble	−7.00	6.55 × 10^−5^; 1.00 × 10^−7^	Poorly soluble
**25**	−9.83	9.72 × 10^−8^; 1.48 × 10^−10^	Poorly soluble	−10.95	7.40 × 10^−9^; 1.13 × 10^−11^	Insoluble	−7.45	2.32 × 10^−5^; 3.55 × 10^−8^	Poorly soluble
**26**	−4.48	2.15 × 10^−2^; 3.30 × 10^−5^	Moderately soluble	−5.92	7.83 × 10^−4^; 1.20 × 10^−6^	Moderately soluble	−5.32	3.14 × 10^−3^; 4.82 × 10^−6^	Moderately soluble

**Table 12:** The lipophilicity prediction of hybrids **9**–**26** predicted using SwissADME (http://www.swissadme.ch/).

Hybrid	Log *P*o/w (iLOGP)	Log *P*o/w (XLOGP3)	Log *P*o/w (WLOGP)	Log *P*o/w (MLOGP)	Log *P*o/w (SILICOS-IT)	Consensus Log *P*o/w
**9**	0.00	5.32	5.65	4.30	3.81	3.82
**10**	0.00	5.32	5.65	4.30	3.81	3.82
**11**	0.00	4.07	3.19	1.46	2.81	2.31
**12**	0.00	5.26	6.01	2.73	5.24	3.85
**13**	0.00	10.07	10.27	6.91	6.37	6.73
**14**	0.00	10.21	10.42	6.91	6.77	6.86
**15**	0.00	5.07	4.40	3.14	2.72	3.07
**16**	0.00	5.03	4.46	2.95	2.71	3.03
**17**	0.00	1.97	0.71	2.11	−1.40	0.68
**18**	0.00	1.82	1.75	1.52	−0.76	0.87
**19**	0.00	4.60	5.33	3.15	3.14	3.24
**22**	0.00	5.08	5.34	3.82	1.93	3.23
**23**	0.00	4.43	4.42	4.05	2.41	3.06
**24**	0.00	9.90	9.93	7.44	6.02	6.64
**25**	0.00	10.04	9.97	7.44	6.42	6.77
**26**	0.00	2.10	2.87	1.25	−0.12	1.22

## Data Availability

Data is contained within the article and the Supplementary Material.

## Supplementary Materials

The following supporting information can be downloaded at: https://www.mdpi.com/article/10.3390/pharmaceutics17060722/s1.
