# Supplementary material for: Ferrocene-Based Hybrid Drugs as Potential Anticancer and Antibacterial Therapeutic Agents for Incorporation into Nanocarriers: In Silico, In Vitro, Molecular Docking Evaluations
Source: Pharmaceutics. 2025 May 30;17(6):722. doi: 10.3390/pharmaceutics17060722 (PMC12195905; doi:10.3390/pharmaceutics17060722)
Supplement: Supplementary file 1 [file pharmaceutics-17-00722-s001.zip › pharmaceutics-3626218-supplementary.pdf]

**Keto ferrocenyl butanoic acid (8)**

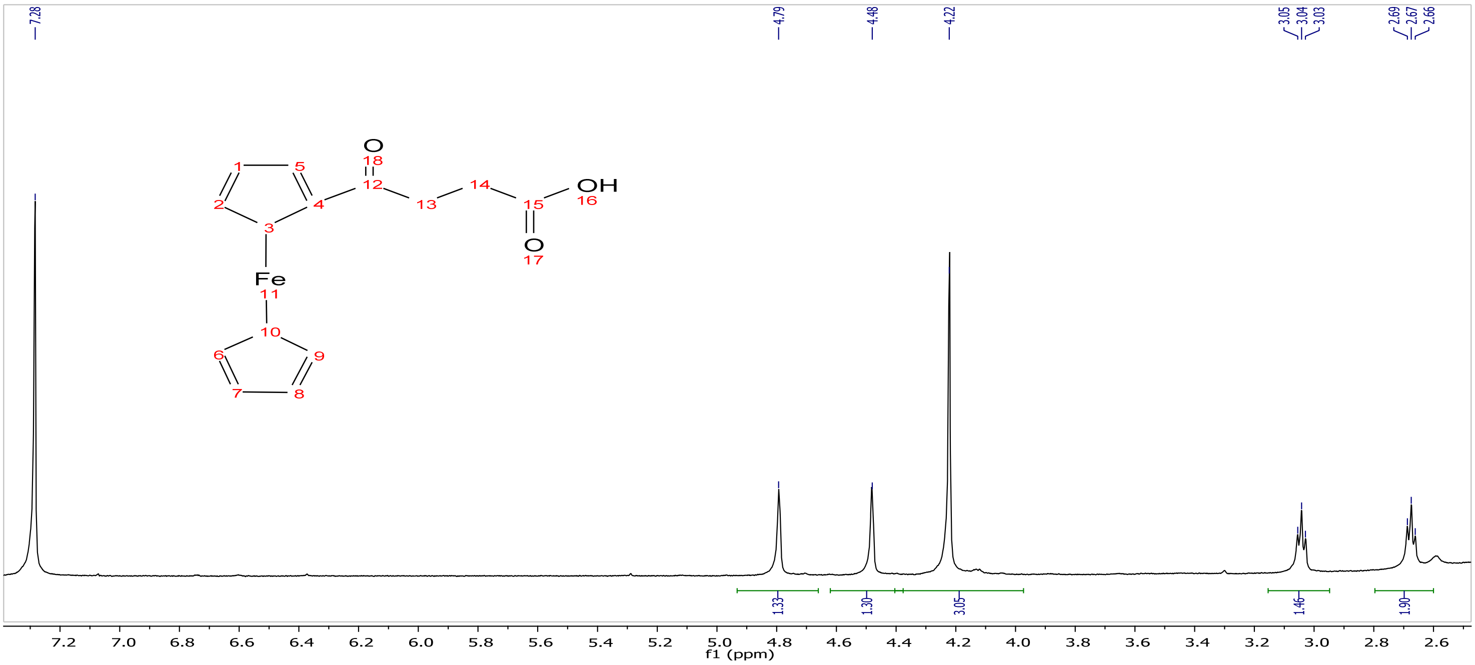


$^{1}$HNMR of keto-ferrocenyl butanoic acid (**8**).

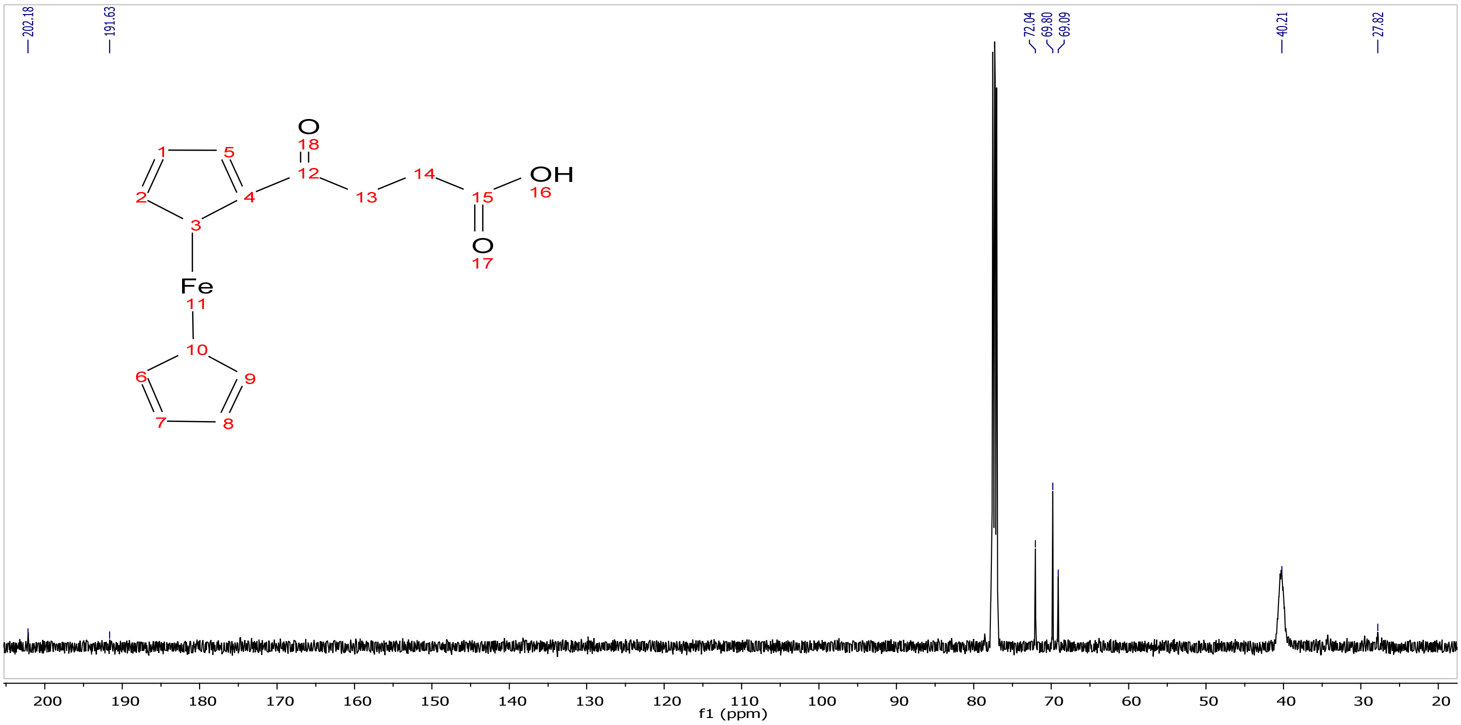


$^{13}$CNMR of keto-ferrocenyl butanoic acid (**8**).

**Methyl-Ferrocenol (21)**

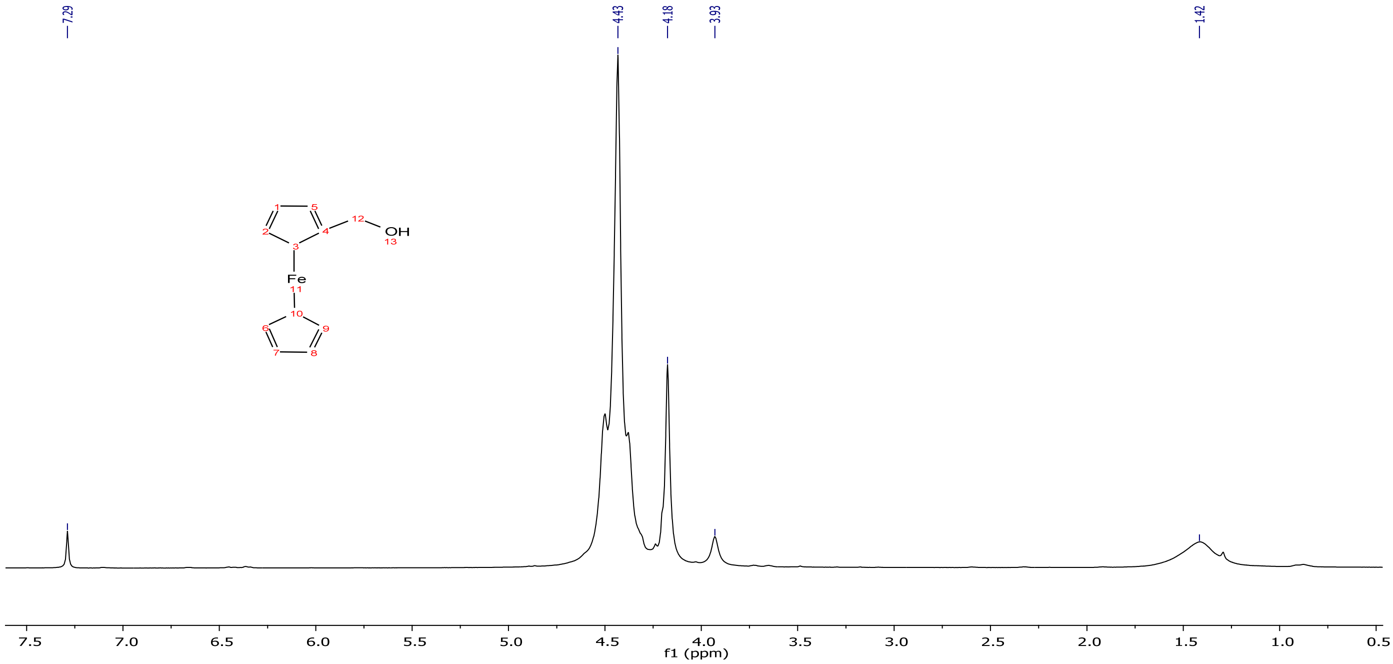


$^{1}$HNMR of methyl-ferrocenol (**21**)

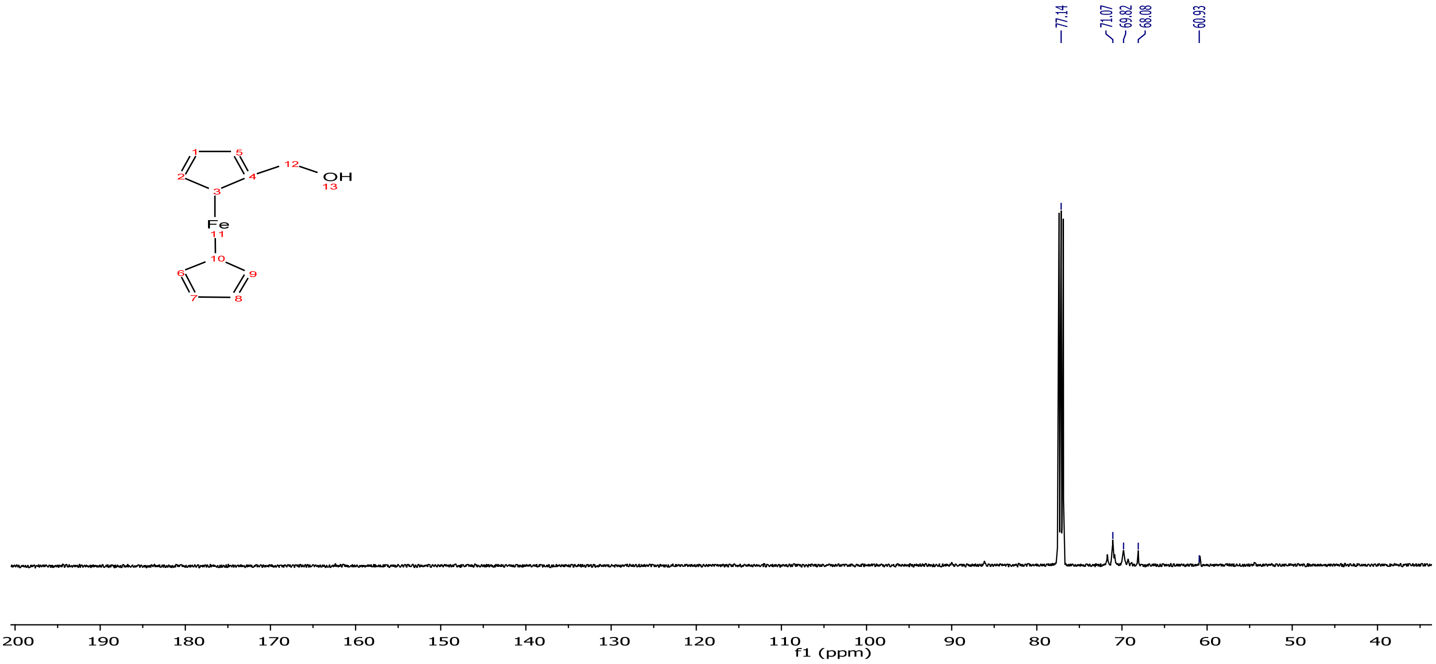


$^{13}$CNMR of methyl-ferrocenol (**21**)

## Carvacrol-keto-ferrocenyl butanoate (9)

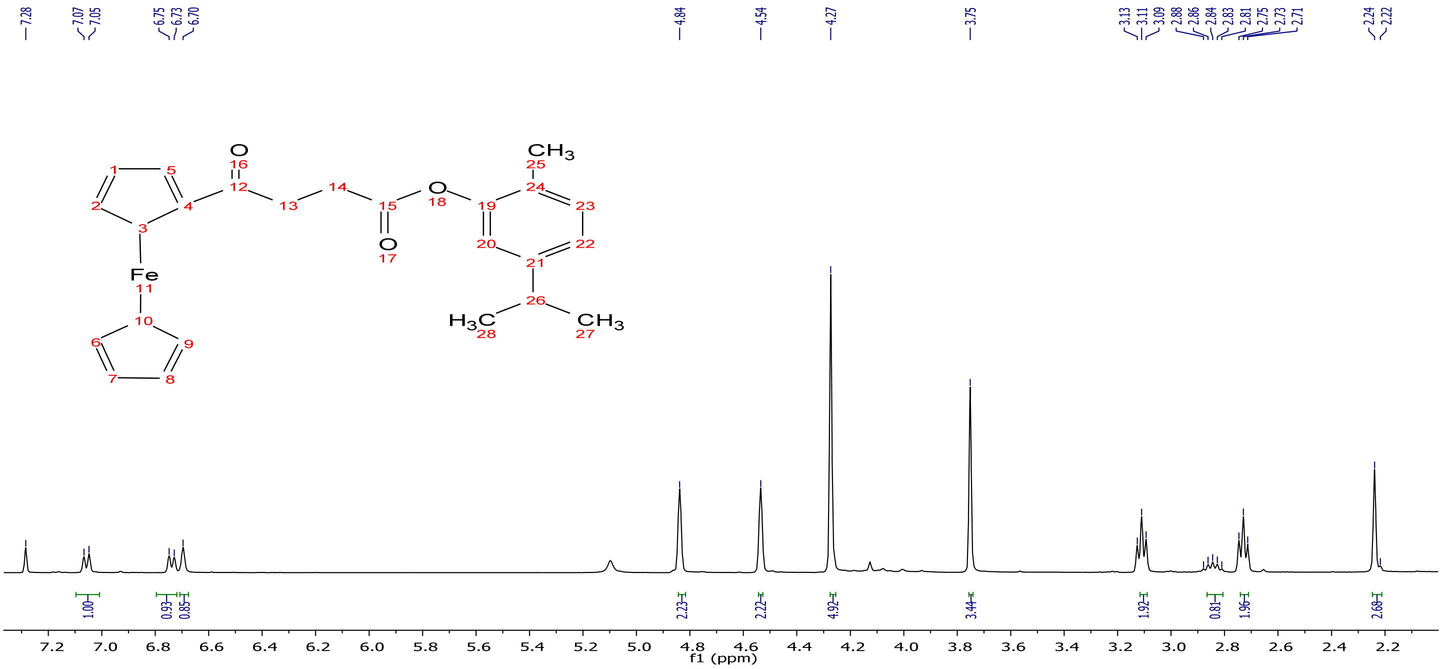


$^{1}$HNMR of carvacrol-keto-ferrocenyl butanoate (**9**).

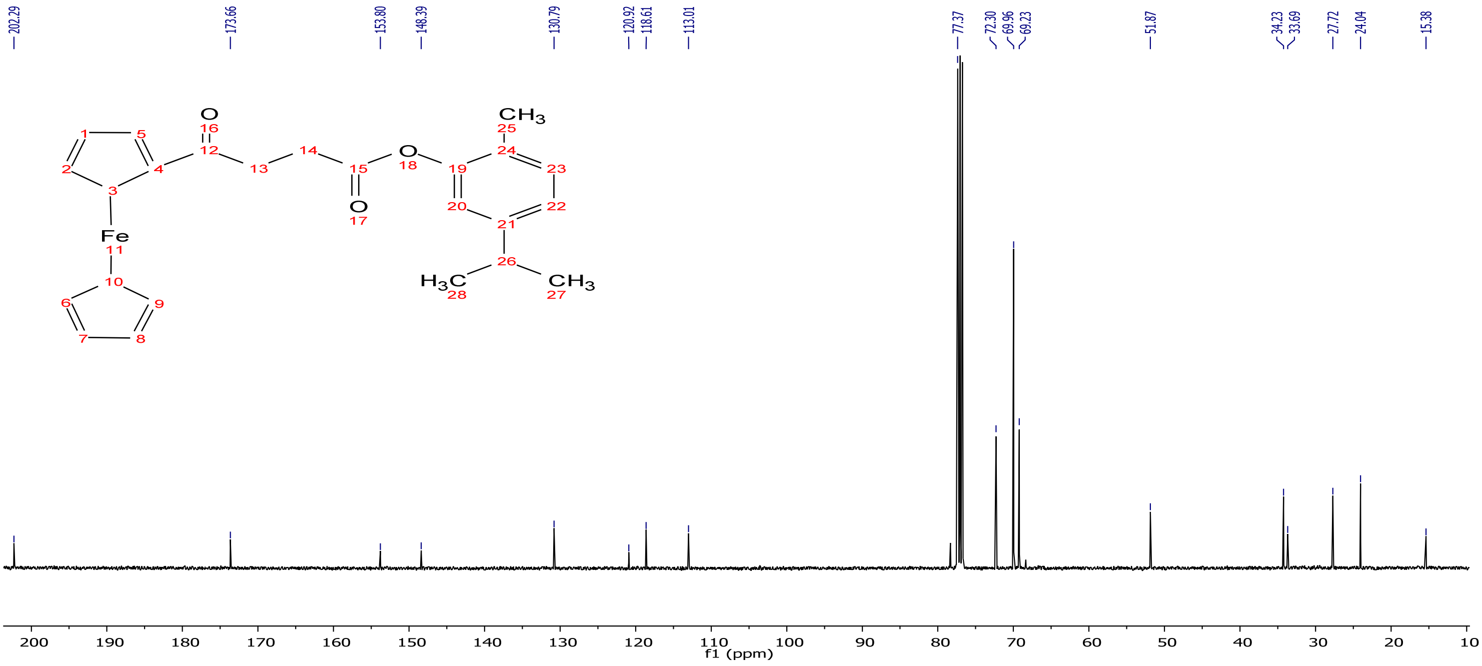


$^{13}$CNMR of carva-keto-ferrocenyl butanoate (**9**).

## Thymol-keto-ferrocenyl butanoate (10)

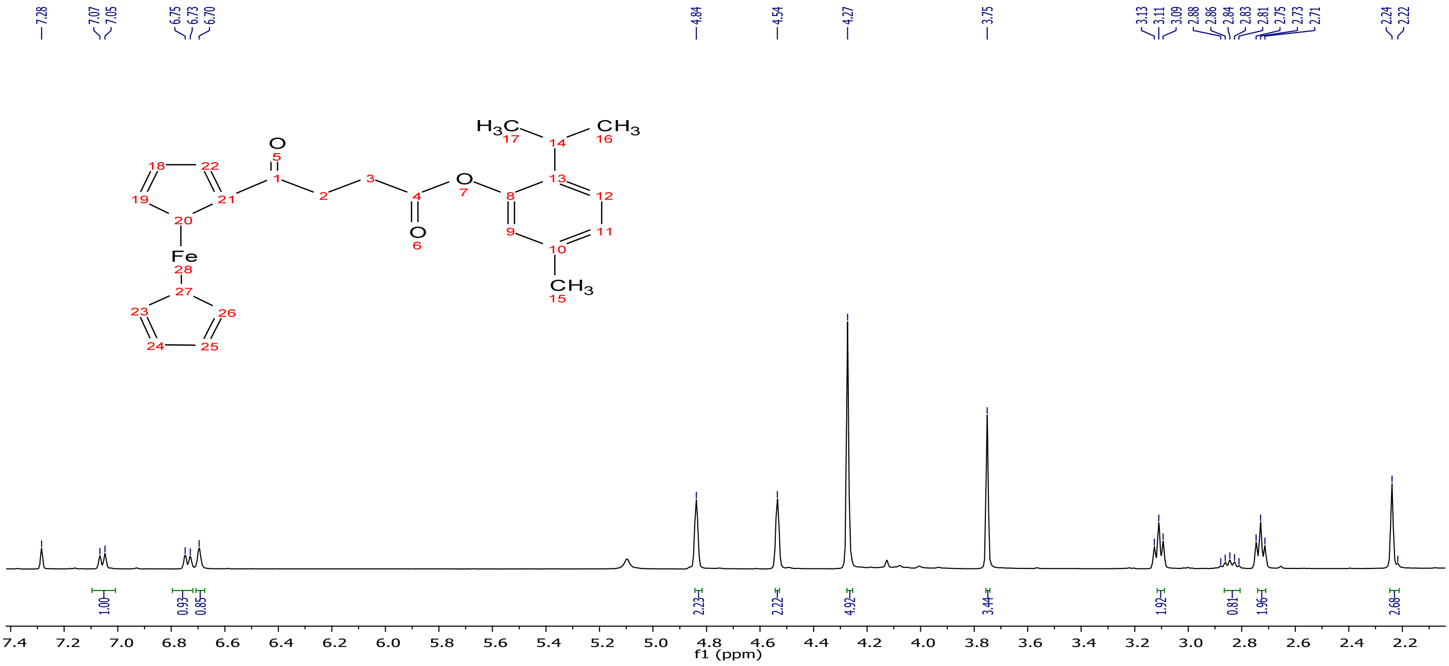


$^{1}$HNMR of thymol keto-ferrocenyl butanoate (**10**).

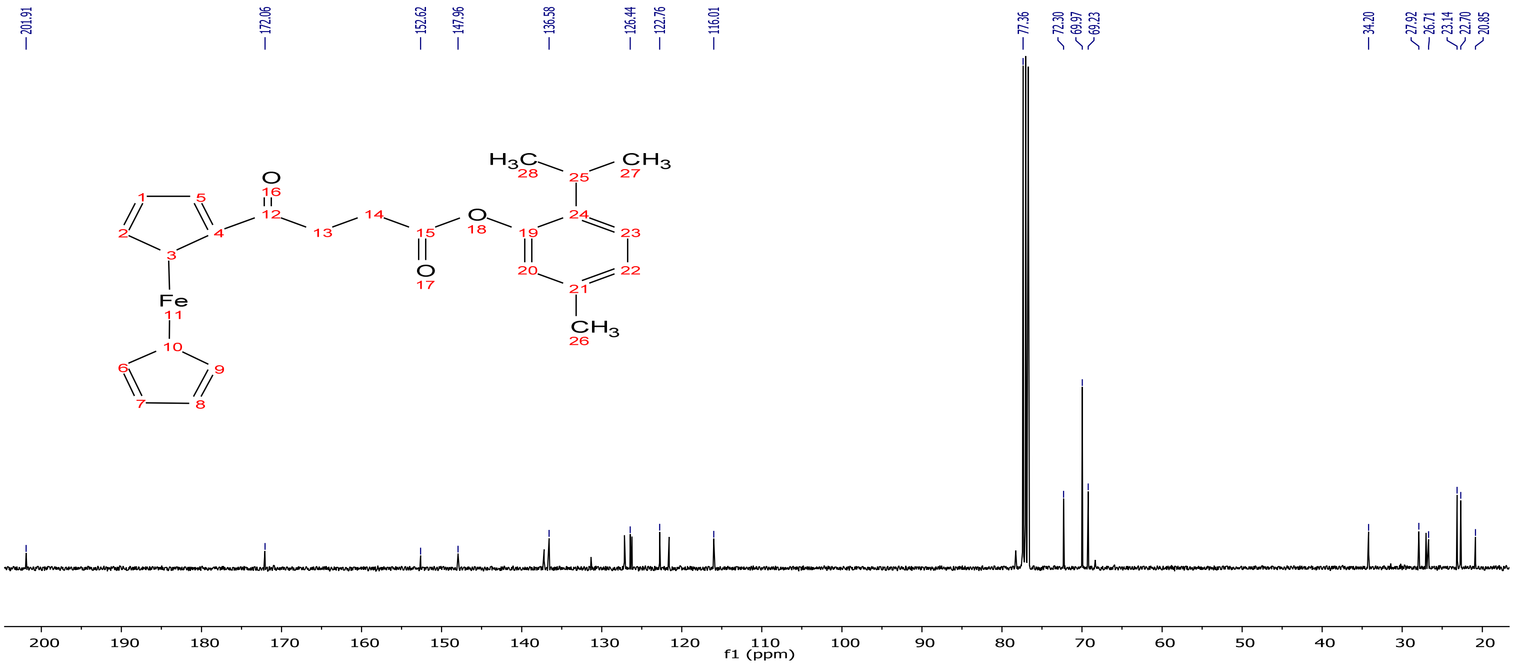


$^{13}$CNMR of thymol keto-ferrocenyl butanoate (**10**).

## Thiamine-keto-ferrocenyl-butanoate (11)

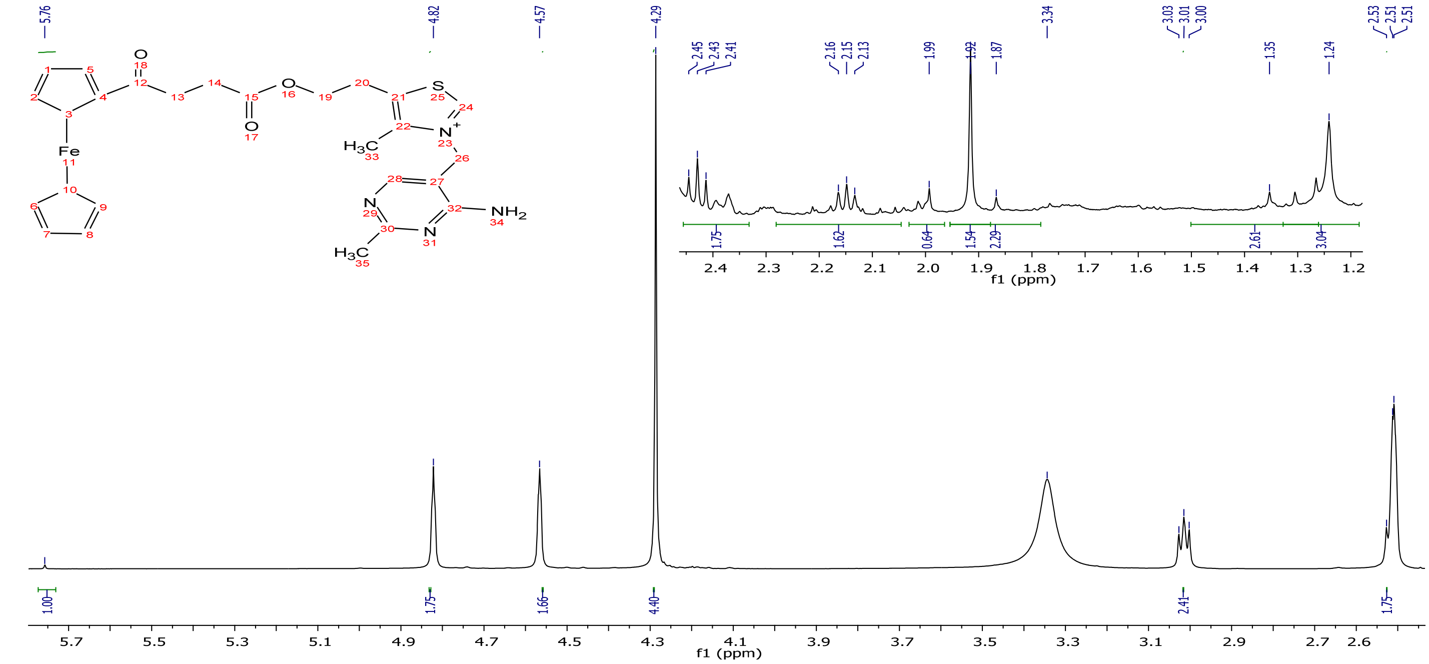


$^{1}$HNMR of thiamine-keto-ferrocenyl butanoate (**11**).

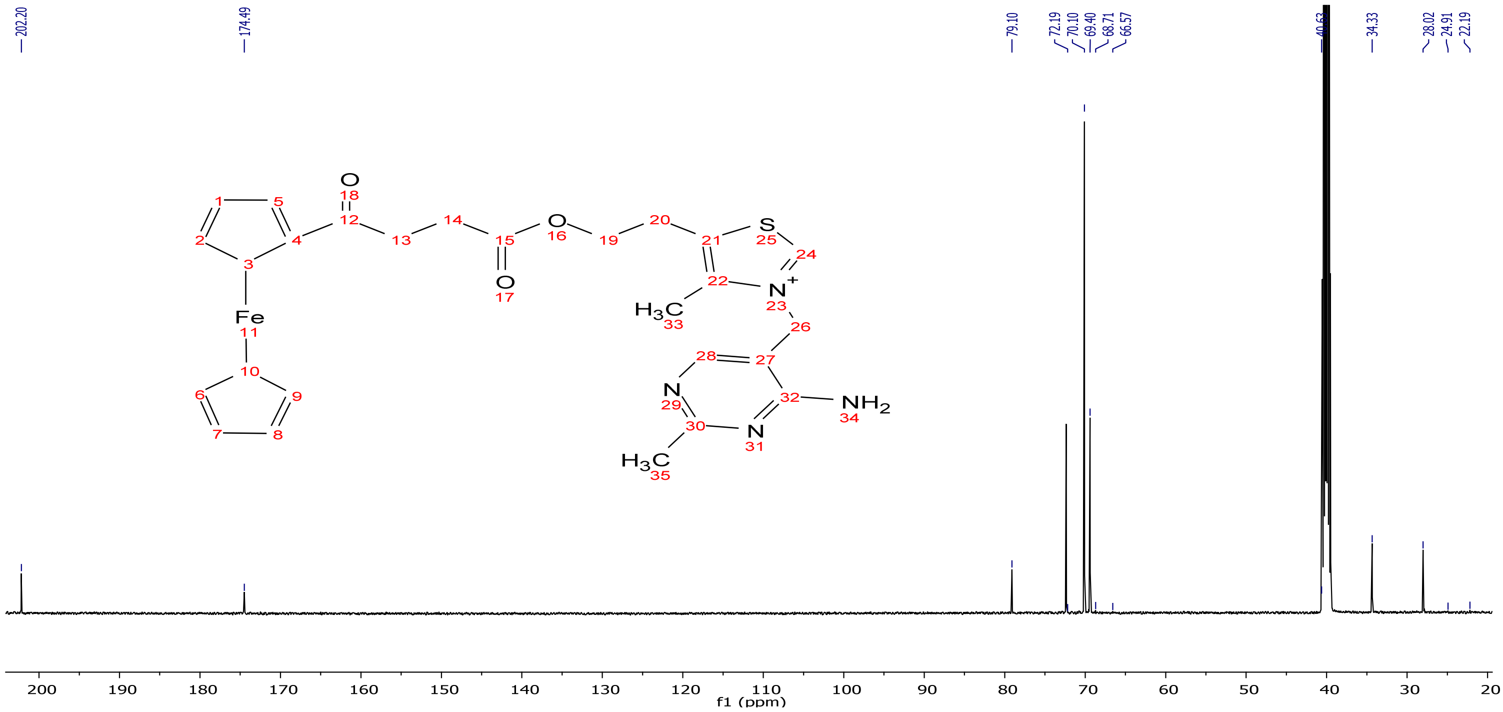


$^{13}$CNMR of thiamine-keto-ferrocenyl butanoate (**11**)

## Curcumin-keto-ferrocenyl butanoate (12)

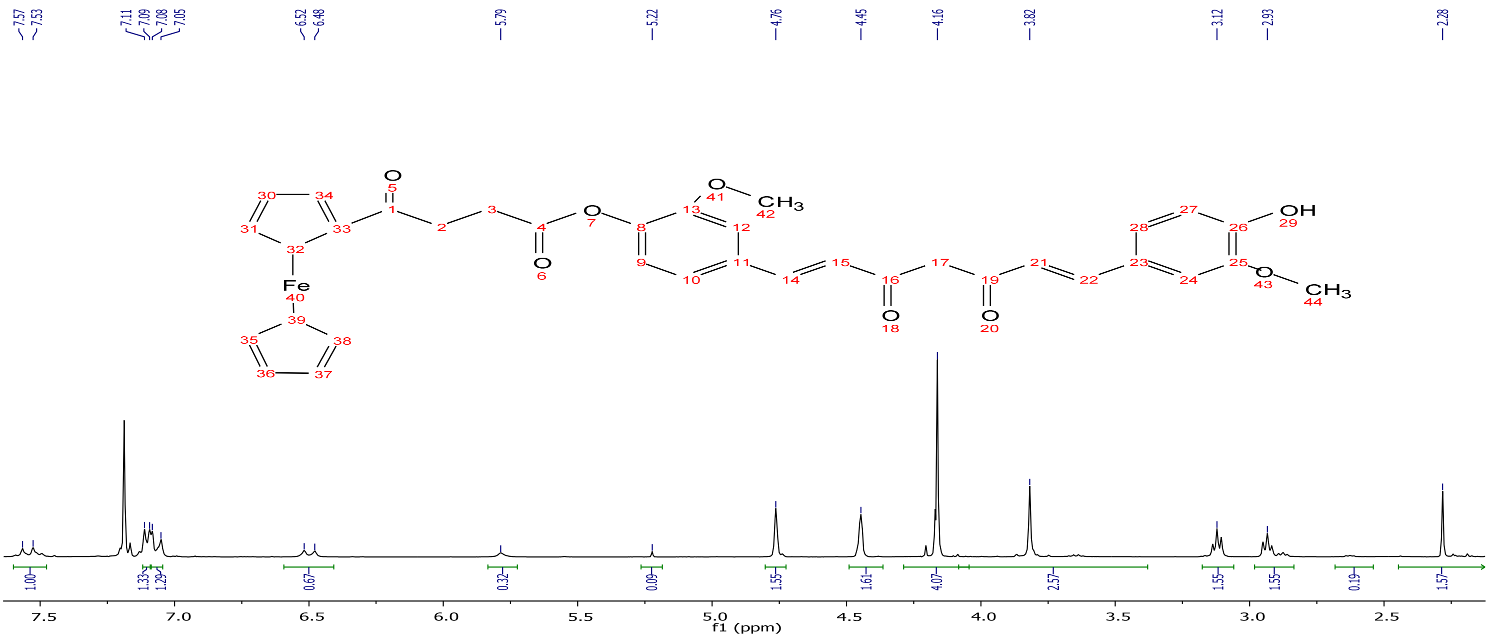

$^{1}$HNMR of curcumin-keto-ferrocenyl butanoate (**12**).

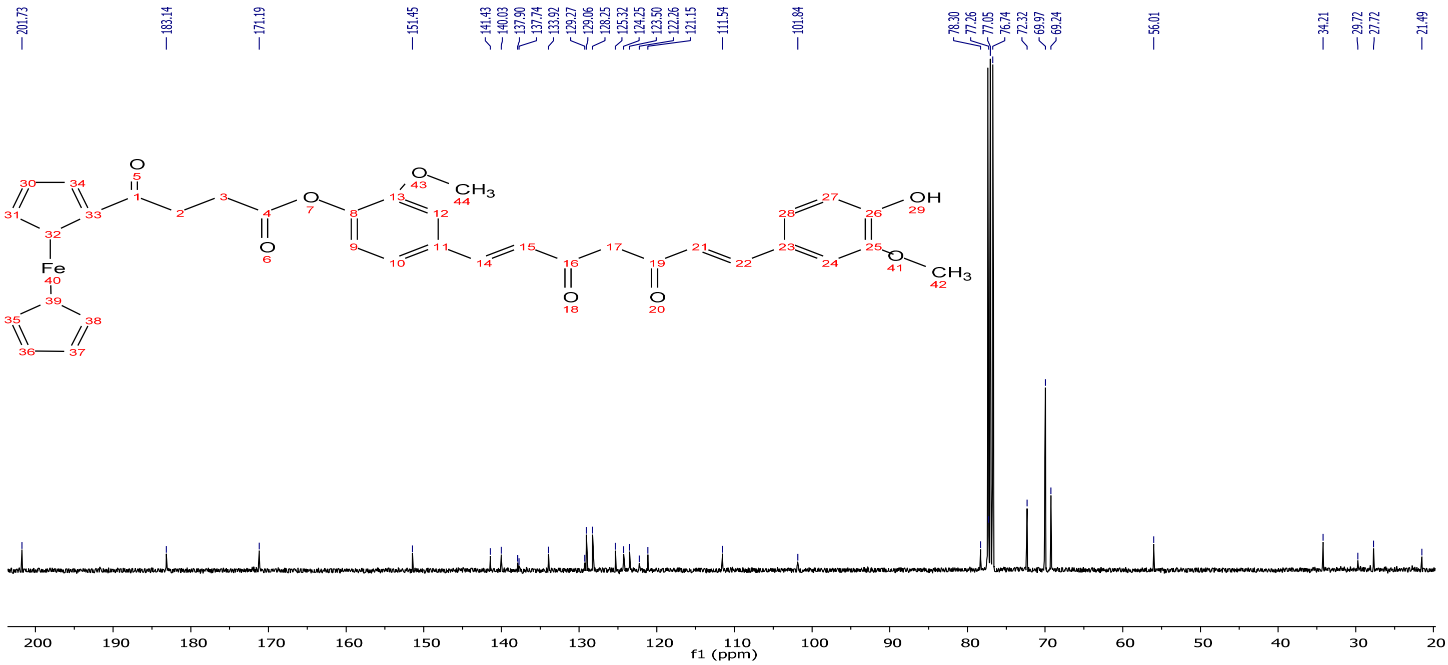

$^{13}$CNMR of curcumin-keto-ferrocenyl butanoate (**12**).

## Ursolic keto-ferrocenyl butanoate (13)

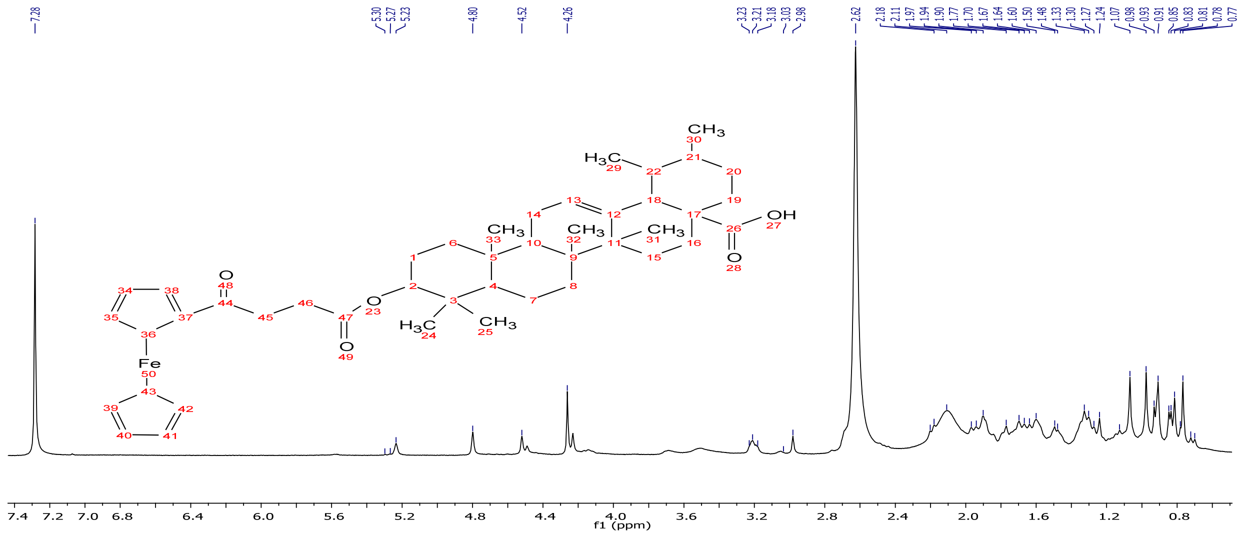


¹HNMR of ursolic keto-ferrocenyl butanoate (**13**).

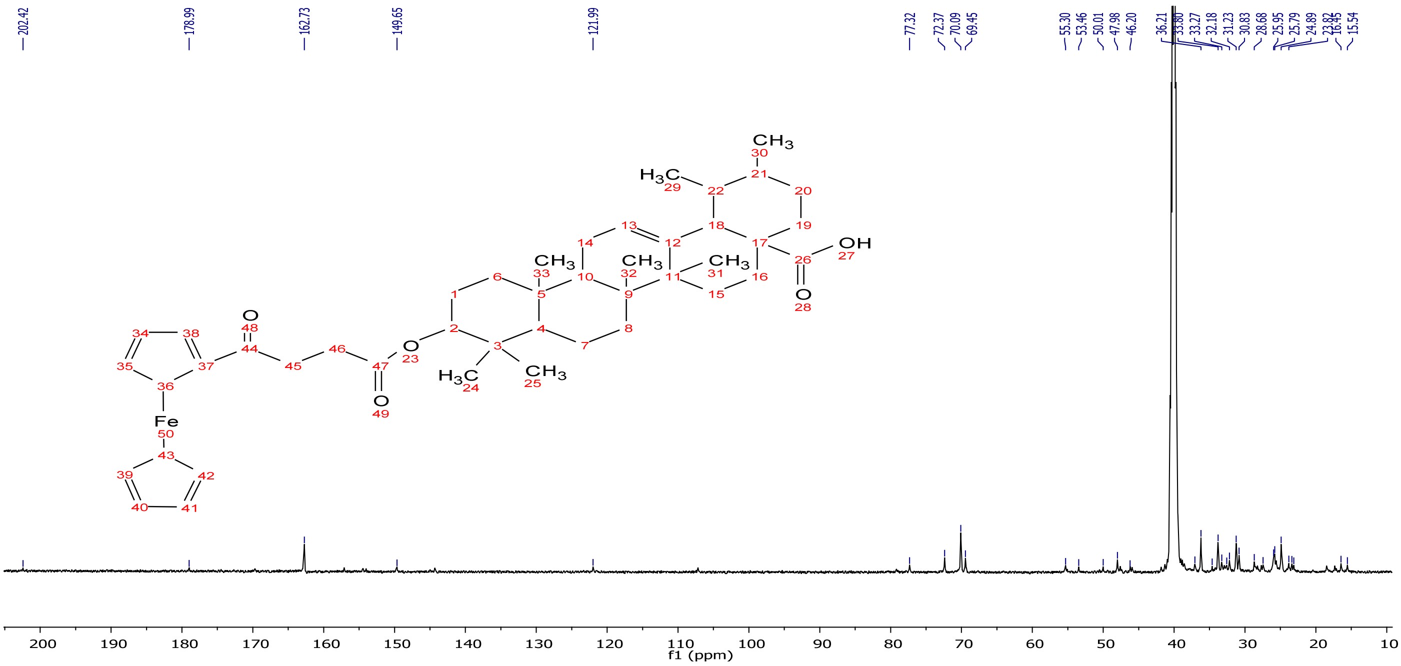


¹³CNMR of ursolic keto-ferrocenyl butanoate (**13**)

## Oleanolic keto-ferrocenyl butanoate (14)

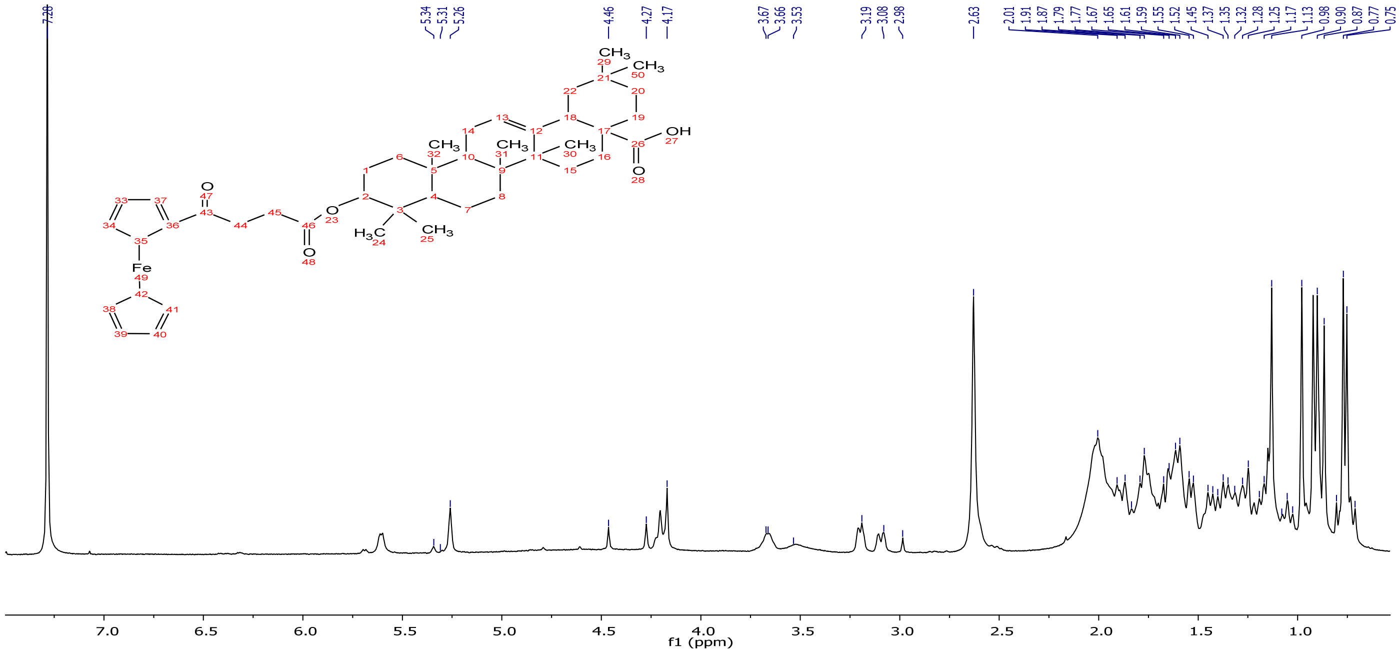


$^{1}$HNMR of oleanolic keto-ferrocenyl butanoate (**14**).

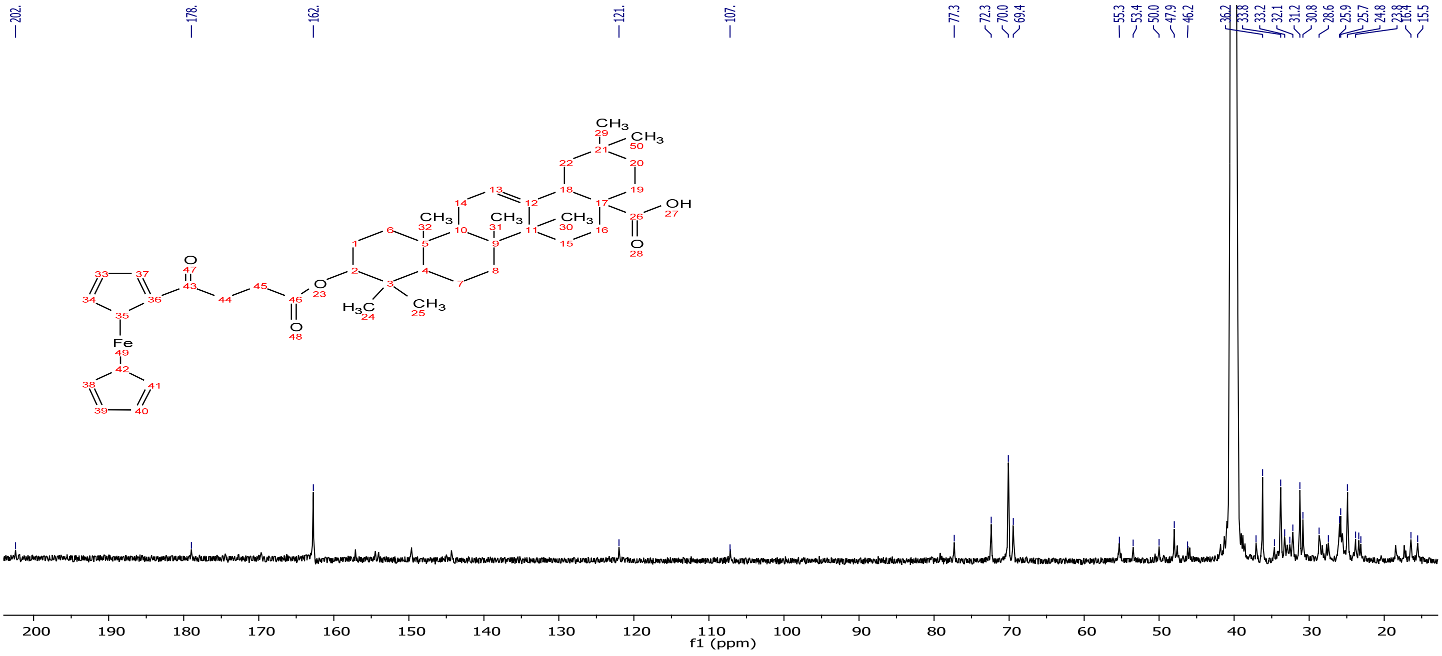


$^{13}$CNMR of oleanolic keto-ferrocenyl butanoate (**14**).

## Ciprofloxacin-keto-ferrocenamide (15)

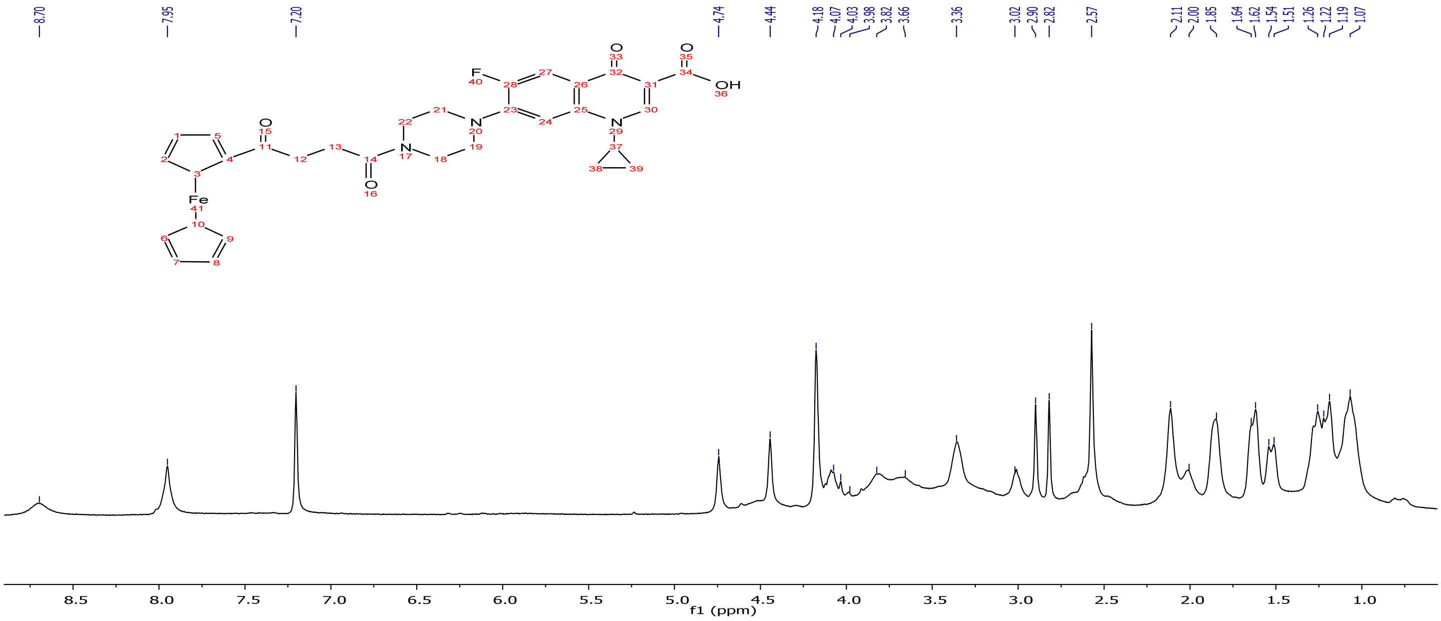

$^{1}$HNMR of ciprofloxacin-keto-ferrocenamide (**15**).

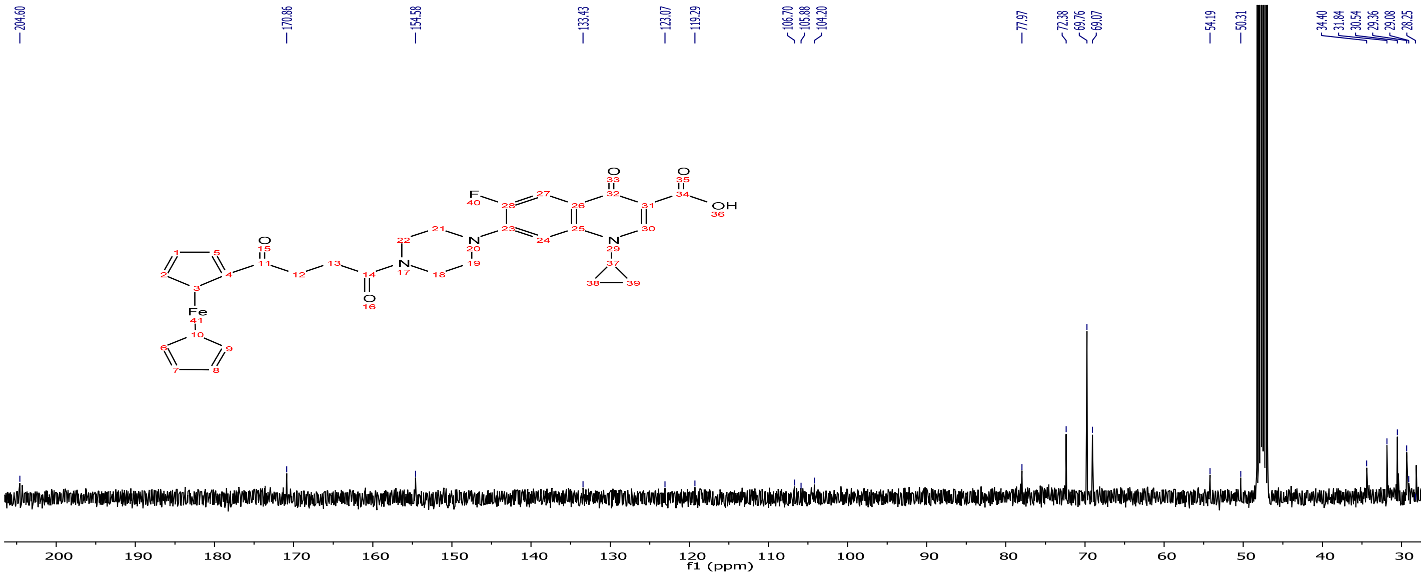

$^{13}$CNMR of ciprofloxacin-keto-ferrocenamide (**15**).

**Norfloxacin-keto-ferrocenamide (16)**

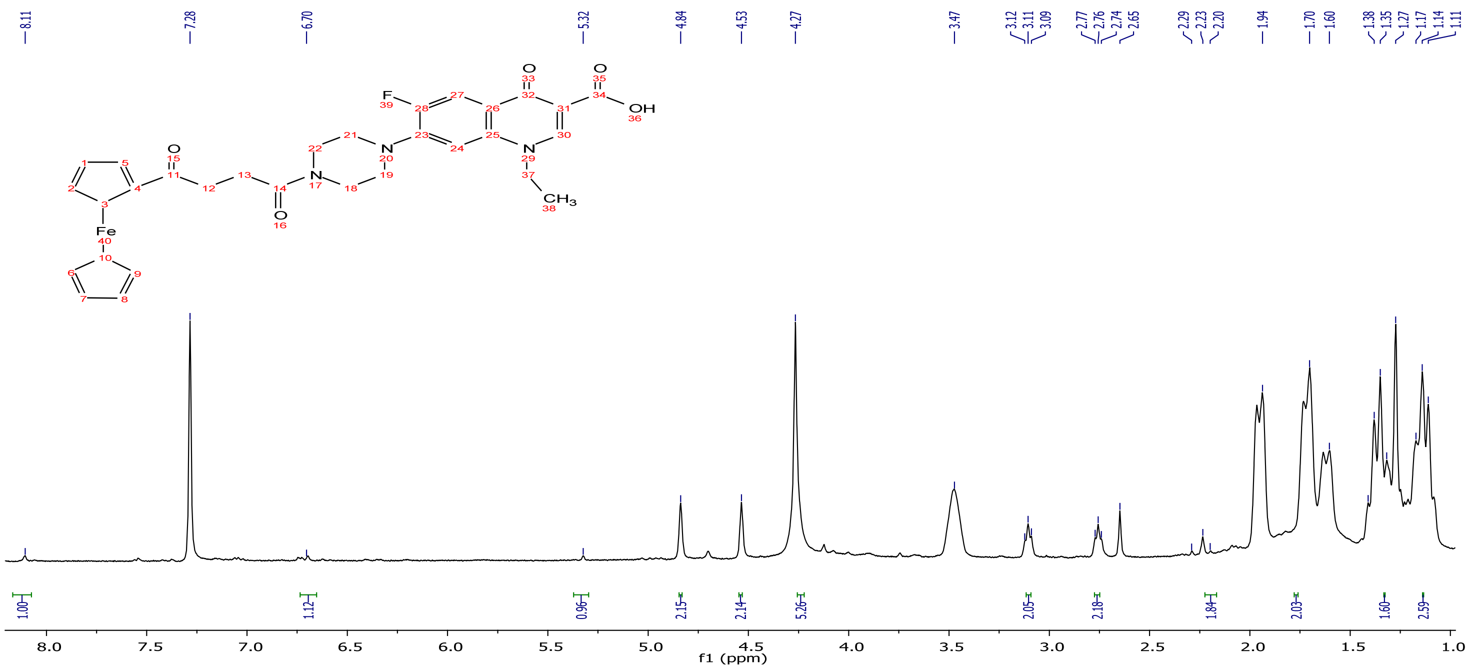

$^{1}$HNMR of norfloxacin-keto-ferrocenamide (**16**).

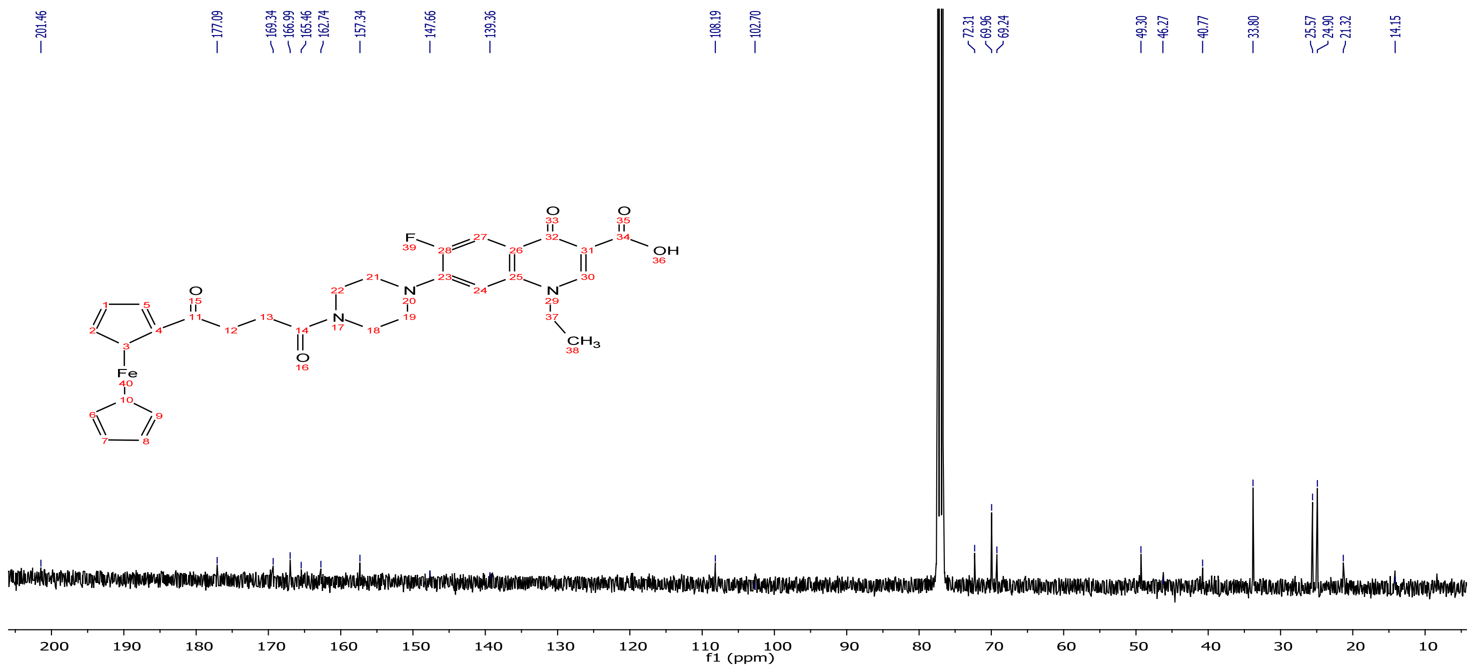

$^{13}$CNMR of norfloxacin-keto-ferrocenamide (**16**).

## Temozolo-keto-ferrocenyl butanamide (17)

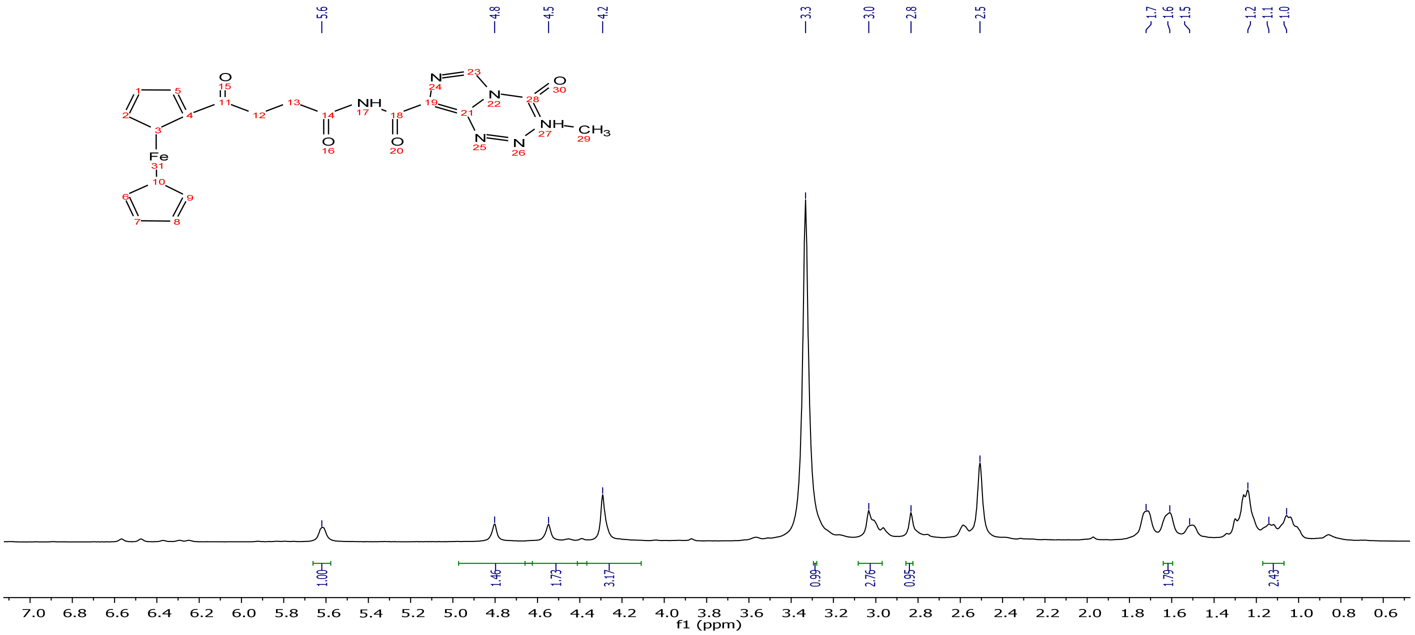


$^{1}$HNMR temo-keto-ferrocenyl butanamide (**17**).

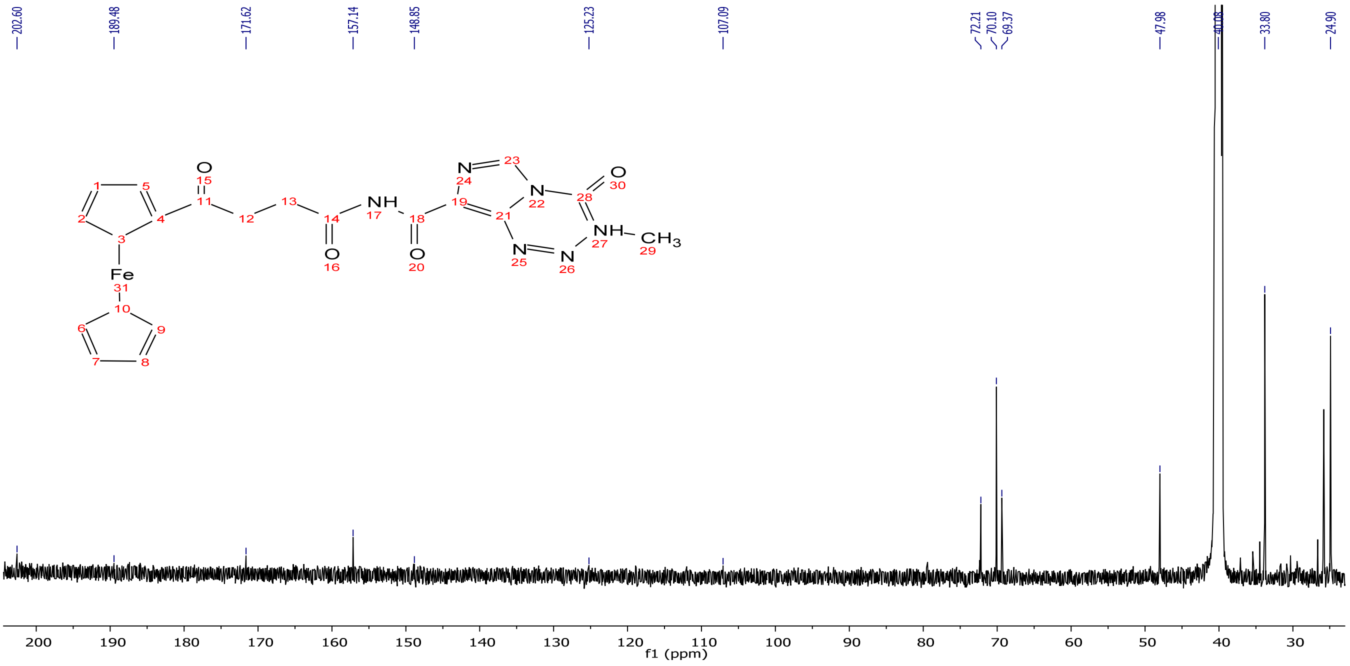


$^{13}$CNMR temo-keto-ferrocenyl butanamide (**17**).

## Metformin-keto-ferrocenyl butanamide (18)

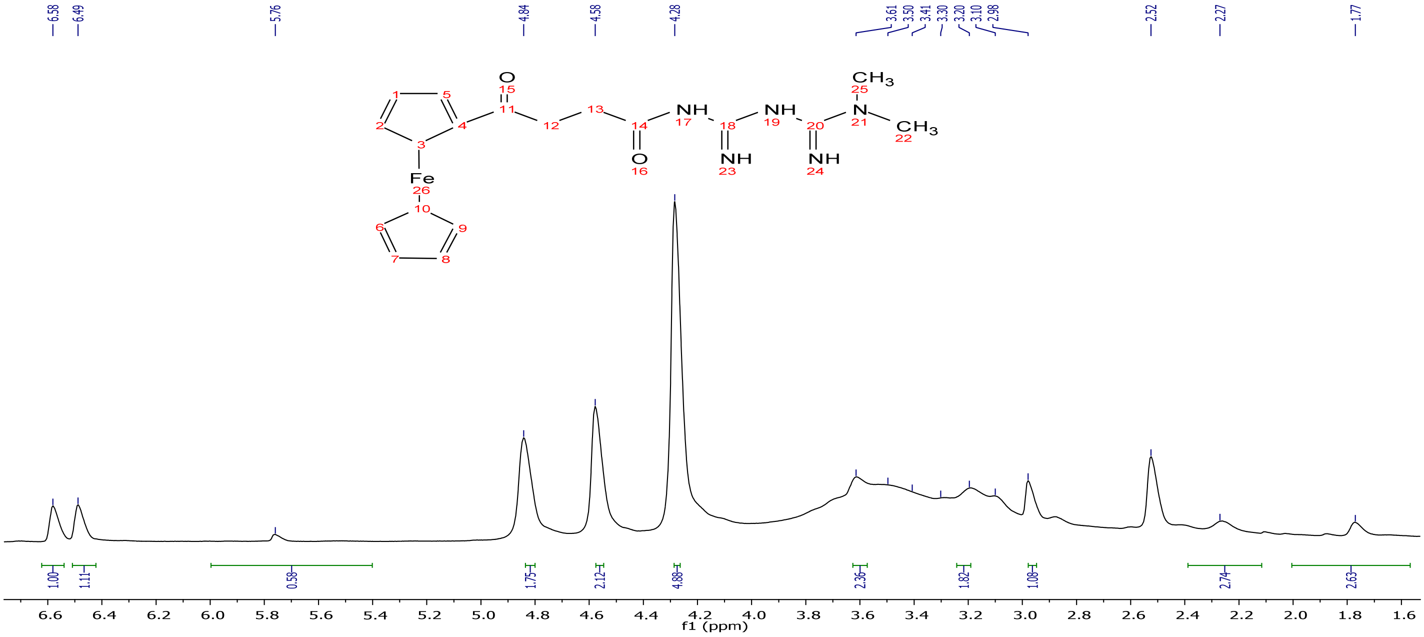

$^{1}$HNMR metformin-keto-ferrocenyl butanamide (**18**).

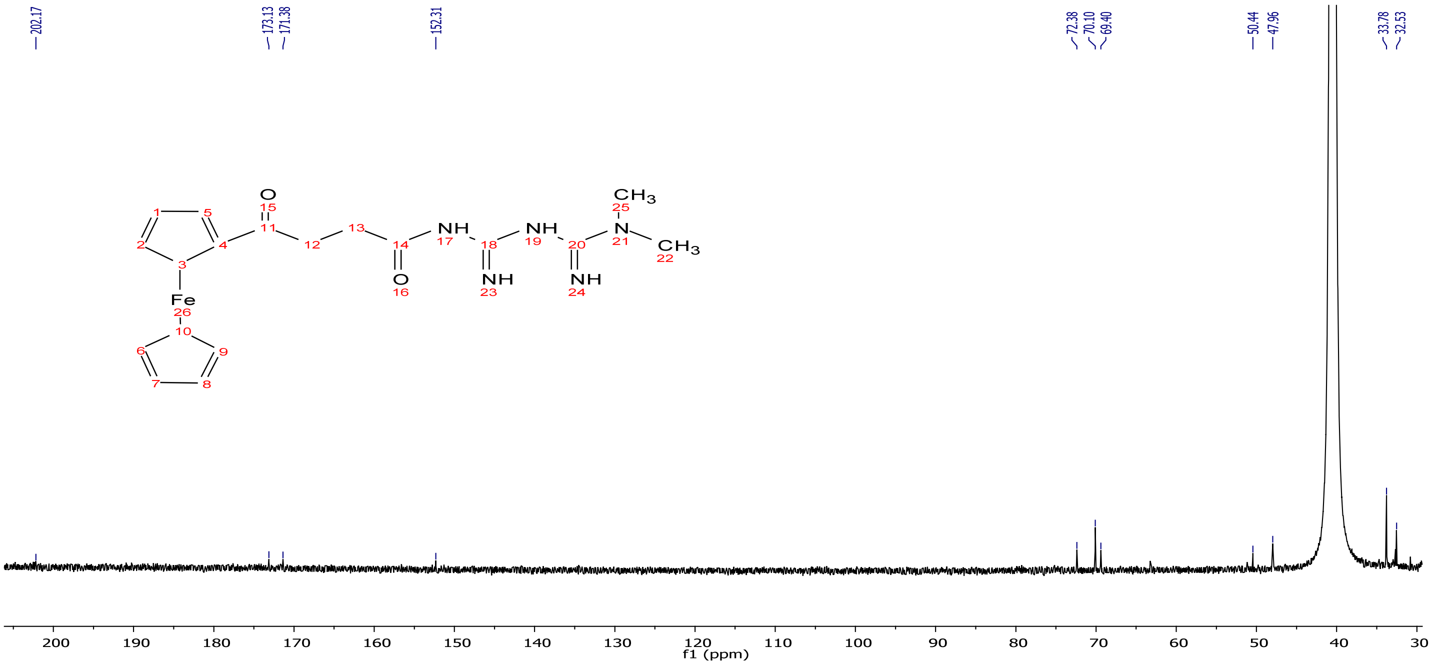

$^{13}$CNMR metformin-keto-ferrocenyl butanamide (**18**).

## Pyrimethamine-keto-ferrocenyl butanamide (19)

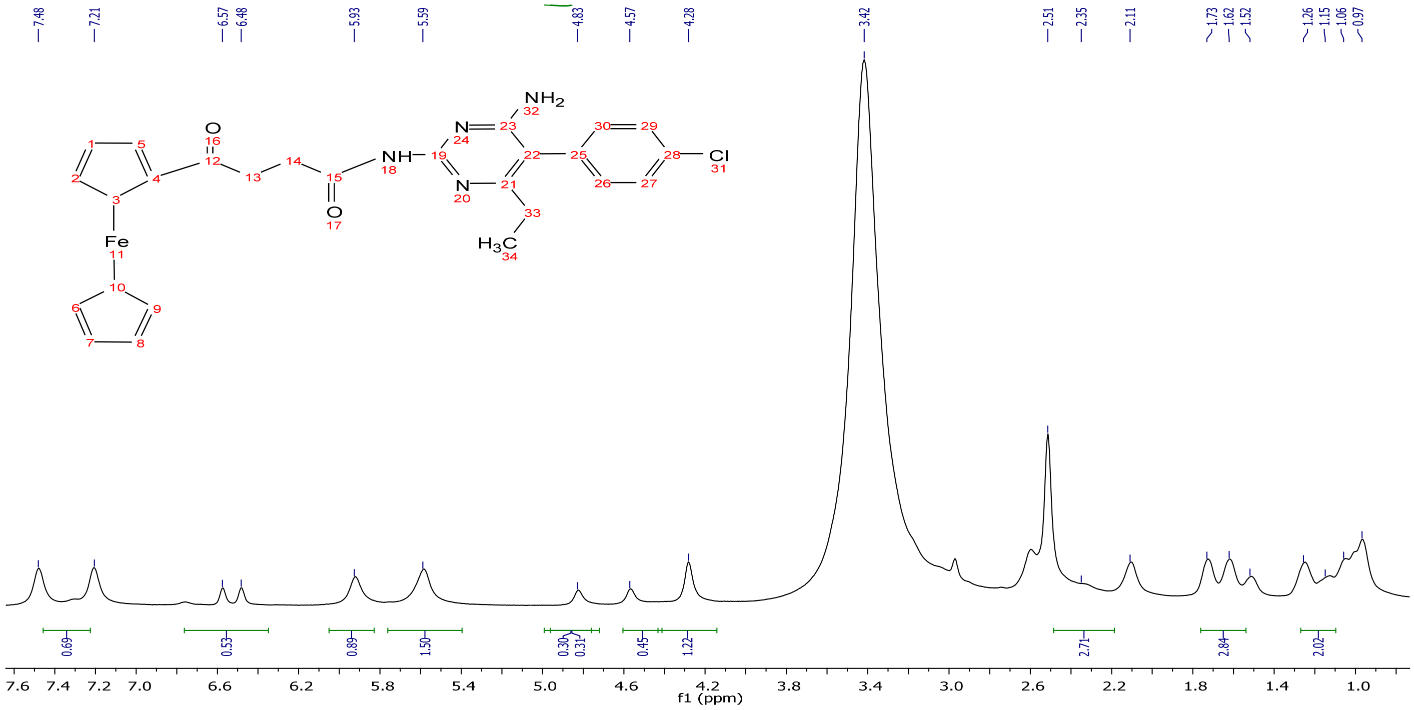


$^{1}$HNMR of pyrimethamin-keto-ferrocenyl butanamide (**19**).

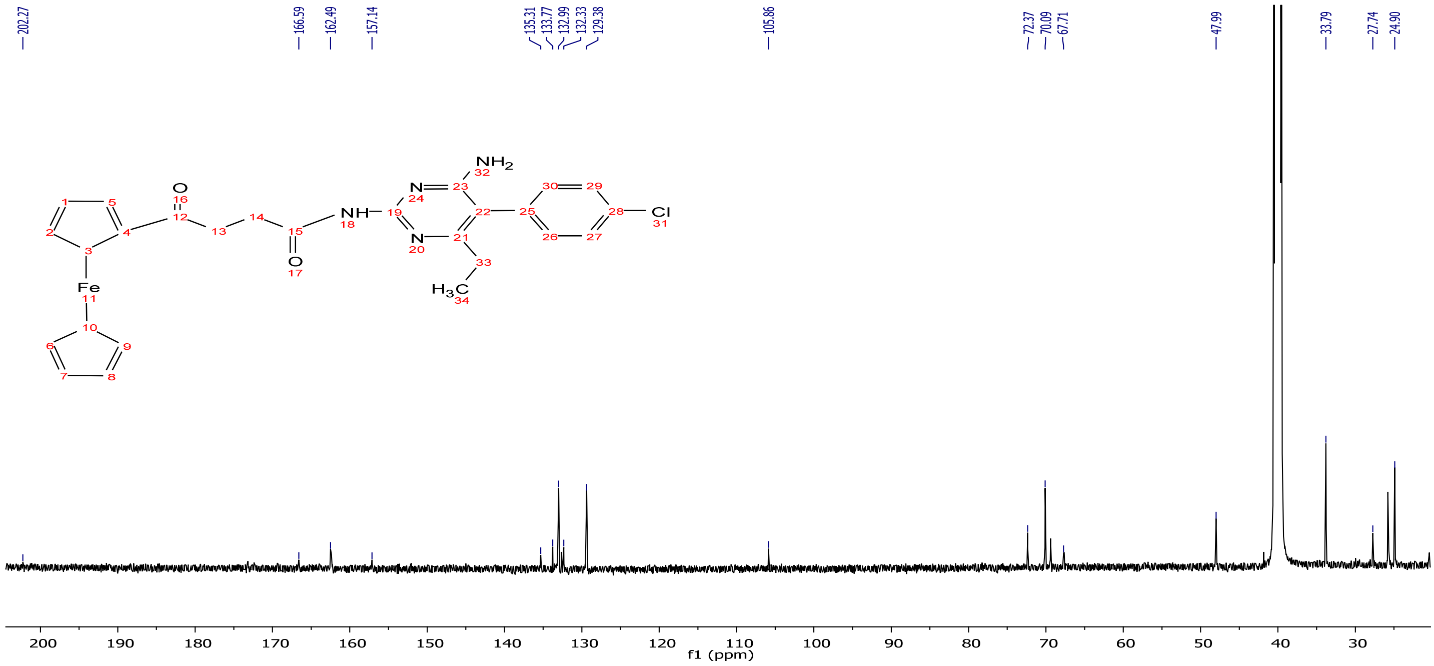


$^{13}$CNMR of pyrimethamine-keto-ferrocenyl butanamide (**19**).

## Artesunate-ferrocenoate (22)

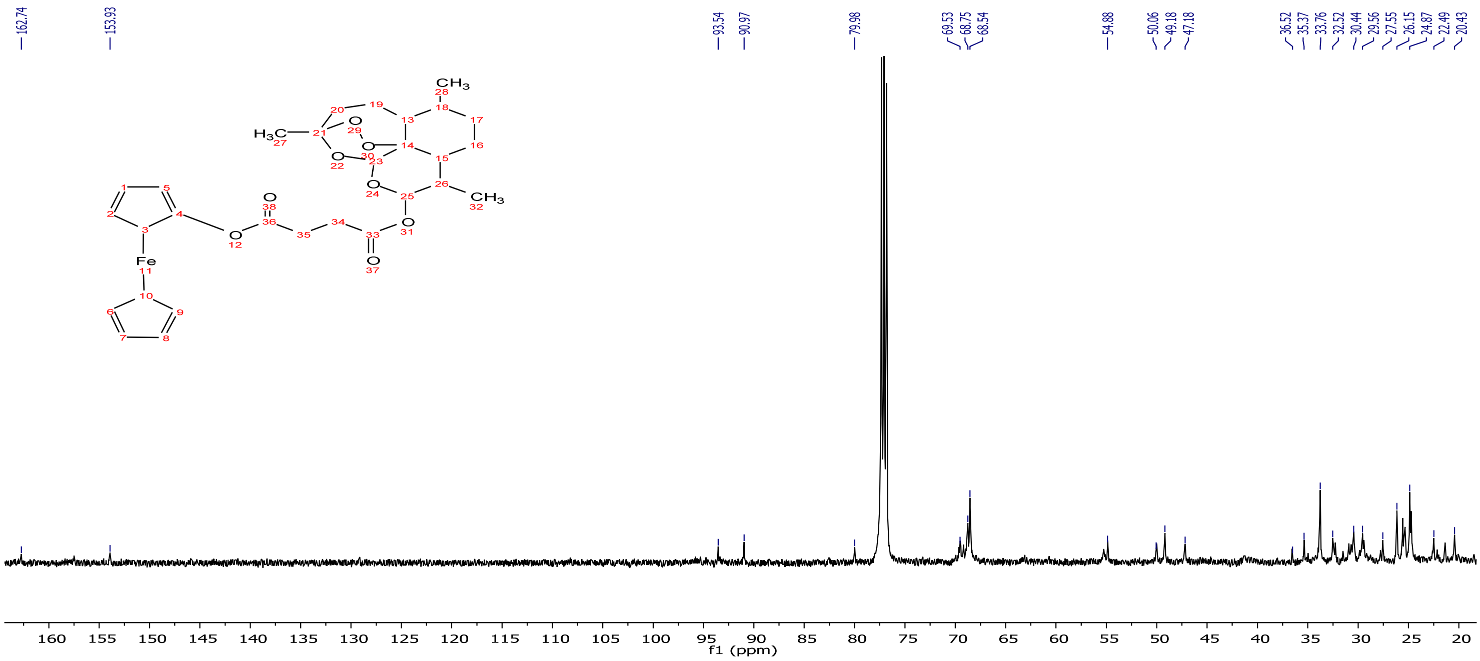

$^{1}$HNMR of artesunate-ferrocenoate (**22**).

## Cinnamic-ferrocenoate (23)

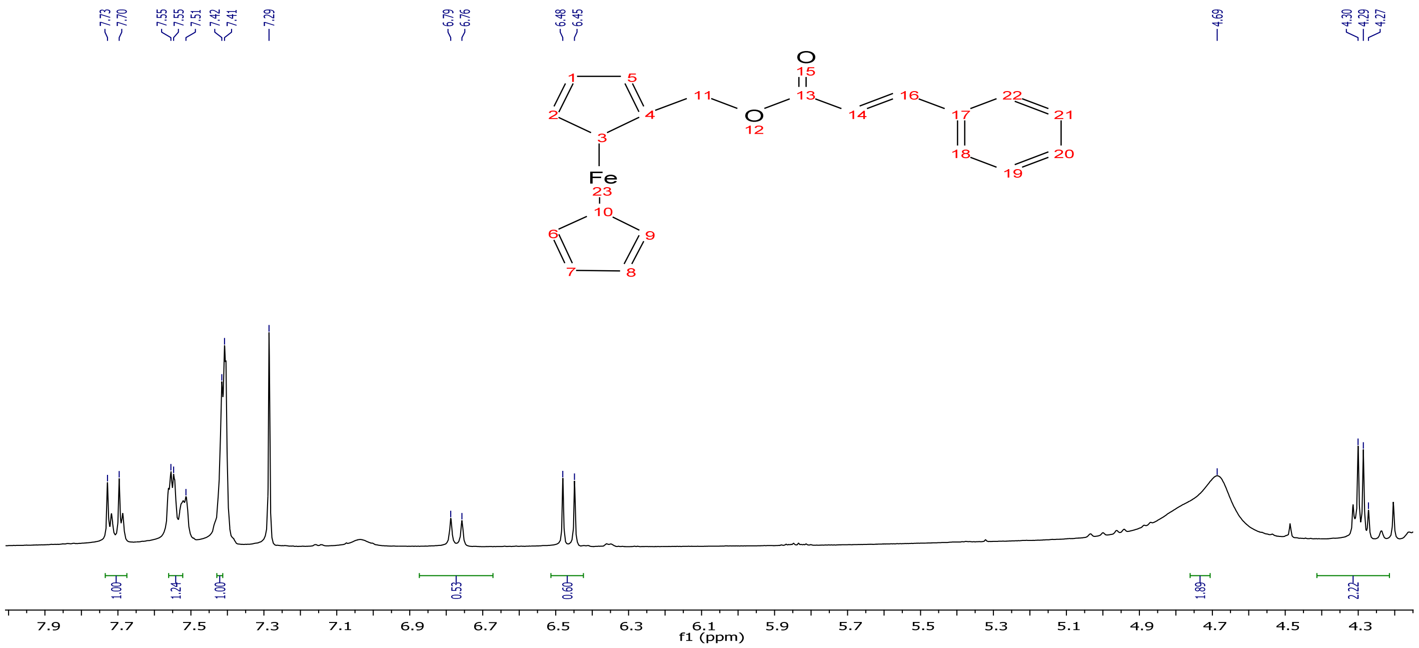

$^{1}$HNMR of cinnamic-ferrocenoate (**23**).

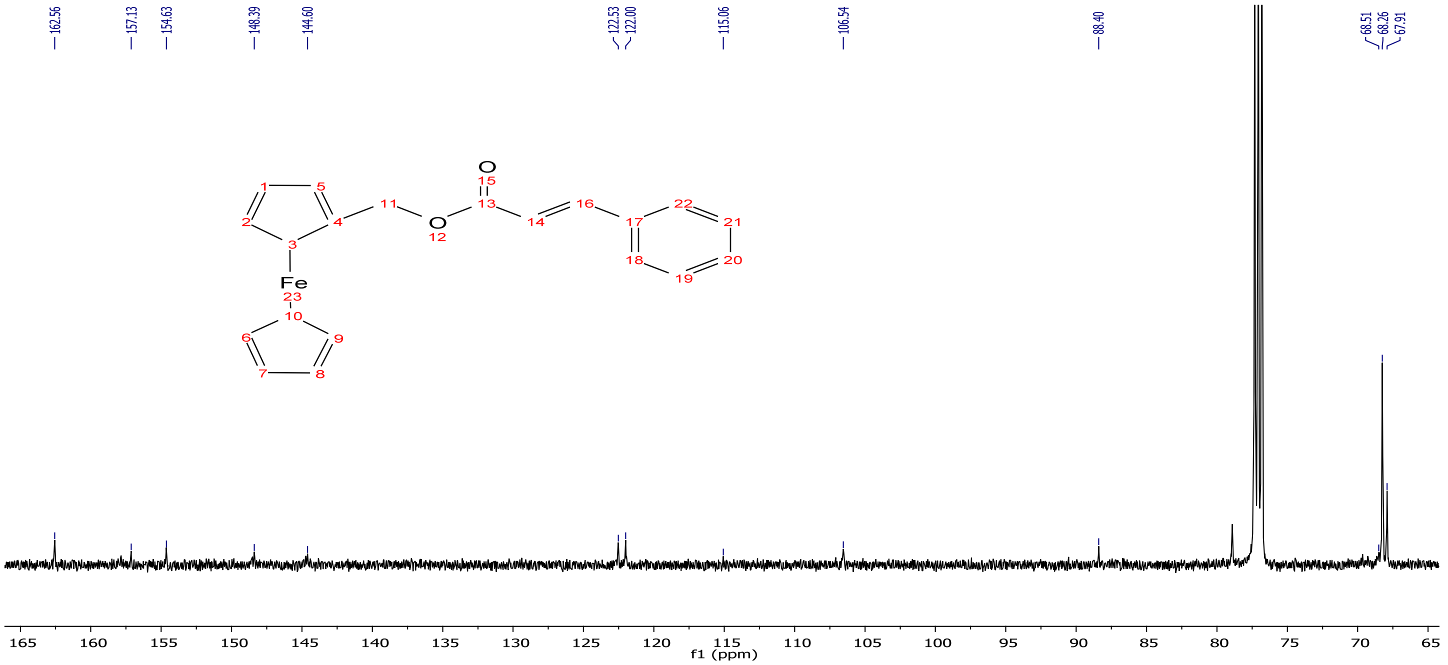

$^{13}$CNMR of cinnamic-ferrocenoate (**23**).

## **Ursolic-ferrocenoate (24)**

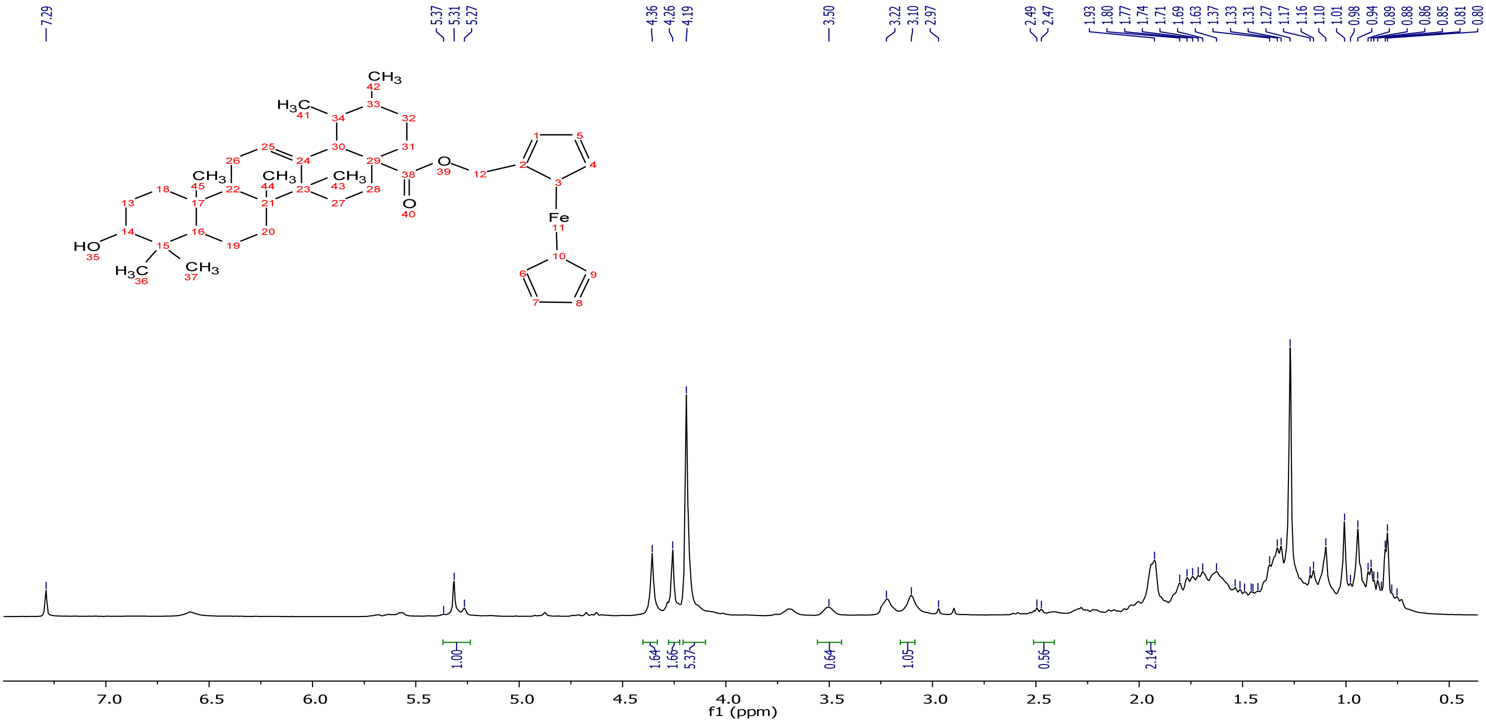


$^{1}$HNMR of ursolic-ferrocenoate (**24**).

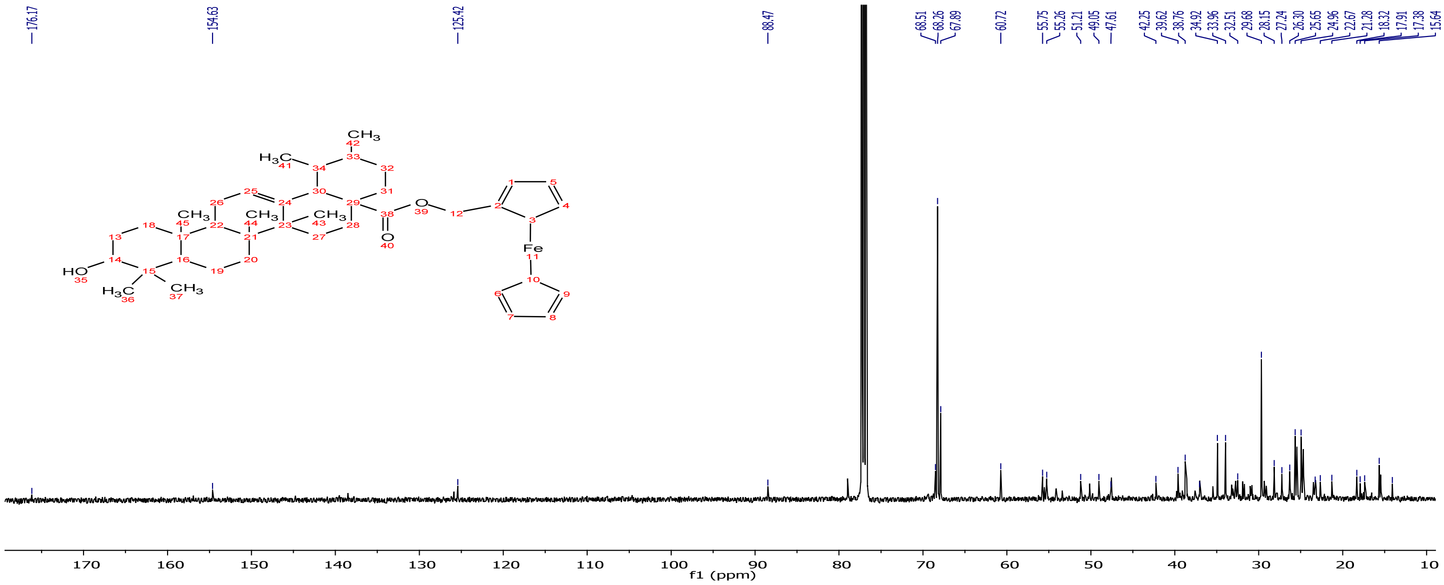


$^{13}$CNMR of ursolic-ferrocenoate (**24**).

# Oleanolic-ferrocenoate (25)

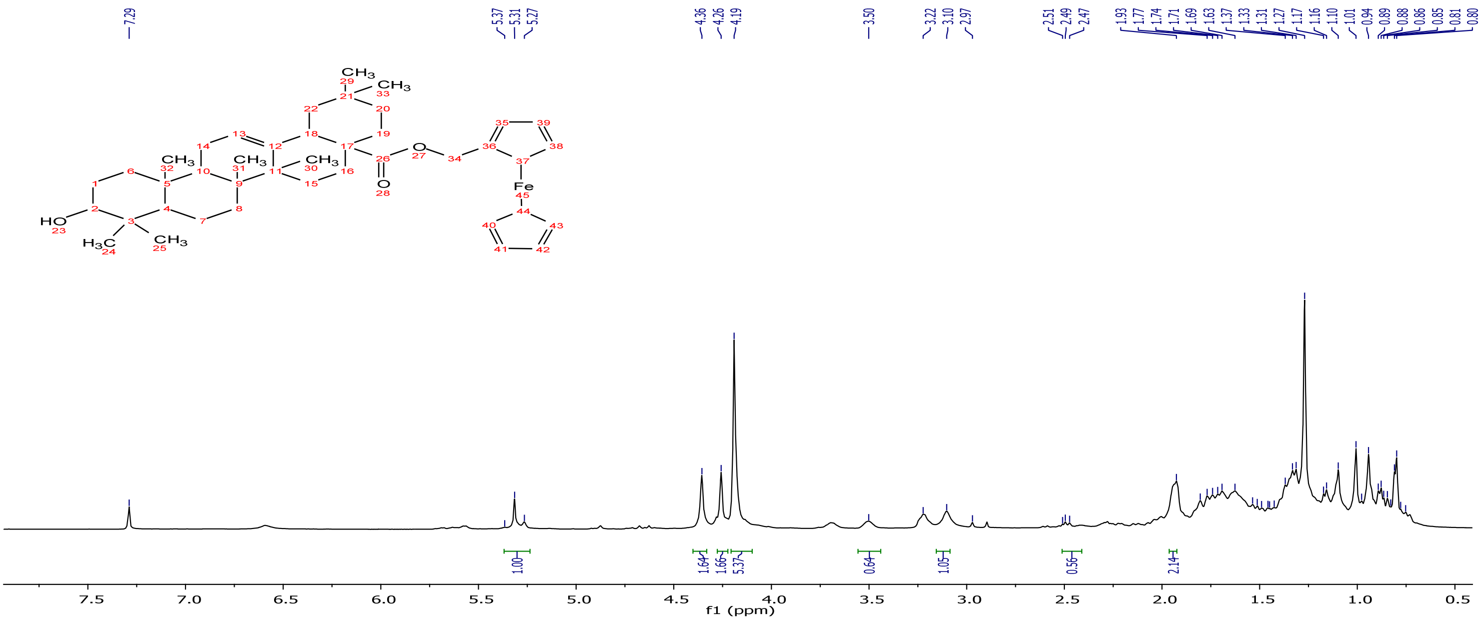


$^{1}$HNMR of oleanolic-ferrocenoate (**25**).

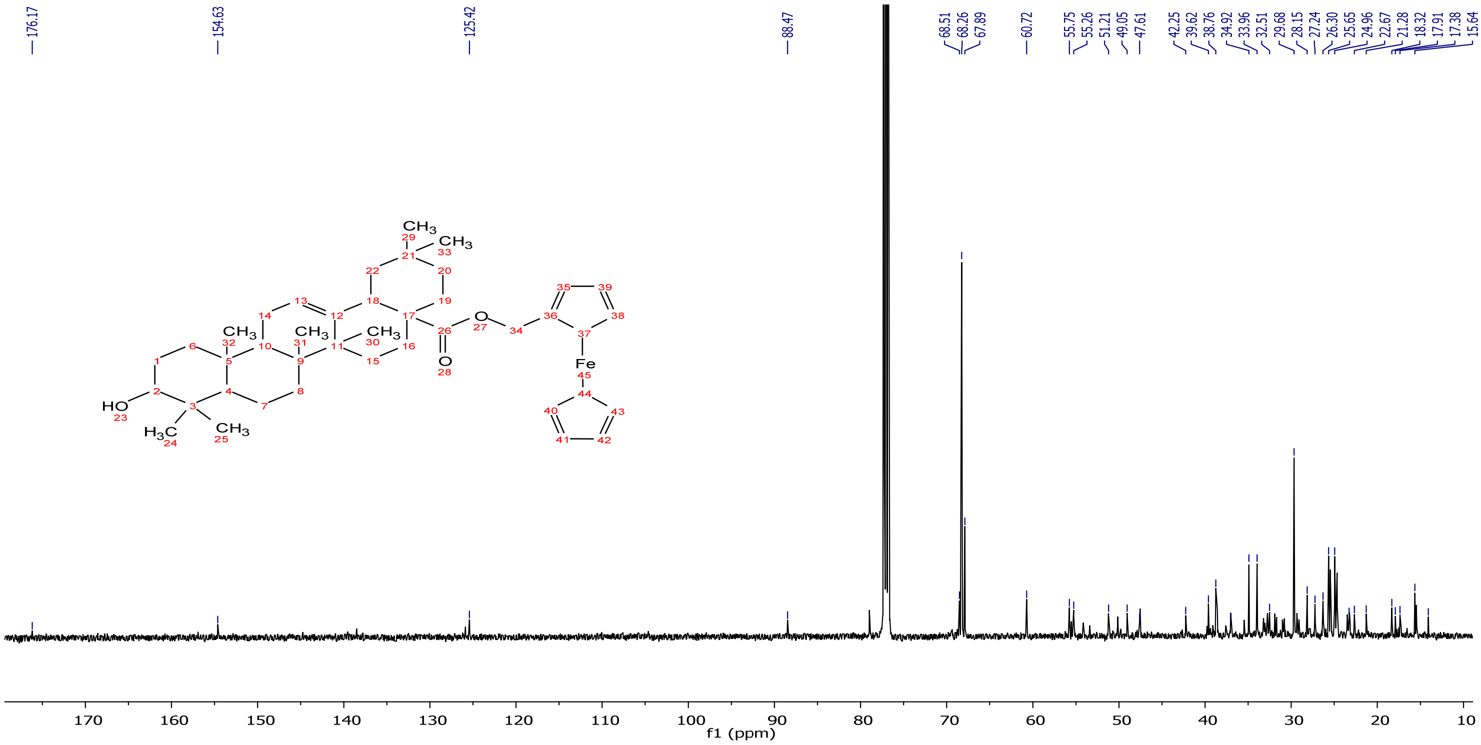


$^{13}$CNMR of oleanolic-ferrocenoate (**25**).

**Methotrexate-ferrocenoate (26)**

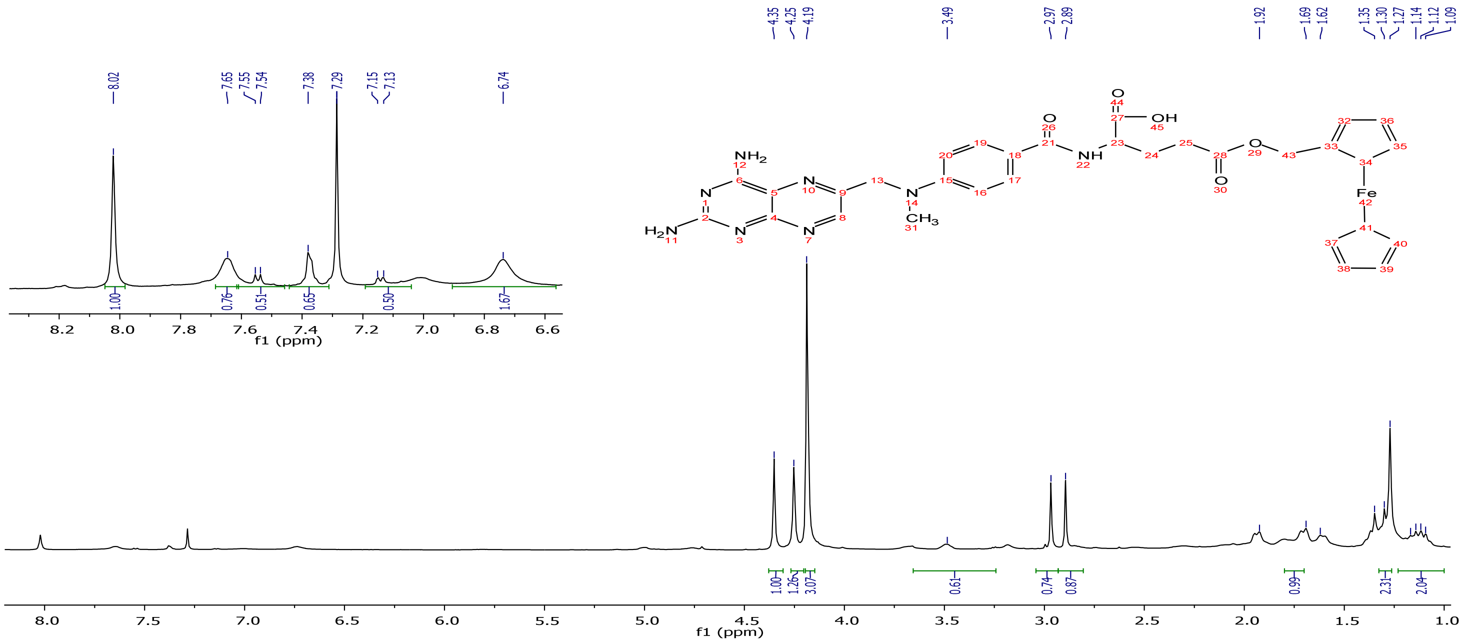

$^{1}$HNMR of methotrexate-ferrocenoate (**26**).

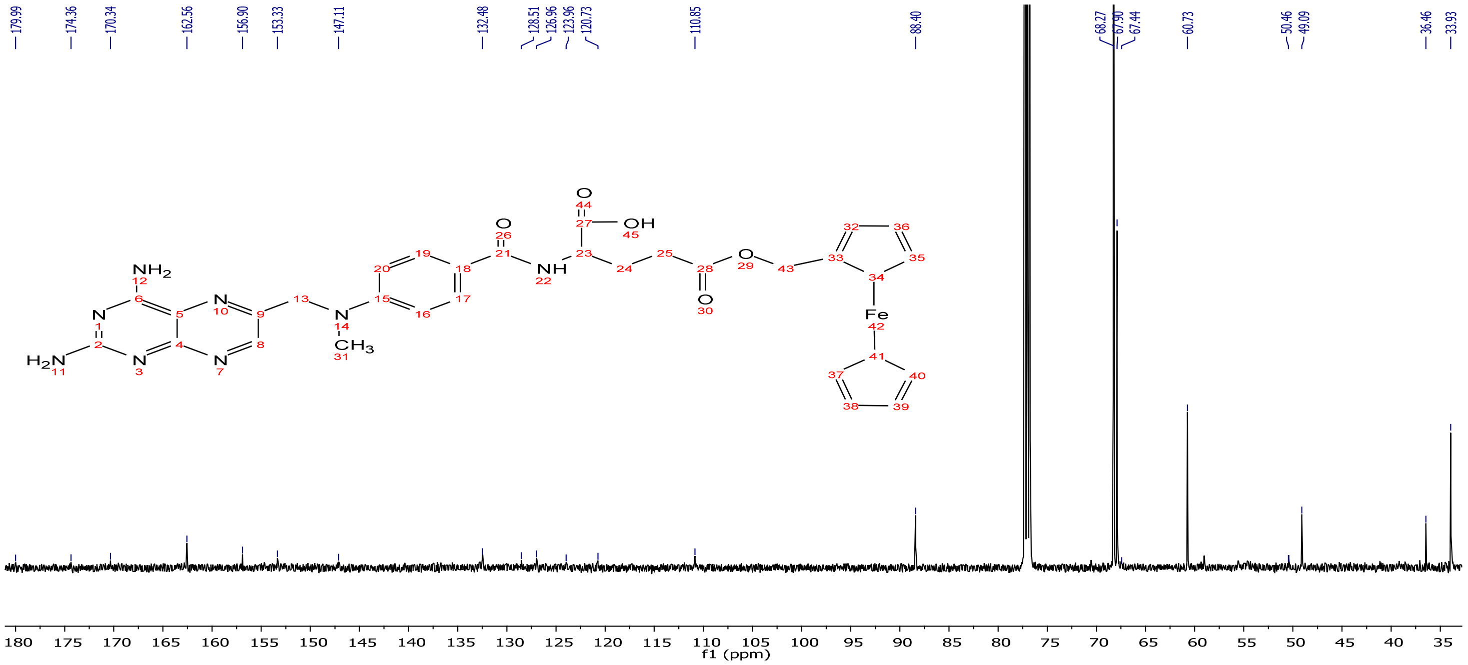

$^{13}$CNMR of methotrexate-ferrocenoate (**26**).

# FTIR of compounds 8, 21, and hybrid compounds (9-19and 22-26)

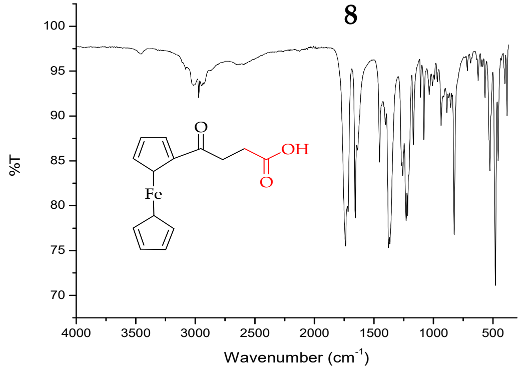


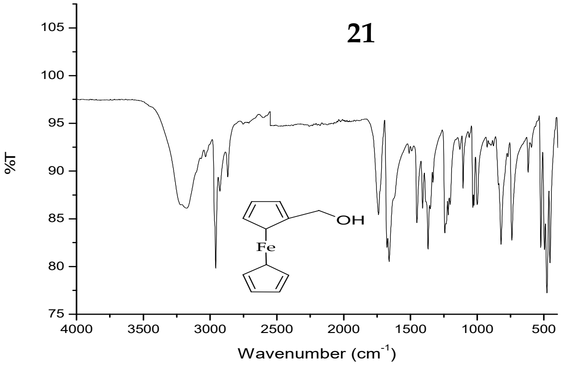


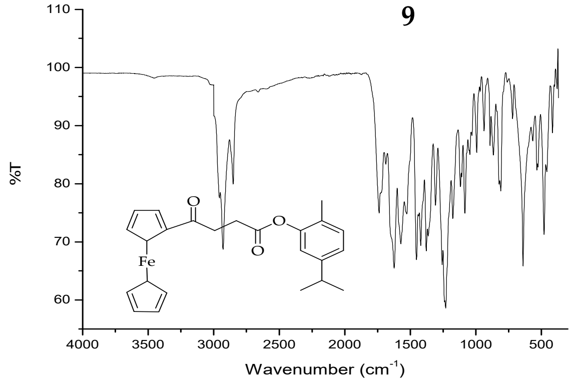


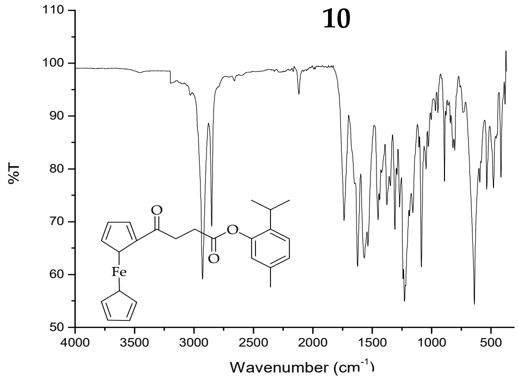


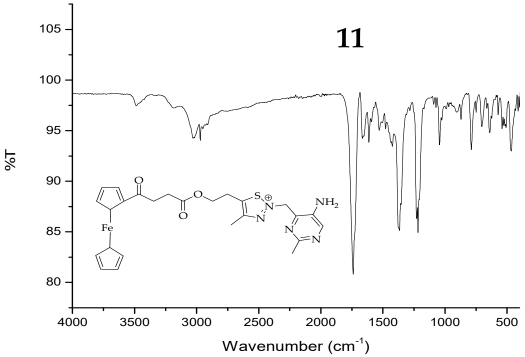


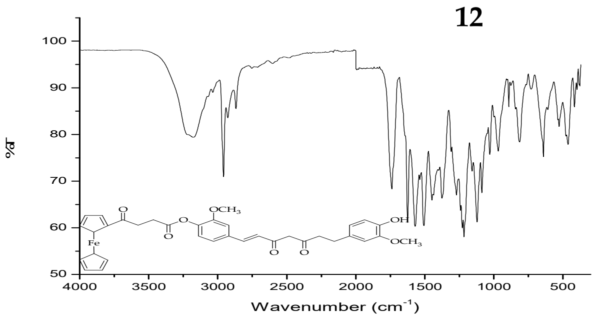


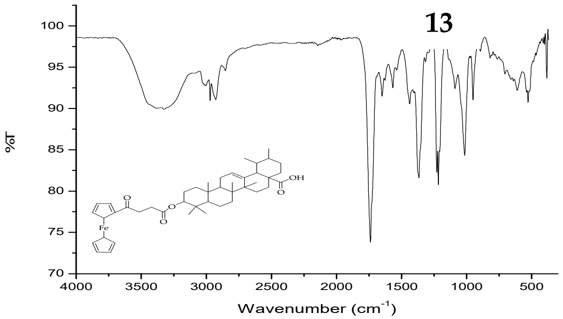


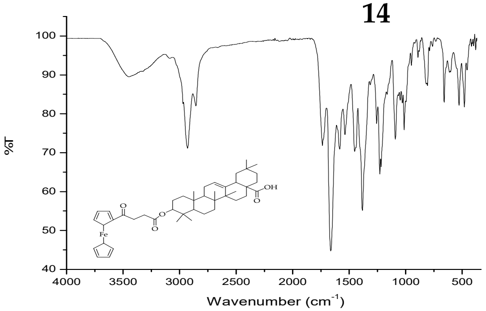


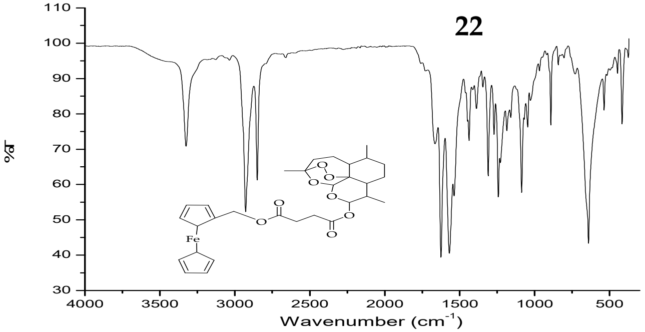

23

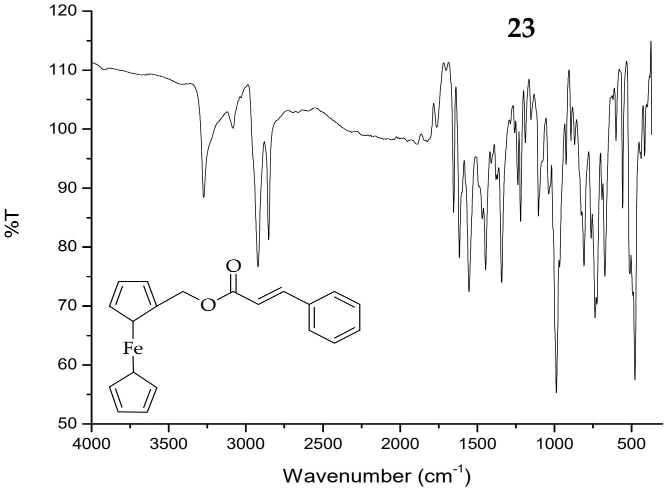


24

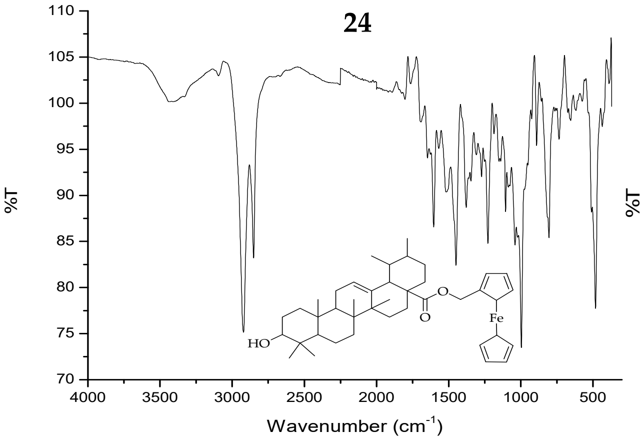


25

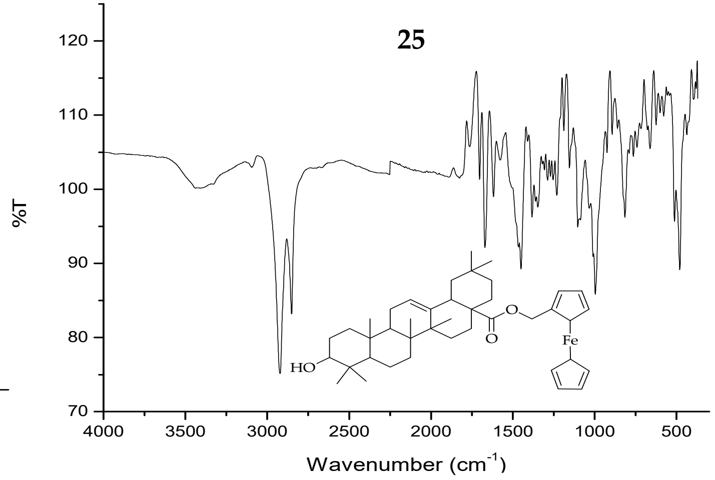


26

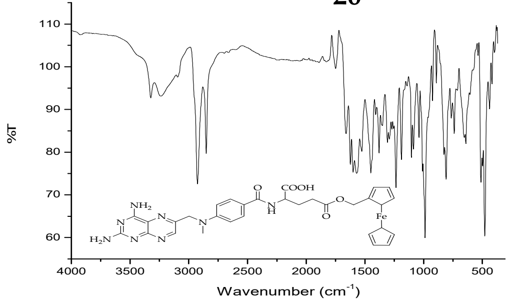


15

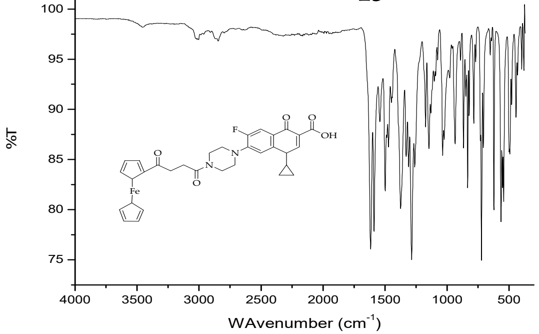


16

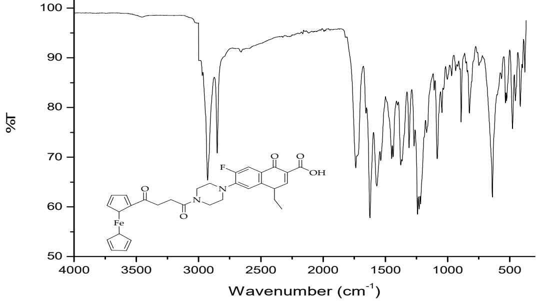


17

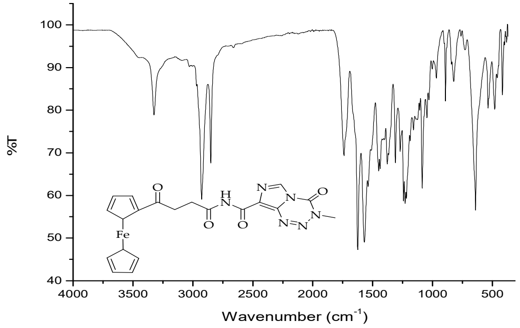


18

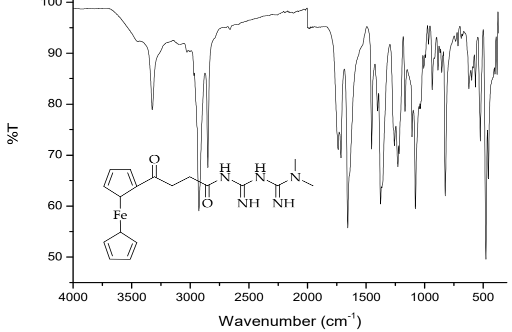


19

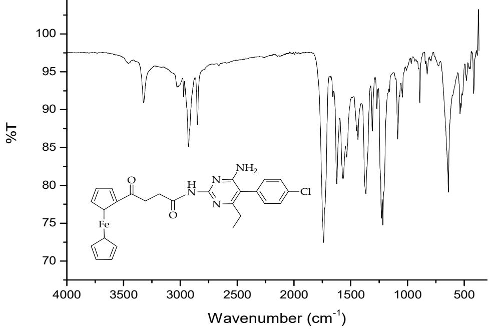

**LC-MS of the hybrid compounds (9-19 and 22-26)**

**9**

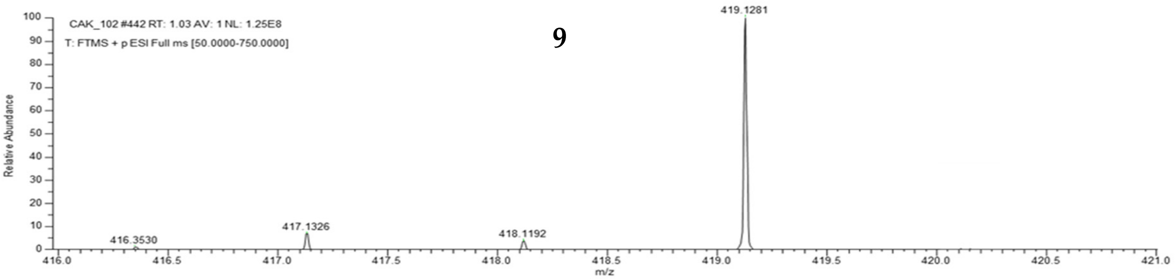


**10**

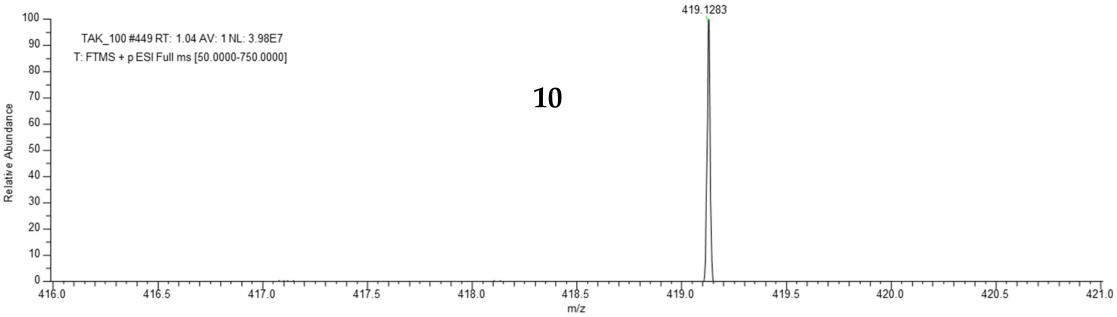


**11**

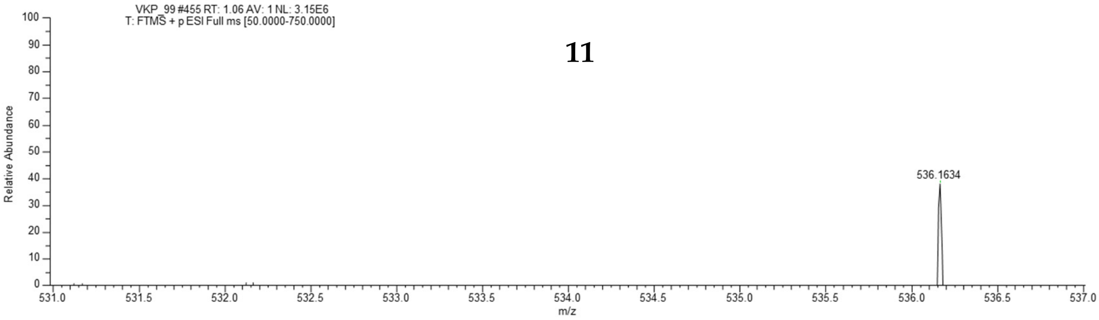


**12**

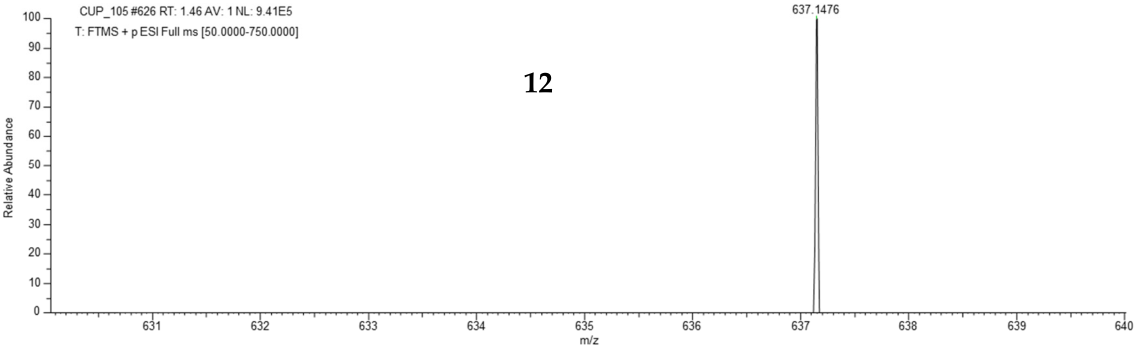


**13**

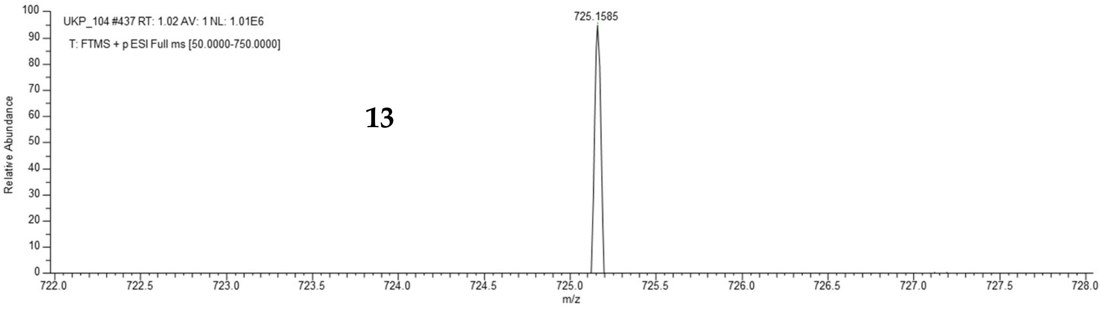

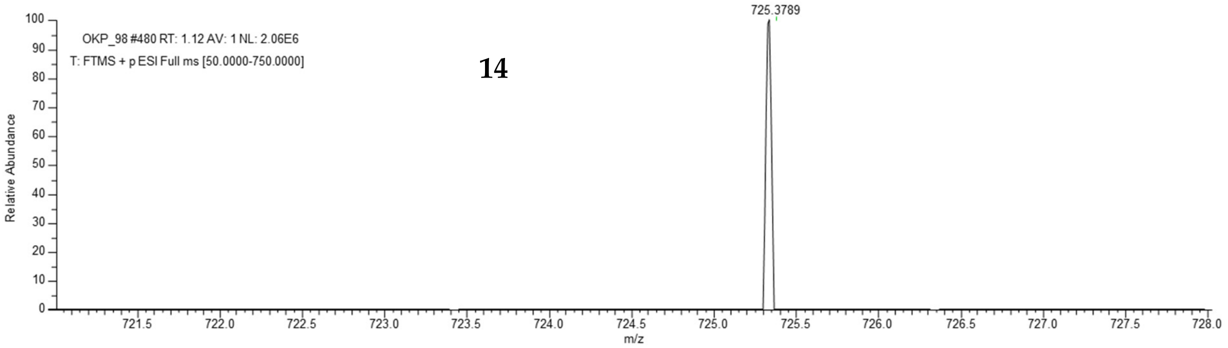
OKP_98 #480 RT: 1.12 AV: 1 NL: 2.06E6
T: FTMS + p ESI Full ms [50.0000-750.0000]
14
725.3789
Relative Abundance
m/z

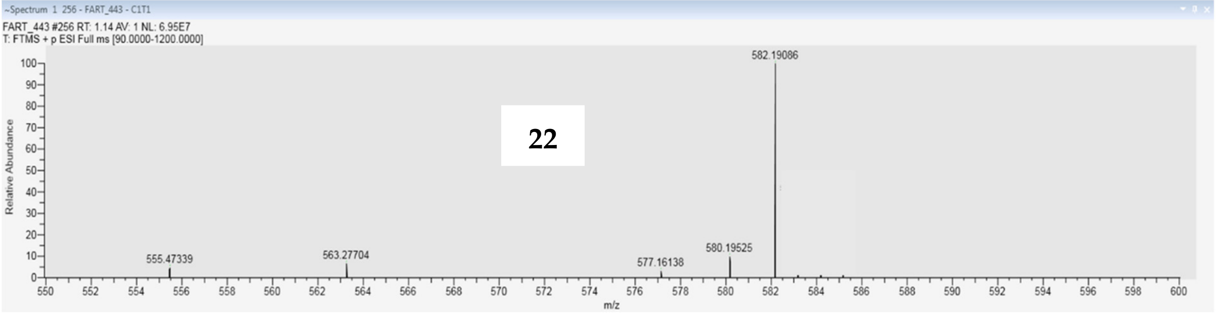
~Spectrum 1 256 - FART_443 - C1T1
FART_443 #256 RT: 1.14 AV: 1 NL: 6.95E7
T: FTMS + p ESI Full ms [90.0000-1200.0000]
22
582.19086
555.47339
563.27704
577.16138
580.19525
Relative Abundance
m/z

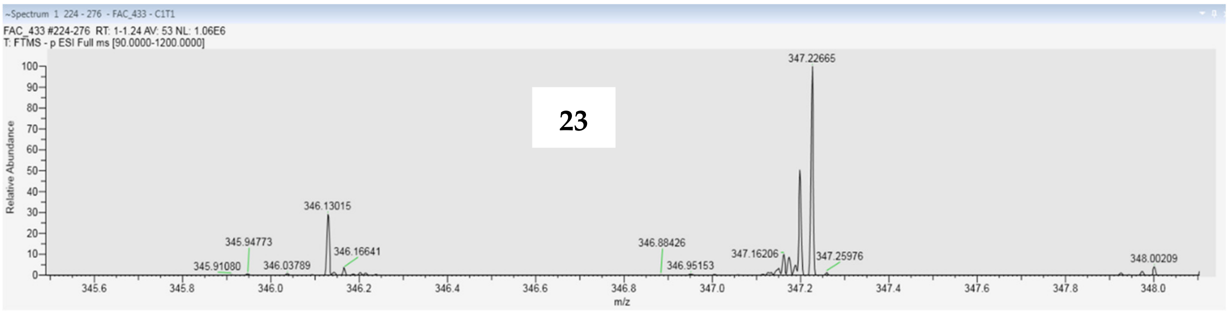
~Spectrum 1 224 - 276 - FAC_433 - C1T1
FAC_433 #224-276 RT: 1-1.24 AV: 53 NL: 1.06E6
T: FTMS - p ESI Full ms [90.0000-1200.0000]
23
347.22665
346.13015
345.94773
345.91080
346.03789
346.16641
346.88426
346.95153
347.16206
347.25976
348.00209
Relative Abundance
m/z

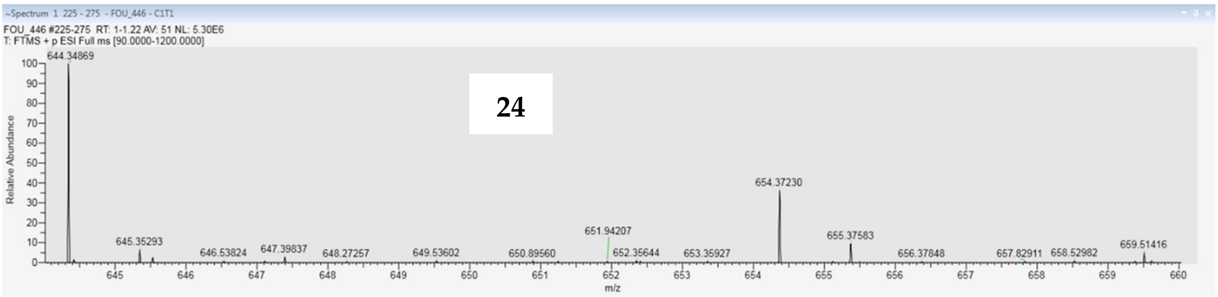
~Spectrum 1 225 - 275 - FOU_446 - C1T1
FOU_446 #225-275 RT: 1-1.22 AV: 51 NL: 5.30E6
T: FTMS + p ESI Full ms [90.0000-1200.0000]
24
644.34869
645.35293
646.53824
647.39837
648.27257
649.53602
650.89560
651.94207
652.35644
653.35927
654.37230
655.37583
656.37848
657.82911
658.52982
659.51416
Relative Abundance
m/z

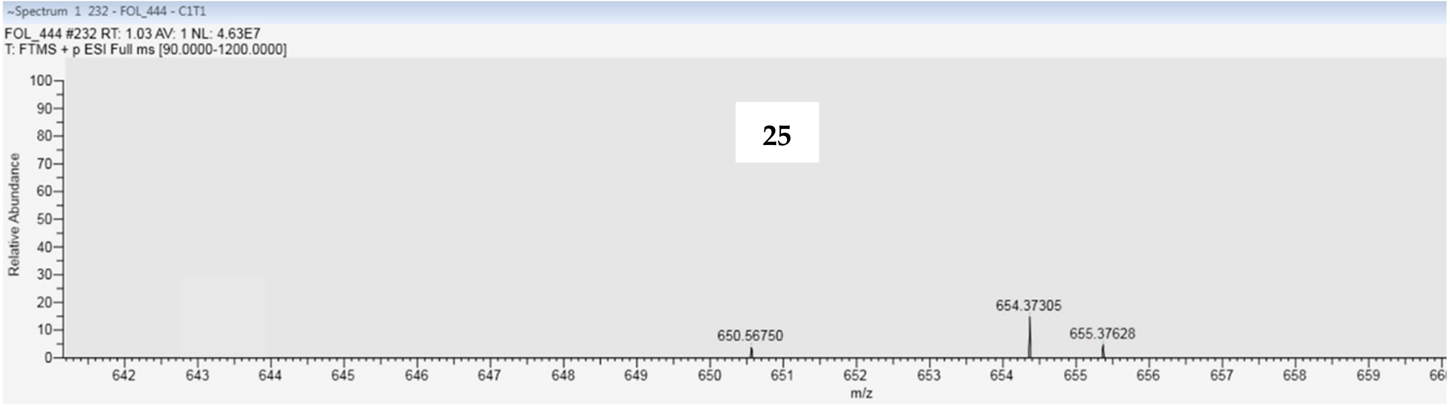
~Spectrum 1 232 - FOL_444 - C1T1
FOL_444 #232 RT: 1.03 AV: 1 NL: 4.63E7
T: FTMS + p ESI Full ms [90.0000-1200.0000]
25
650.56750
654.37305
655.37628
Relative Abundance
m/z

26

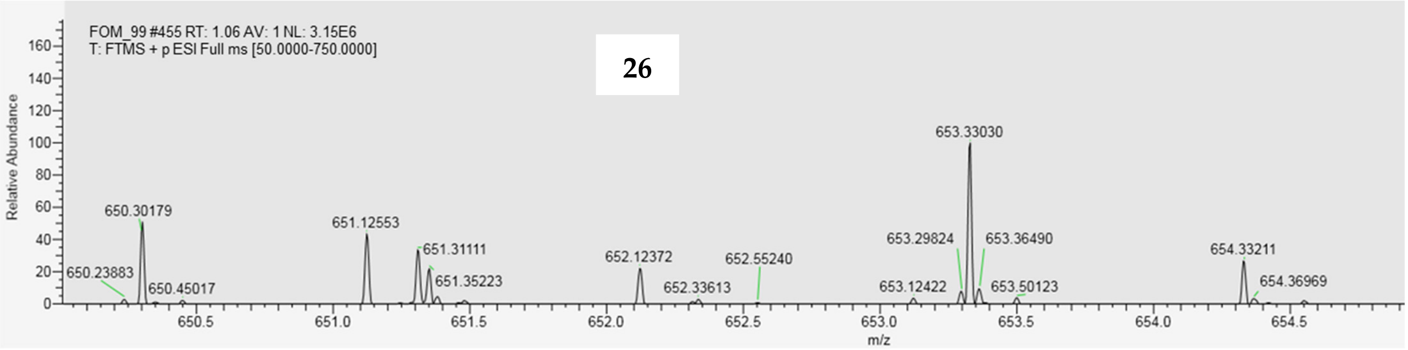
FOM_99 #455 RT: 1.06 AV: 1 NL: 3.15E6
T: FTMS + p ESI Full ms [50.0000-750.0000]
Relative Abundance
m/z
650.30179
650.23883
650.45017
651.12553
651.31111
651.35223
652.12372
652.33613
652.55240
653.29824
653.33030
653.36490
653.12422
653.50123
654.33211
654.36969

15

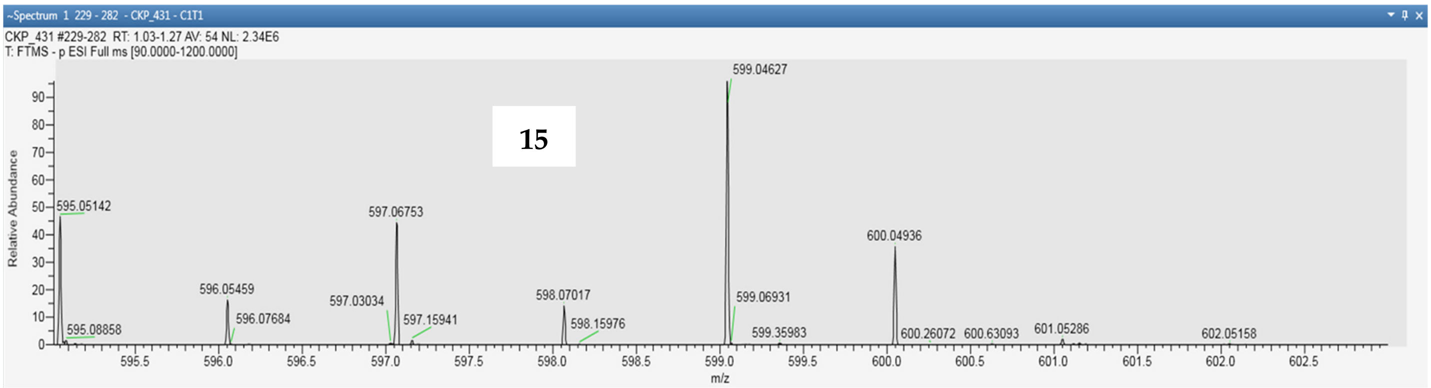
~Spectrum 1 229 - 282 - CKP_431 - C1T1
CKP_431 #229-282 RT: 1.03-1.27 AV: 54 NL: 2.34E6
T: FTMS - p ESI Full ms [90.0000-1200.0000]
Relative Abundance
m/z
595.05142
595.08858
596.05459
596.07684
597.03034
597.06753
597.15941
598.07017
598.15976
599.04627
599.06931
599.35983
600.04936
600.26072
600.63093
601.05286
602.05158

16

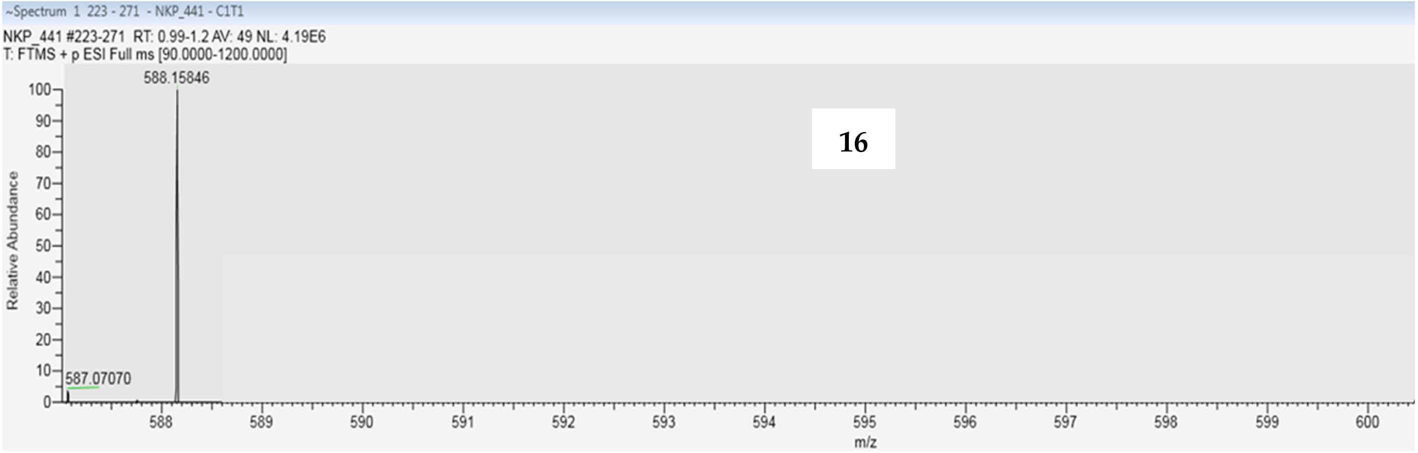
~Spectrum 1 223 - 271 - NKP_441 - C1T1
NKP_441 #223-271 RT: 0.99-1.2 AV: 49 NL: 4.19E6
T: FTMS + p ESI Full ms [90.0000-1200.0000]
Relative Abundance
m/z
588.15846
587.07070

17

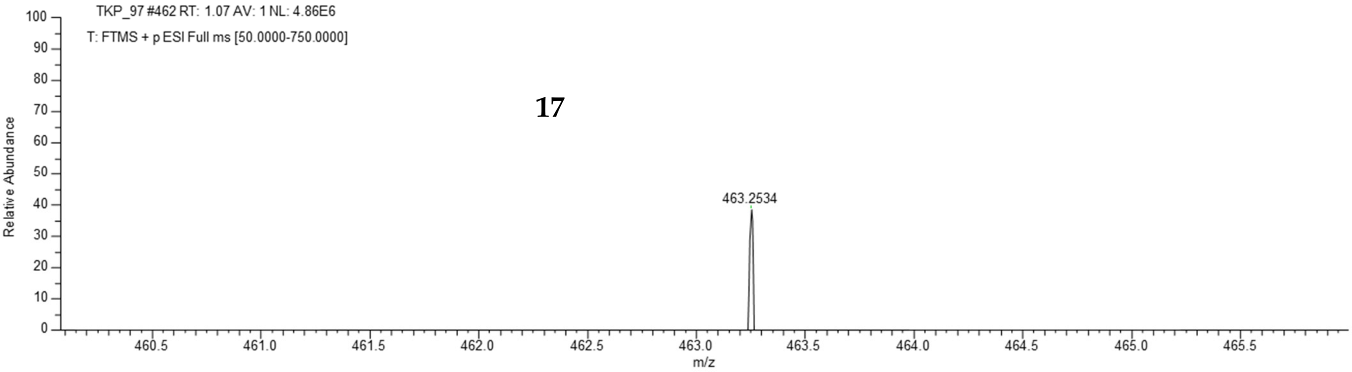
TKP_97 #462 RT: 1.07 AV: 1 NL: 4.86E6
T: FTMS + p ESI Full ms [50.0000-750.0000]
Relative Abundance
m/z
463.2534

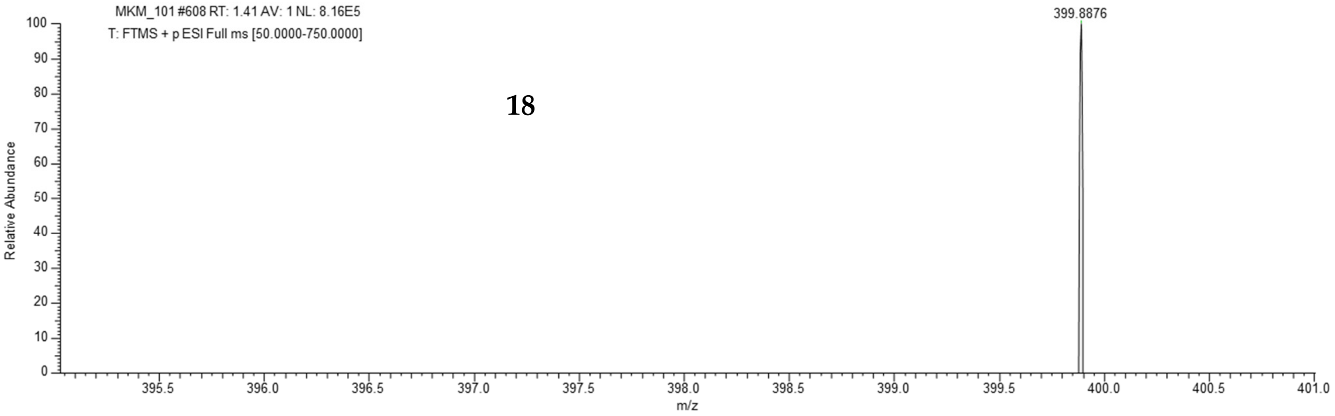

MKM_101 #608 RT: 1.41 AV: 1 NL: 8.16E5
T: FTMS + p ESI Full ms [50.0000-750.0000]
399.8876
Relative Abundance
m/z


18

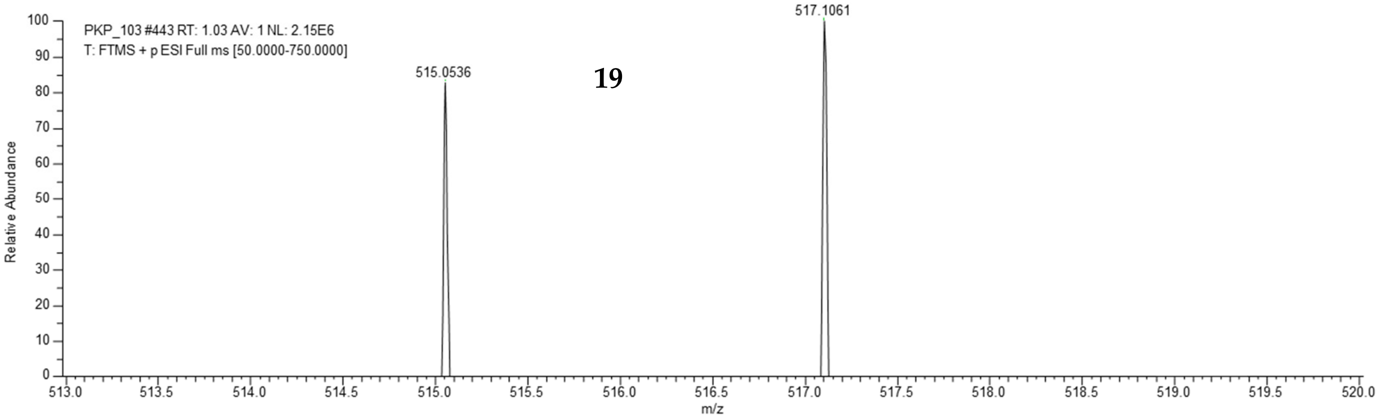

PKP_103 #443 RT: 1.03 AV: 1 NL: 2.15E6
T: FTMS + p ESI Full ms [50.0000-750.0000]
515.0536
517.1061
Relative Abundance
m/z


19

**% Cell Viability and IC50 (μg/mL) values of POA, Compound 16, and hybrid compounds (9-19 and 22-26).**

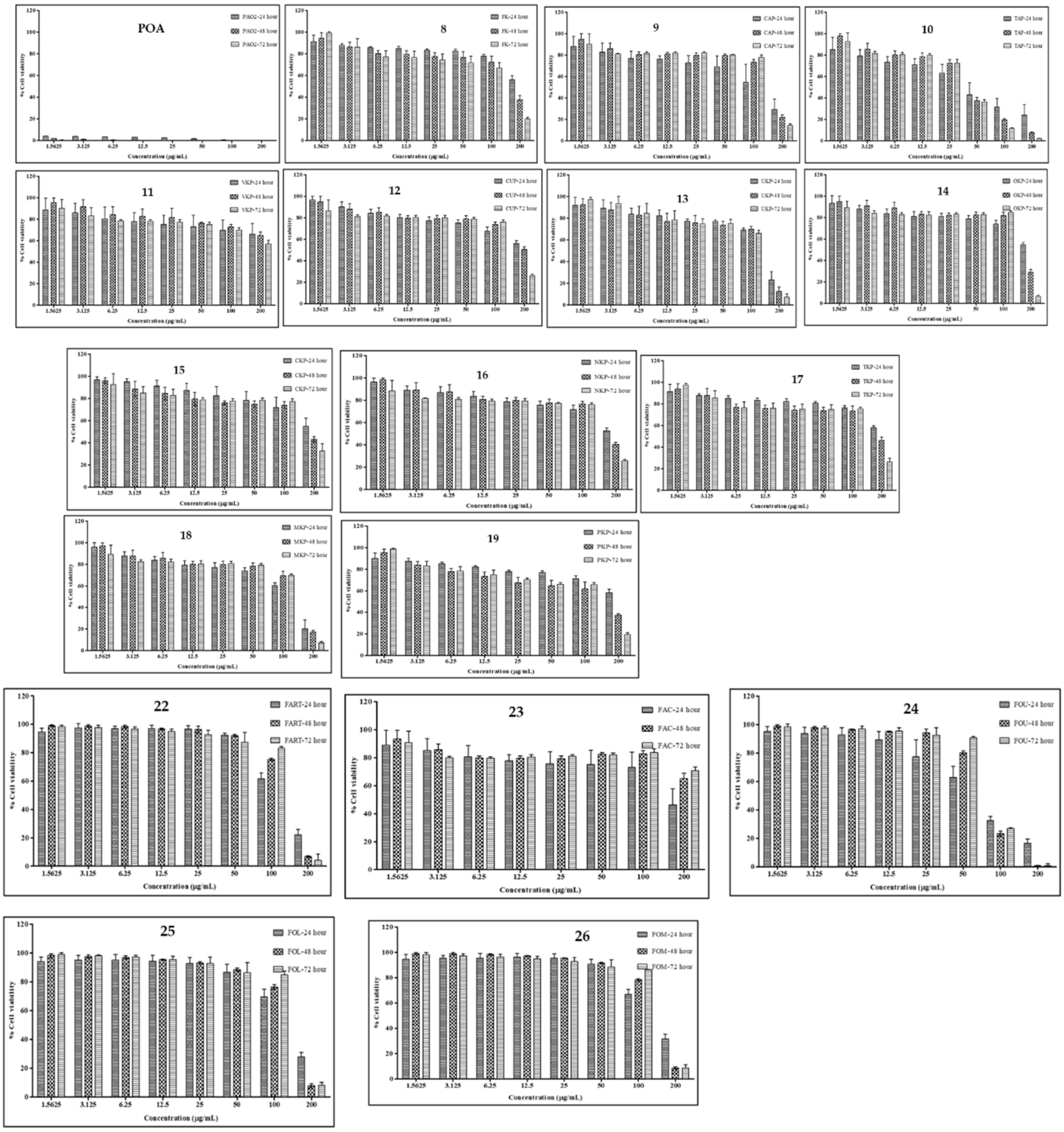

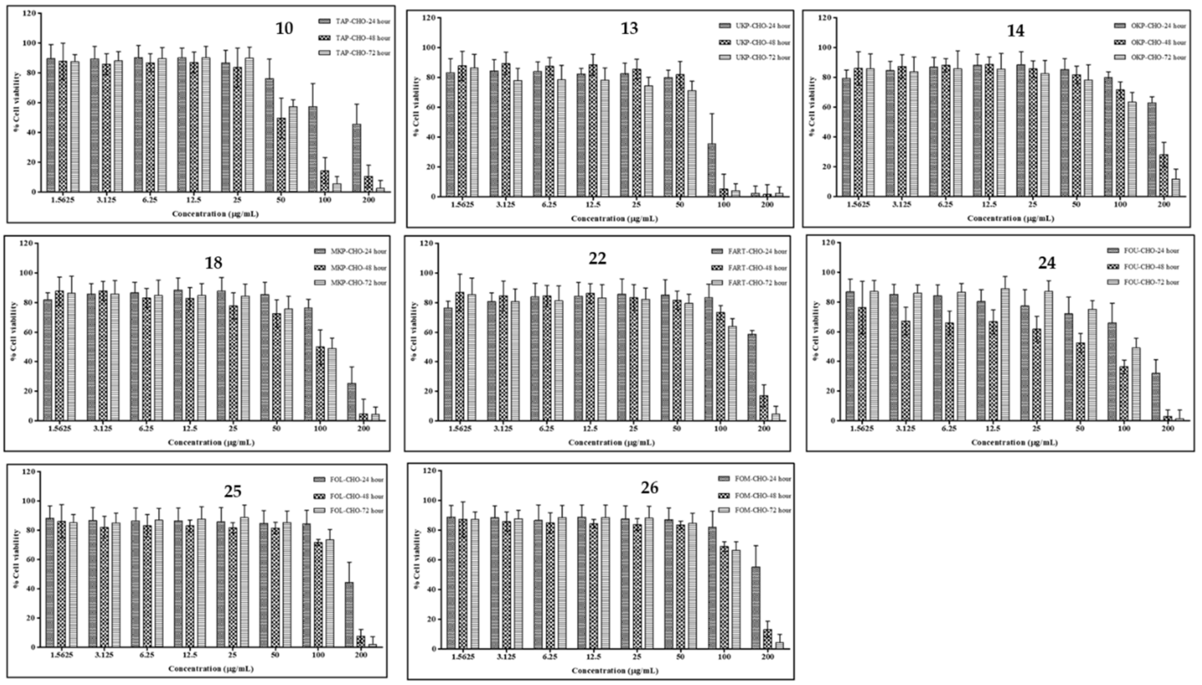


The % Cell viability of hybrid (**10**, **13**, **14**, **18, 22**, **24**, **25**, **and 26**) against Chinese Hamster Ovary cancer cells (CHO).

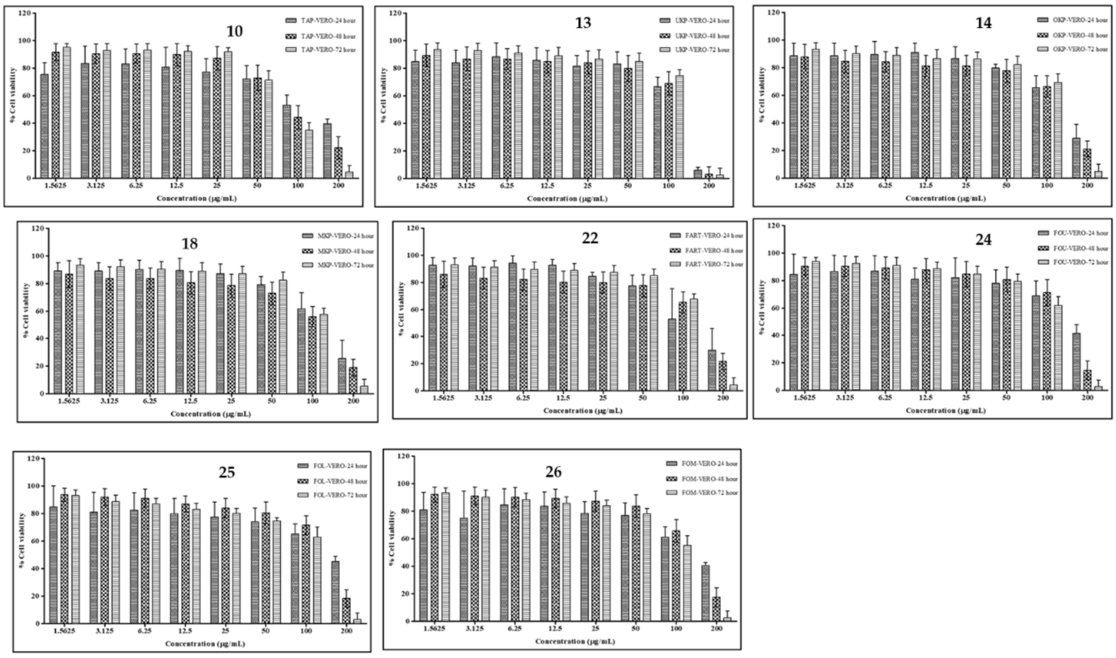


The % Cell viability of hybrid (**10**, **13**, **14**, **18**, **22**, **24**, **25**, **26**) against normal African green monkey kidney (VERO) cells.

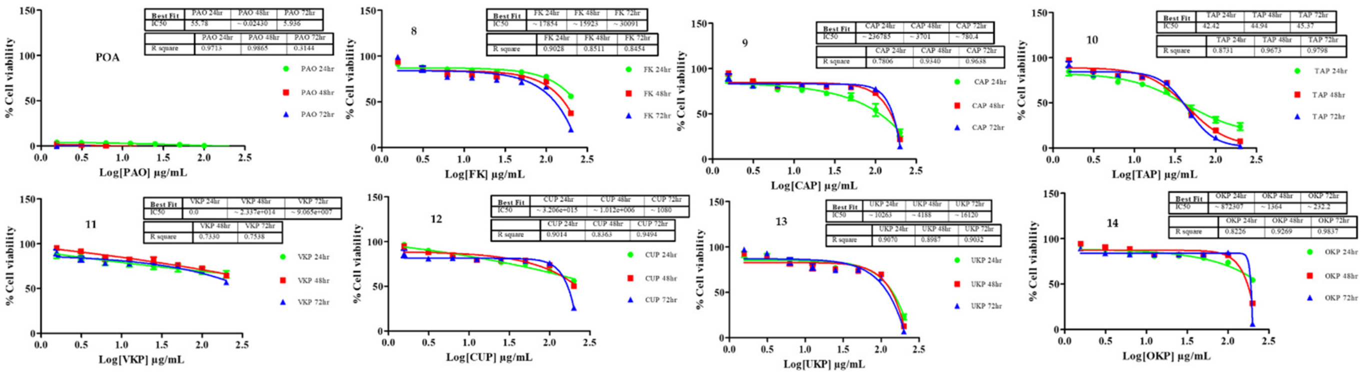


IC50 (μg/mL) values of hybrid compounds (**9**-**14**) together with the positive control (POA) and Negative Control (**16**) against the cervical cancer cell line (HeLa).

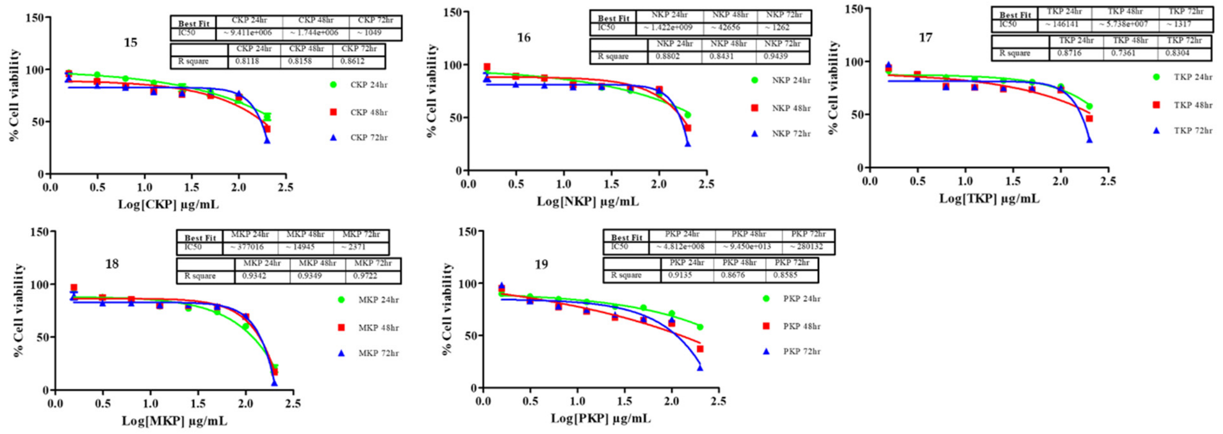


IC50 (μg/mL) values of hybrid compounds (**15**-**19**) against the cervical cancer cell line (HeLa).

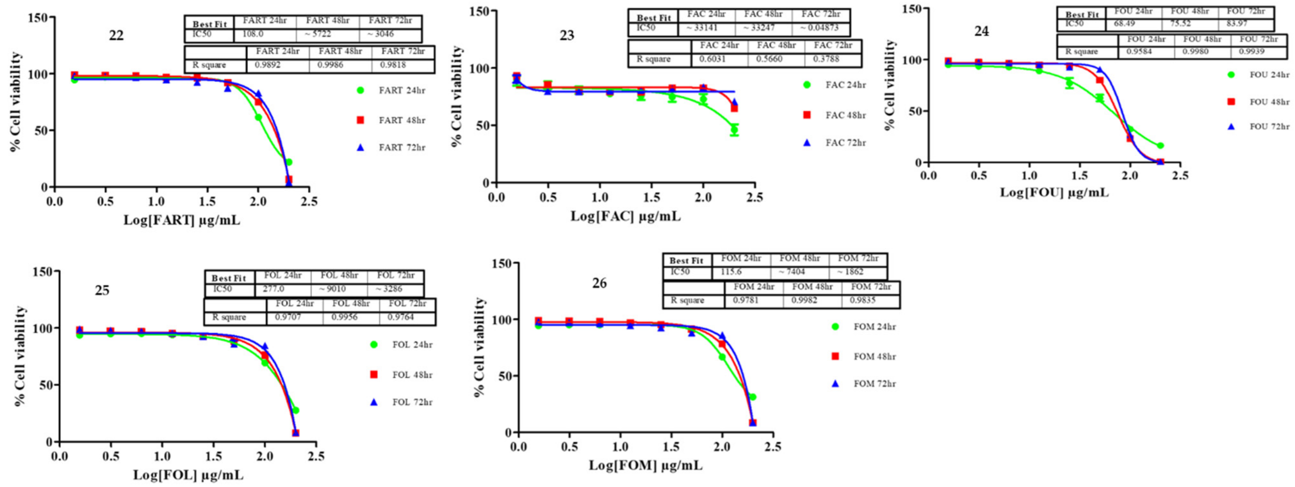


IC50 (μg/mL) values of hybrid compounds (**22**-**26**) against the cervical cancer cell line (HeLa).

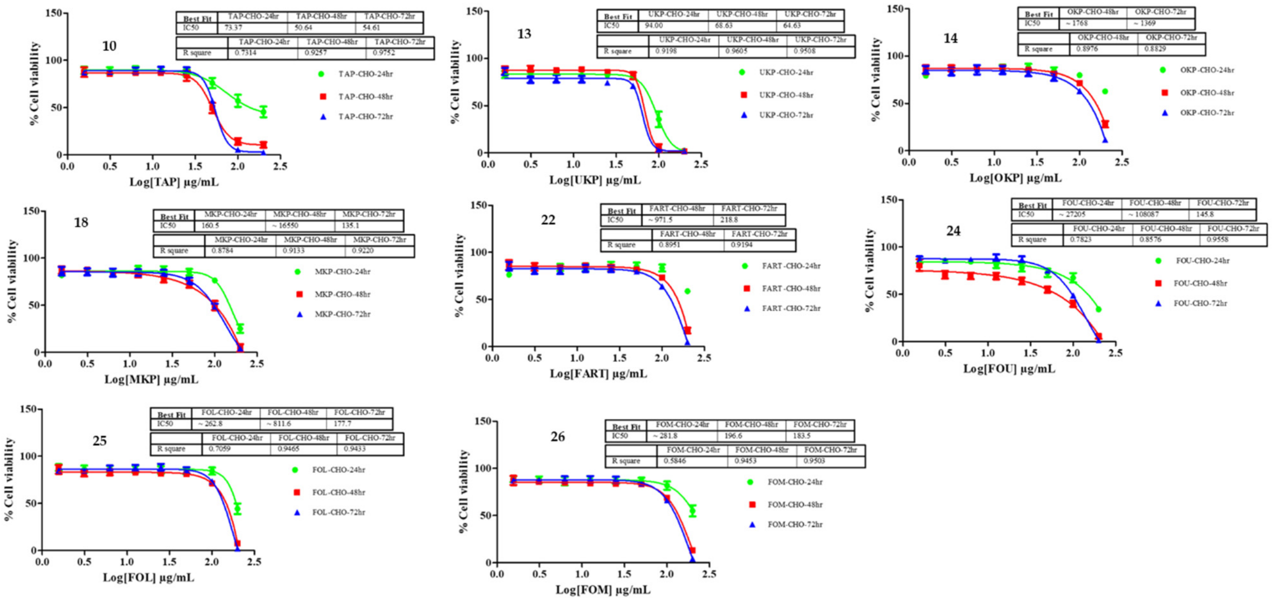


The IC50 (µg/mL) values of hybrid (**10, 13, 14, 18, 22, 24, 25,** and **26**) against Chinese Hamster Ovary cancer cells (CHO).

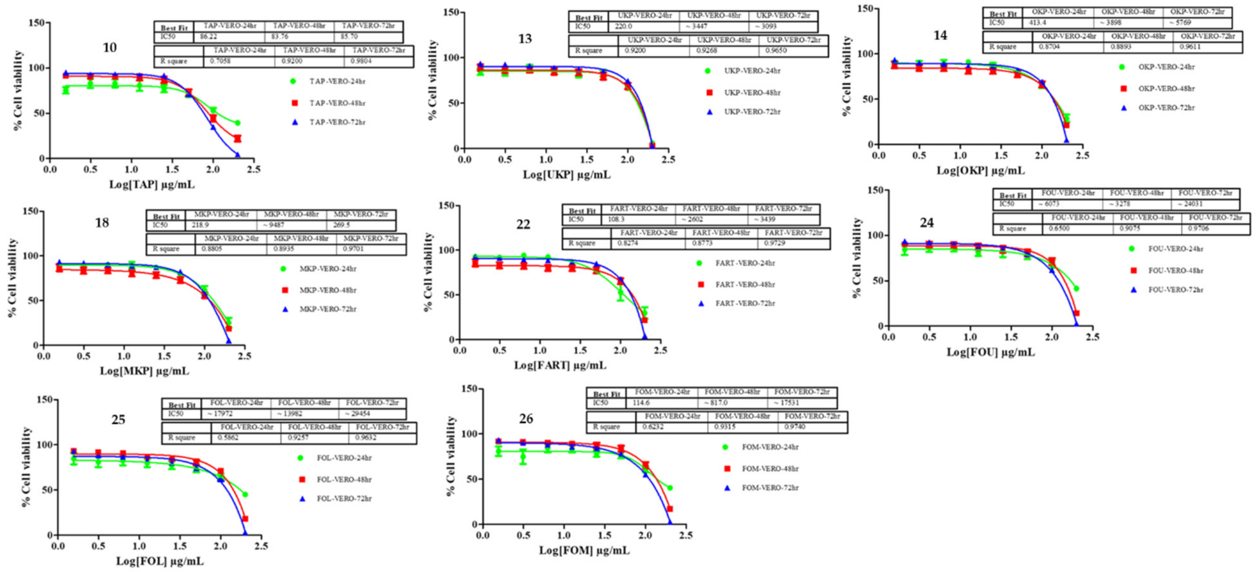


The % Cell viability of hybrid (**10, 13, 14, 18, 22, 24, 25,** and **26**) against normal African green monkey kidney (VERO) cells.
